# Interaction of Carbon Dots with Nucleic Acids Is Driven by Their Surface Charge

**DOI:** 10.1021/acs.jcim.5c02242

**Published:** 2025-12-19

**Authors:** Andrea Nedělníková, Petr Stadlbauer, Pavel Banáš, Jiří Šponer, Michal Otyepka, Petra Kührová, Markéta Paloncýová

**Affiliations:** † Regional Center of Advanced Technologies and Materials, Czech Advanced Technology and Research Institute (CATRIN), Palacký University Olomouc, Šlechtitelů 27, 779 00 Olomouc, Czech Republic; ‡ Department of Physical Chemistry, Faculty of Science, Palacký University Olomouc, 17. listopadu 12, 771 46 Olomouc, Czech Republic; § IT4Innovations, VŠB − Technical University of Ostrava, 17. listopadu 2172/15, 708 00 Ostrava-Poruba, Czech Republic; ∥ Institute of Biophysics of the Czech Academy of Sciences, Královopolská 135, 612 00 Brno, Czech Republic; ¶ Faculty of Electrical Engineering and Computer Science VŠB − Technical University of Ostrava, 17. listopadu 2172/15, 708 00 Ostrava-Poruba, Czech Republic

## Abstract

Carbon dots (CDs) are nanoscale carbon materials with tunable optical properties, low toxicity, and modular functionalization, making them a promising material for biomedical applications. For safe and efficient applications in theranostics, it is essential to assess how CDs interact with biomolecules. Here, we focus on the effect of CDs on the structure and function of nucleic acids (NAs), relevant to NA structural stability, chromatin organization, and gene regulation. We performed more than 150 μs of atomistic molecular dynamics simulations, encompassing a diverse set of NA structures, from canonical DNA and RNA helices through noncanonical motifs such as tetraloops and G-quadruplexes, up to nucleosomes. We simulated their interactions with graphitic CDs with two sizes and distinct surface chemistries: neutral hydrophobic (CD^0^), negatively charged (CD^–^), and positively charged (CD^+^). We identified multiple nonspecific interaction modes including stacking to bases, CH−π contacts, and electrostatic interactions with the NA backbone. All CD types formed contacts with NAs, but only CD^+^ remained tightly bound and is therefore relevant for NA-related applications. The CD nonspecific binding did not compromise the global NA architecture, and we did not observe any intercalation or base-pair disruption. In the nucleosome, CD^+^ adsorb to DNA and occasionally bridge adjacent DNA gyres, that may alter local chromatin dynamics. In summary, the surface charge and particle size emerged as the key determinants of NA–CD interactions, providing atomistic guidance for the rational design of CDs optimized for theranostic applications.

## Graphical abstract

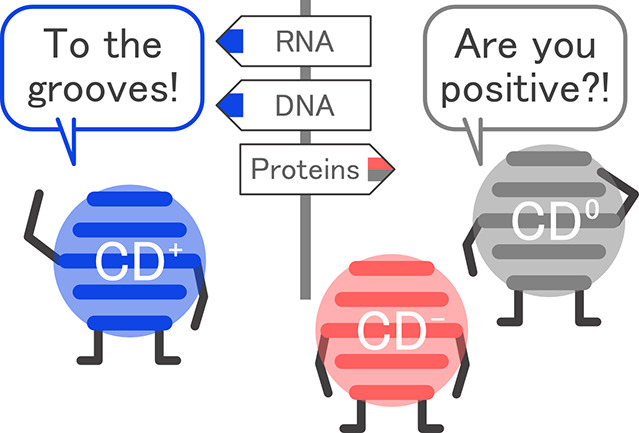

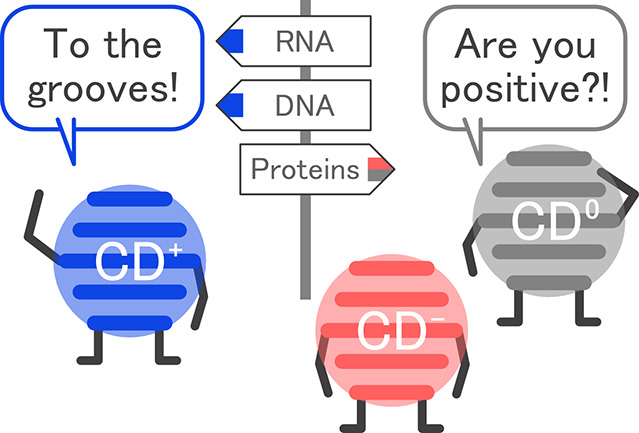

## Introduction

Carbon dots (CDs) are zero-dimensional carbon nanomaterials that have gained significant attention in recent years due to their unique physicochemical properties, including tunable optical characteristics, low toxicity, water solubility, and modular functionalization. Their ability to penetrate biological barriers^1^ makes them highly promising for medical applications, particularly in theranostics.^2,3^ CDs have been studied for their potential in diseases prevention,^4,5^ sensors,^6–9^ vaccination strategies,^10,11^ and bioimaging.^12–16^ Furthermore, they hold promise for photothermal and photodynamic therapies,^17^ gene therapies,^18,19^ and targeted drug delivery.^20–22^ A key advantage of CDs is their tunable luminescence, allowing for excitation wavelength adjustments up to the infrared spectrum, which enables deep tissue penetration for imaging and therapeutic applications.^23^

The biological effects of CDs are largely determined by their chemical and physical characteristics, such as size, hydrophilicity, charge,^24^ functionalization,^25^ etc.^26^ Positively charged CDs are particularly notable for their ability to penetrate cellular organelles, including the nucleus,^24,27–30^ mitochondria,^31^ and endoplasmic reticulum,^32^ while other CDs can preferentially target lipid droplets^33^ or specific enzymes. In vivo, CDs can directly interact with biomolecules, influencing their structure and function.^34^ For example, CDs have been observed to inhibit protein fibrillation,^35^ bind to lipid vesicles,^36^ perform gene therapy,^37^ and alter DNA conformation.^38^ Given their nuclear localization, it is essential to assess how CDs influence nucleic acid (NA) structure and function, as these interactions may impact DNA stability, chromatin organization, and gene regulation. Understanding these effects is crucial for safe biomedical applications, particularly in gene therapy, drug delivery, and epigenetic modulation. However, experimental methods often lack the necessary atomistic resolution to fully characterize CD-NA interactions, making computational approaches, such as molecular dynamics simulations, essential for gaining deeper insights into their biological implications.

Computational approaches offer simultaneous atomic and femtosecond resolution, enabling detailed examination of bio-nano interactions.^39^ While CDs have been simulated in various biological environment,^40^ there are currently few studies focusing on their effect on NAs. For instance, adsorption of single-stranded DNA (ssDNA) on graphitic CDs has been shown to increase with decreasing CD oxidation.^41^ Aggregates of graphene flakes resembling graphitic CDs were shown to interact with DNA in the minor groove,^42^ while larger CDs tend to stack on the terminal bases.^43^ In these simulations, CD-like aggregates did not intercalate into DNA, behavior observed for single-layer polyaromatic hydrocarbons,^42,43^ or known DNA intercalators such as ethidium bromide,^44,45^ berberine,^46^ or doxorubicin.^47,48^ Yet, computational studies of CD interactions with DNA of structural complexity beyond the double helix or with RNA of any kind are lacking. Gaining atomistic-level insight into CD-induced structural changes in RNA and DNA is essential to ensure their safe application in bioimaging,^12–16^ theranostics,^2,3^ and drug delivery.^20–22^ Furthermore, simulations performed in more complex biomolecular environments can reveal the preference of CDs for individual species characterized by their chemical nature, hydrophilicity, charge etc. Such knowledge could enable the targeted design of safe nanomaterials for bioimaging and drug delivery.

In this work, we performed over 150 μs of molecular dynamics (MD) simulations to investigate the interaction of spherical graphitic CDs with NAs. We focused on the smallest CDs (∼1.6 nm in diameter) due to their high potential for deep tissue and cell nucleus penetration. Three CD variants with different surface functionalization were studied: hydrophobic unfunctionalized CD (CD^0^), and CD functionalized by negatively charged carboxyl groups (CD^–^), or positively charged amino groups (CD^+^), as shown in Figure 1. To evaluate the role of CD size, we prepared a positively charged model CD7^+^ (∼2.4 nm in diameter). To probe the interactions of all types and sizes of CD with NA, we selected a representative set of NA structures encompassing a wide range of biological and structural contexts (Figure 1). These included canonical double-stranded DNA and RNA helices, noncanonical motifs such as a GAGA RNA tetraloop (TL) and G-quadruplexes (G4), a hybrid G4-duplex junction (QDJ), and a protein–DNA complex represented by the nucleosome (NS). This progression from free short helices to compact and highly organized chromatin structures allowed us to investigate how CD binding is modulated by NA topology, flexibility, and protein context.

**Figure 1:**
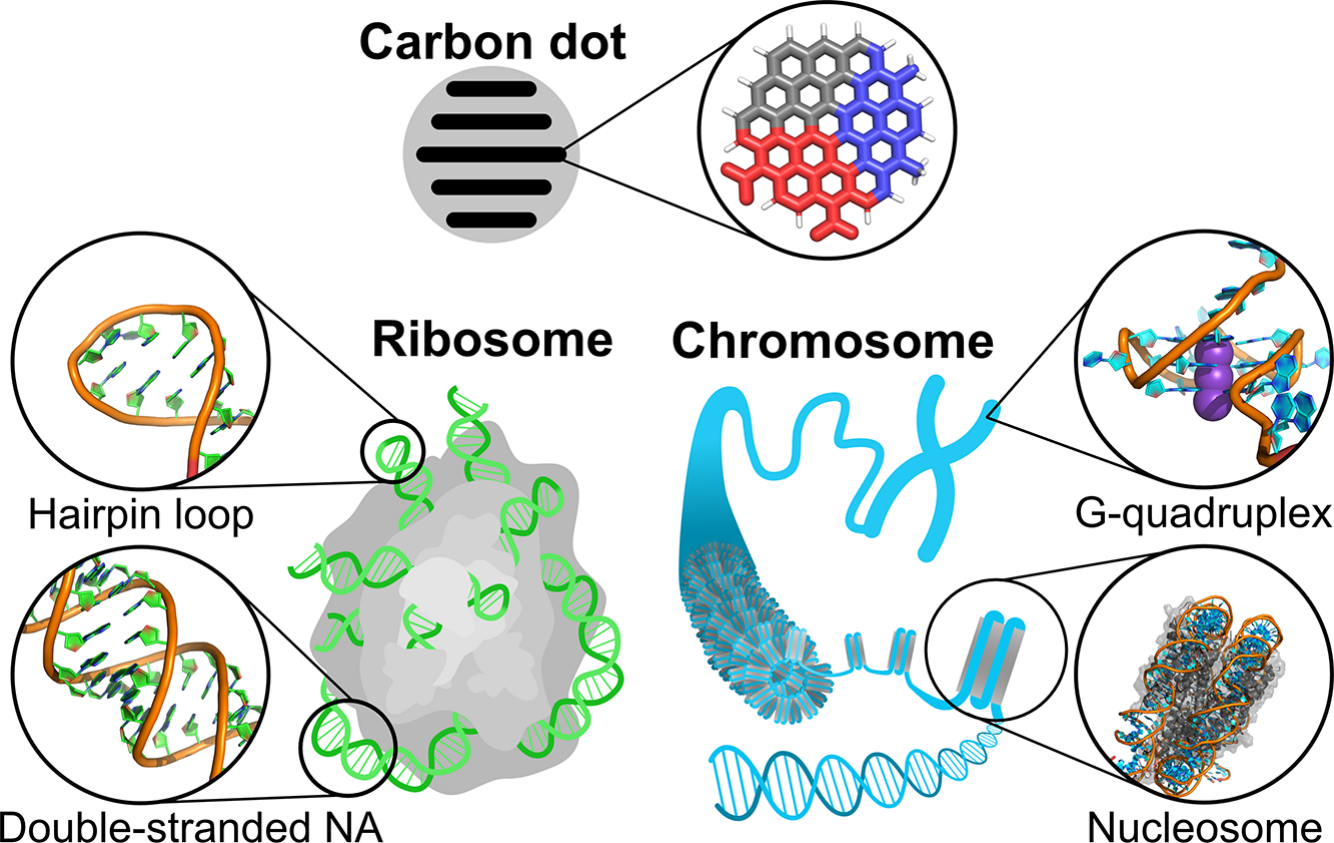
Overview of the studied systems. Carbonized CD with a graphitic core is schematically shown in a cross-section with a highlighted functionalized polyaromatic hydrocarbon building block. The color scheme corresponds to the colors used in other figures of the manuscript: neutral CD^0^ are depicted in gray, negative CD^–^ in red, and positively charged CD^+^ in blue. RNA systems have nucleobases shown in green, DNA in cyan, and proteins are depicted as gray surfaces.

## Methods

### Preparation of Starting Structures of Biomolecules

The set of biomolecules comprises various RNA and DNA structures to cover a diverse range of NA conformations and sequences. As canonical RNA structures, four double helices were considered: (i) an alternating AU sequence, r­[U­(UA)_6_A] (PDB ID: 1RNA,^49^ denoted as R14AU); (ii) a segment of 19 base pairs with the sequence r­(GCAC­CGUU­GGUA­GCGG­UGC) (extracted from PDB ID: 1QC0,^50^ denoted as R19N); (iii) RNA duplex r­(CG)_5_, featuring alternating CG base pairs constructed using the Nucleic Acid Builder (NAB) tool of AmberTools^51^ (denoted as R10CG); (iv) r­(CG)_10_ duplex built up the same way (denoted as R20CG). Furthermore, two canonical B-DNA double helices were studied: (v) Dickerson-Drew dodecamer (PDB ID: 1BNA,^52^ denoted as D12N), and (vi) a DNA duplex d­(CG)_10_ constructed in NAB (denoted as D20CG). The noncanonical structures included: (vii) RNA GAGA tetraloop with a short stem (PDB ID: 1Q9A,^53^ residues 2658–2663, denoted as TL); (viii) DNA c-Myc G4 (PDB ID: 6AU4,^54^ denoted as cG4); (ix) the human telomeric DNA parallel-stranded G4 (PDB ID: 1KF1, denoted as hG4); (x) G4-duplex junction structure from the promoter of the human PIM1 gene (PDB ID: 7CV3), from which we removed the overhang bases and the third G4 loop (nucleotides T23 and C24) to expose the quartet to the solvent (denoted as QDJ); and finally (xi) nucleosome containing the human H3T histone variant (PDB ID: 3AFA,^55^ denoted as NS).

The starting structure of NS was taken from the 3AFA structure. The protonated states of amino acids were set in agreement with pH 7.3 using the H++ (see Table S8 for a list of histidine protonation-state and tautomer assignments).^56^ The force field details are described later in the section Building of Systems Containing CDs.

### Preparation of CDs

The CDs utilized in this study were constructed using the Carbon Dot Builder^57^ plugin, integrated within VMD^58^ software. Each CD was designed with five distinct layers with a total height of 1.74 nm. The three central layers were configured with three aromatic nuclei at their periphery, each featuring a diameter of 1.6 nm (circumcoronene), while the two outermost layers contained only two aromatic nuclei each (coronene) to keep the approximately spherical shape. To evaluate the effect of charge and functionalization on the properties of CDs, three types of CDs were prepared: a positively charged CD incorporating nine protonated NH_3_^+^ groups (referred to as CD^+^); a negatively charged CD featuring 11 deprotonated COO^–^ groups (referred to as CD^–^); and a neutral CD without any functional group modification (referred to as CD^0^), which served as a control for comparative analysis. The desired level of functionalization of the CD^+^ and CD^–^ surfaces was chosen to be ∼10% of carbon atoms at CD flake edges. The variation in the number of functional groups is due to the randomness element inherent in the CD builder. Additionally, to investigate the effect of a larger positively charged CD, a seven-layered CD (denoted as CD7^+^) was simulated (see ). This CD7^+^ featured an increased height of 2.4 nm and carried a ∼10% coverage with functional groups, amounting to a total of 26 NH_3_^+^ moieties. The layered structure was composed of seven stacked aromatic planes: two outermost layers contained three aromatic nuclei at their edge (i.e., circumcoronene), the adjacent layers contained four, and the three central layers were constructed with five nuclei at their edge. We chose NH_3_^+^ functionalized CD as an idealized model of positively charged CD with uniformly and highly charged surface to isolate and investigate the effects of electrostatic interactions while minimizing contributions from uncharged or hydrophobic regions.

### Building of Systems Containing CDs

The building of all simulated complexes followed a similar protocol: The starting structures of biomolecules were taken as described above, except for NS, for which we took a structure after 200 ns of MD simulation (see the next section). Then, the biomolecule starting topologies were prepared independently using the AMBER package, while the CDs were generated using the GROMACS package, by the procedure from originally published builder.^57^ Subsequently, the structures of the CDs underwent energy minimization, were integrated with the exposed biomolecules (by GROMACS tool insert-molecules), and the topologies were merged and converted to the AMBER format by ParmEd.^59^ Each simulated complex contained one biomolecule and one CD and was prepared in four replicates, with the exceptions of QDJ, which was simulated five times with CD^0^ or CD^–^ and ten times with CD^+^, and the complex containing the NS. CD7^+^ was tested with R14AU, R19N and D12N canonical systems, run with three, two, and three replicates, respectively. Further, we simulated CD7^+^ with cG4, hG4, and QDJ, all in three replicates. NS simulations were performed in several sets containing more CDs to increase sampling. First set included 5 CDs and was simulated once with CD^0^ and twice with CD^–^ or CD^+^. To evaluate the role of CD concentration, we also performed simulations which contained three and eight CD^+^, respectively. Furthermore, we simulated NS in three replicates with five CD7^+^.

The complete systems were then solvated by combining the OPC explicit water model^60^ with 0.15 M KCl, using Joung-Cheatham ion parameters.^61^ The RNA-containing systems were simulated using the AMBER RNA force field parmbsc0 ^62,63^ with the OL3 modification^64^ combined with van der Waals modifications for phosphate oxygens.^65^ Additionally, the external generalized interaction-specific gHBfix19 potential was applied:^66,67^ (i) all NH-N hydrogen bond interactions were strengthened by 1.0 kcal/mol, and (ii) all OH-nbO hydrogen bond interactions were weakened by −0.5 kcal/mol. The DNA-containing systems were simulated with the AMBER DNA force field OL21,^68^ encompassing the χ_OL4_,^69^ εζ_OL1_,^70^ β_OL1_ ^71^ and α/γ modifications^68,72^ of the Cornell et al. force field.^63^ In NS, the ff12SB force field was used for proteins.^73^

The selected nucleic acid force fields represent the current state-of-the-art for unbiased MD of DNA and RNA.^74^ For DNA, OL21 ^62,63,68–71^ has recently been shown to outperform all earlier AMBER variants (including bsc1,^75^ OL15 ^71^ and Tumuc1 ^76^ across a broad range of B- and Z-DNA systems, providing the most accurate backbone, helical parameters, and dynamical behavior.^77^ For RNA, the OL3 force field combined with the phosphate-oxygen van der Waals modifications and the interaction-specific gHBfix19 potential (OL3_CP_-gHBfix19)^64–67^ is a well-established and computationally efficient variant that has consistently improved RNA tetranucleotide and tetraloop ensembles while avoiding side-effects observed in some alternative reparameterizations.^66,67,78,79^ Using these two force fields therefore provides a robust and widely validated description of the nucleic-acid structure and dynamics for the present study.

### Simulation Protocol

Prior to MD simulations, each system underwent a series of minimizations and equilibrations. Initially, water molecules and ions were relaxed while keeping the positions of the solute constrained, allowing the solvent molecules and counterions to move during a 1000-step minimization. This was followed by a 500 ps MD simulation under NpT conditions (1 atm, 298 K) to relax the box volume. After this initial procedure, the biomolecule underwent multiple minimization runs, during which decreasing force constants were applied to the sugar–phosphate backbone atoms. The system was then heated in two steps: first under NVT conditions for 100 ps, followed by equilibration under NpT conditions for an additional 100 ps.

Electrostatic interactions were managed using the particle-mesh Ewald (PME) method under periodic boundary conditions in the NpT ensemble at 298 K. Temperature control was maintained using the weak-coupling Berendsen thermostat^80^ with a coupling time of 1 ps. The SHAKE algorithm, with a tolerance of 10^–5^ Å, was used to constrain the positions of all of the hydrogen atoms. A 10.0 Å cutoff was applied to nonbonding interactions to enable a 2 fs integration step.

All biomolecules were simulated in combination with CD^0^, CD^+^, and CD^–^ (see Tables S3–S5). To account for variations in relative positioning, each system, excluding D20CG, R20CG, was prepared with different random positions of CD in bulk solvent in several replicates. For D20CG and R20CG, ten simulations were performed with CD^+^ initially placed near either the major groove (five replicates) or the minor groove (five replicates), to sample different binding orientations. Additionally, selected biomolecules and CDs were simulated individually as reference systems (see Tables S1 and S2 for an overview of the performed simulations). The length of the simulations varied between 0.4 and 2.4 μs (see Tables S1–S7 for the system composition and simulation length).

### Description of Performed Analyses

The global behavior of all systems was analyzed using the *cpptraj* module (AMBER tool).^81^ The program was used for evaluation of NA structure (root-mean-square deviation of atomic positions, dihedral backbone angles, sugar pucker, and helical parameters) and interaction of CDs with NA (monitoring of distances between atoms, radial distribution functions between CD and residues in contact, and number of hydrogen bonds). The time evolution of CD-NA interactions was monitored by solvent accessible surface area (SASA) calculated by *gmx sasa* from GROMACS package.^82,83^ SASA was calculated for (a) CD, (b) NA (and histone in nucleosome), and (c) complex of CD, NA (and histone), with a probe radius of 0.14 nm. The difference of SASA of the complex and sum of individual components is presented as a measure of contact surface area, system time evolution, and convergence. The distance of NS gyre centers was calculated as the closest distance between a center of mass of a base pair and another base pair (excluding ten closest base pairs in DNA sequence). Some of the per-residue properties were visualized in PyMOL^84^ as per-residue color scale. Local structural effects of binding of CD^+^ to nucleosomal DNA were quantified using an event-aligned RMSD analysis at the residue level. A detailed description of the procedure is provided in the Supporting Information. Density maps of CDs in contact with NAs were calculated and visualized using VIAMD software.^85^ The overall shape and behavior of CDs was described using several parameters: interlayer distances, undulation of layers, horizontal positions of individual layers, rotations (Figure S1), and tilting of neighboring layers relative to the reference middle layer. The evolution of these parameters was analyzed throughout the entire simulation, with average values, densities, and other metrics calculated from the last 200 ns of the simulation.

## Results and Discussion

We performed 151 all-atom simulations to investigate the interactions between CDs and nucleic acids (NAs). Our study we focused on small (∼1.6 nm in diameter) carbonized CDs with a graphitic core, which easily permeate cellular structures. To assess the role of CD hydrophobicity and surface charge in CD–NA interactions, we designed three CD functional modifications. Nonfunctionalized graphitic CD^0^ served as a hydrophobic reference, CD^–^ with its COO^–^ groups represented a negatively charged variant, while CD^+^ with its NH_3_^+^ groups represented a positively charged one. These surface modifications are commonly introduced in synthetic CDs and are relevant for biomedical applications due to their biocompatibility.^86^ The larger positively charged CD7^+^ was included in the study to evaluate the role of the CD size.

To evaluate how CDs influence NA behavior, we performed unbiased simulations with a representative set of NA structures covering different biological contexts. Specifically, we examined canonical double-stranded RNA and DNA helices, noncanonical motifs (), such as an RNA tetraloop (TL), DNA G-quadruplexes (G4), G-quadruplex-duplex junction (QDJ), and a protein–DNA complex represented by the nucleosome (NS). The systematic selection of NA structures probes the CD interactions across a diverse structural context, i.e., from regular helices, through noncanonical elements, to densely packed chromatin. This approach allowed us to assess the CD binding modes, structural effects, and potential implications for NA stability and cellular function.

### Interaction Modes between CDs and Canonical NA Helices

Initially, we probed the interactions of CDs with canonical helices, which are common motifs in NAs. In genomic DNA, B-DNA helices predominate, while, in cellular RNAs, shorter A-RNA helices constitute a substantial portion of overall structures. In simulations of CDs with these canonical helices, we identified two principal interaction modes: I) interactions with grooves, either minor or major, and II) interactions with terminal base pairs (Figures 2, S3 and S12–S16 and Tables S3, S5, and S6).

**Figure 2:**
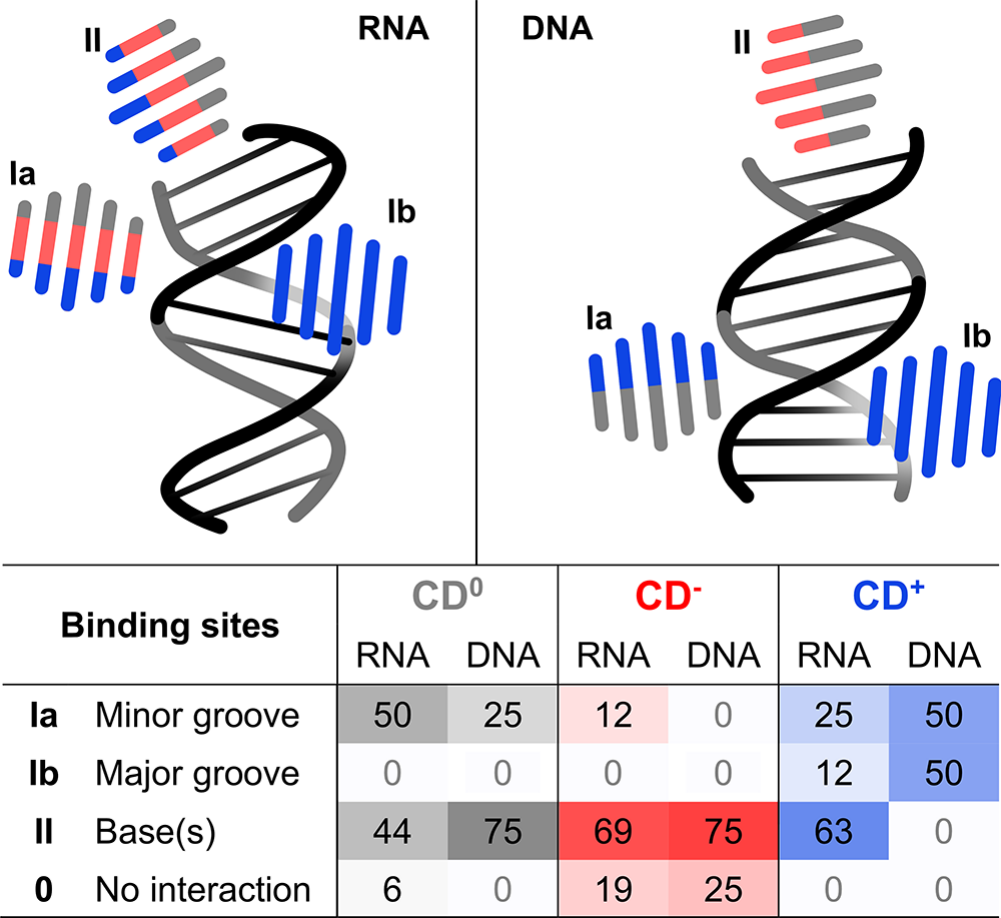
Schematic representation (top panel) illustrates the interaction modes of CDs with canonical helices. The color of each CD symbol indicates the type of CD involved in the interaction: CD^0^, CD^–^ and CD^+^ are shown in gray, red, and blue, respectively. The table summarizes the percentage of simulations that resulted in the given interaction mode (for more details, see Tables S3 and S5 and Figures S12–S15). Binding modes for CD7^+^ are listed in Table S6.

The preferred interaction mode varied depending on the type of NA and specific CD functionalization. Although binding to terminal base pairs was generally the dominant mode in these simulations, it is likely less relevant in a cellular context where internal base pairs, helices, and grooves are more prevalent. The only systems not preferring binding to NA terminal bases were CD^0^ with RNA and CD^+^ with DNA. We observed that CD^0^ preferentially engaged the minor groove of RNA helices, while CD^+^ interacted with the minor and major grooves of DNA with comparable frequency (Figure 2). Below, we describe these interaction modes and their effects on NA structures in more detail.

#### Interaction with Minor and Major Grooves

Interactions with the minor (Ia) and major (Ib) grooves were common for CD^0^ and CD^+^, but they were rather rare for CD^–^. CD^–^ engaged the RNA minor groove in only 12% of cases, typically positioning over the ribose rings and avoiding contact with the phosphate backbone, likely due to electrostatic repulsion (Figure 2). CD^0^ and CD^+^ instead interacted not only with the ribose rings but also maximized contacts with negatively charged phosphates of the backbone (for CD^+^; Figure S17) and the sugar edges of nucleobases (in the case of CD^0^), preferentially interacting in the minor groove (Figure S4).

This binding mode preference was particularly notable for DNA, where CD^+^ interacted exclusively in the minor or major groove (Figures 2 and S15), benefiting from electrostatic attraction and hydrogen bonding with the sugar–phosphate backbones. These observations support that groove binding is dominantly driven by electrostatics, making CD^+^ the predominant interaction partner for NAs, whereas CD^–^ is more likely to associate with other cellular structures, thus avoiding repulsion from the polyanionic NA chain.

To probe the effect of CD size on groove binding, we performed a set of simulations with a larger positively charged CD, denoted as CD7^+^. As expected, the larger CD7^+^ did not insert deeply into the NA grooves. Instead, it interacted more superficially, namely, with both grooves in RNA and predominantly the minor groove in DNA, forming hydrogen bonds with the sugar–phosphate backbone near the outer surface of the helix (Figure S16).

To assess the effect of CD binding on the NA structure, we focused on CD^+^ interactions within the major and minor grooves, as this binding mode is considered the most biologically relevant. We systematically designed a set of 20-mer simulations: ten replicates of CG DNA (D20CG) and ten of CG RNA (R20CG) duplexes with CD^+^ molecules initially positioned near either the major or minor groove (five simulations per groove for each NA type). This setup enabled us to sample various positions of CD^+^ within the grooves and study its influence on the NA structural dynamics.

We observed major groove binding in four D20CG and five R20CG simulations and minor groove binding in six D20CG and three R20CG simulations. In two R20CG simulations, the CD^+^ molecules migrated toward the terminal base pair. The CDs generally remained anchored at their initial binding sites, exhibiting minimal rotation and positional drifts, indicating that these interaction sites are relatively stable (Figures S21 and S23).

Binding of CD^+^ within the grooves caused only moderate local perturbations of NA helical structure, without disrupting the overall helical integrity (Figures 3 and S5–S8). Structural changes were more pronounced upon binding to the major groove, particularly in the propeller twist parameter (Figures 3, S5, and S6), and extended over a broader region in RNA rather than in DNA. In contrast, minor groove binding induced only small, localized shifts in helical parameters, most notably in the shift parameter value in RNA, while DNA remained largely unaffected (Figures 3 and S7). These localized deformations likely reflect the geometric compatibility between the size of used CDs and the dimensions of the NA grooves allowing them to fit within the groove and directly interact with the groove interior. Such a specific effect is less likely to occur with CDs of larger diameters as observed in CD7^+^ simulations (see above). In some cases, the affected helical parameters partially reverted toward their original values over time (see Figure 3, RNA minor groove binding), indicating a degree of structural resilience and suggesting that groove-bound interactions do not irreversibly distort the NA conformation.

**Figure 3:**
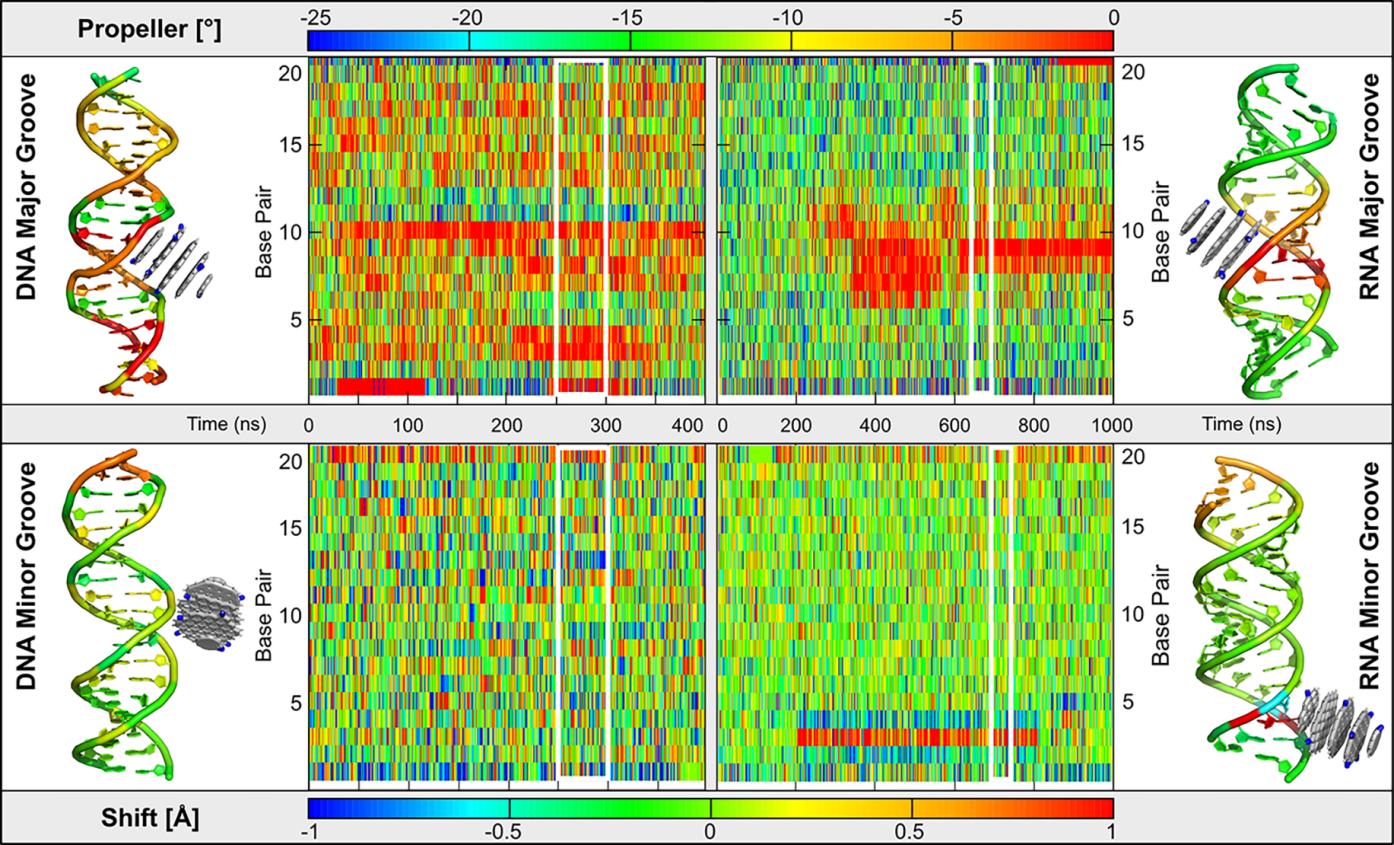
Heatmaps showing the time evolution of propeller twist (top) and shift (bottom) for DNA (left) and RNA (right) duplexes with a CD bound in the major (top) or minor (bottom) groove. Colors indicate the magnitude of each parameter per base pair over time. The plots highlight that local structural changes are more profound in RNA. Side panels show representative structures with the CD in the corresponding groove colored by the per-residue average of propeller twist and shift calculated from the highlighted part of the simulation (indicated in white rectangles). The displayed parameters were the most affected by the CD-attachment (for the evolution of both helical parameters for both grooves together with buckle parameter, see Figures S5–S8).

#### Interaction with Terminal Base(s)

Stacking onto terminal bases (II) represented the most common binding mode in our simulations (Figures 2, S3, S9, and S10). These interactions occurred rapidly for both CD^0^ and CD^+^ (Tables S3 and S5 and Figures S18–S24) and were stabilized by stacking between aromatic surfaces of the exposed terminal base pairs and graphitic layers of CDs, typically at a stacking distance of ∼0.34 nm. The contact surface area between CD stacked on the terminal bases and NA was smaller than that in the case of CD binding to the NA grooves (Table S9). While this binding mode was frequently observed in our simulations of short duplexes, it is likely less relevant in cellular nucleic acids, where terminal base pairs are relatively rare. However, the ability of CDs to stack to exposed bases may still play a role in binding to noncanonical NA motifs, which often present unpaired or bulged-out nucleotides (see next section).

To engage in stacking interactions with NAs, CD^–^ required substantial deformation. In contrast to CD^0^ and CD^+^, which largely retained their spherical shape, in some cases CD^–^ underwent rearrangement of its graphitic layers, exposing hydrophobic core regions to enable contacts with nucleobases (Figures S9 and S12–S15). This process sometimes involved sliding or partial dissociation of the smallest outer layers, resulting in a more elongated structure with a larger exposed surface area (Figure S11 and Tables S3 and S5), similar to what has been observed in MD simulations of CDs in pure water.^65^ In addition, CD^–^, unlike CD^0^ and CD^+^, was also able to weakly bind counterions from bulk solvent. These observations suggest that CD^–^ pays a higher energetic cost to interact with NAs, further supporting the conclusion that in the cellular environment, it is more likely to bind alternative, more favorable targets.

### Interaction Modes between CDs and Noncanonical NA Structures

The frequent occurrence of stacking interaction between CDs and terminal base pairs in canonical helices suggests that CDs tend to associate with exposed nucleobases via stacking, which might play a key role in noncanonical regions. To further explore this behavior in structurally diverse environments, we investigated the interactions of CDs with three representative noncanonical NA motifs: an RNA tetraloop (TL), two G-quadruplexes (hG4 and cG4), and G-quadruplex-duplex junction (QDJ). TL is a prevalent RNA motif that caps double-stranded helices and functions as a strand reversal element. G4 structures, on the other hand, are formed by guanine-rich sequences that fold into guanine tetrads stabilized by Hoogsteen hydrogen bonds and monovalent cations. These structures are more rigid and play critical roles in gene regulation, especially within telomeric and promoter regions.^87^ In regions of the chromatin chain where nucleosomes are depleted, such as active regulatory domains or telomeres, G-quadruplex structures can form and modulate local chromatin architecture.^87^ The QDJ presents a unique structural feature: a pocket that could be selective for small molecules binding to G4 and duplex DNA simultaneously.

Across these systems selected for their distinct architectures, we observed several types of interaction modes (Figure 4 and Tables S4 and S7): (I) interaction with sugar–phosphate backbone; (IIa) binding to unpaired bases located in TL hairpin, G4 loops, or overhangs; (IIb) stacking onto terminal base pairs; and (IIc) stacking onto exposed G-tetrad. The specific interactions observed for each NA noncanonical motif are described in the following paragraphs.

**Figure 4:**
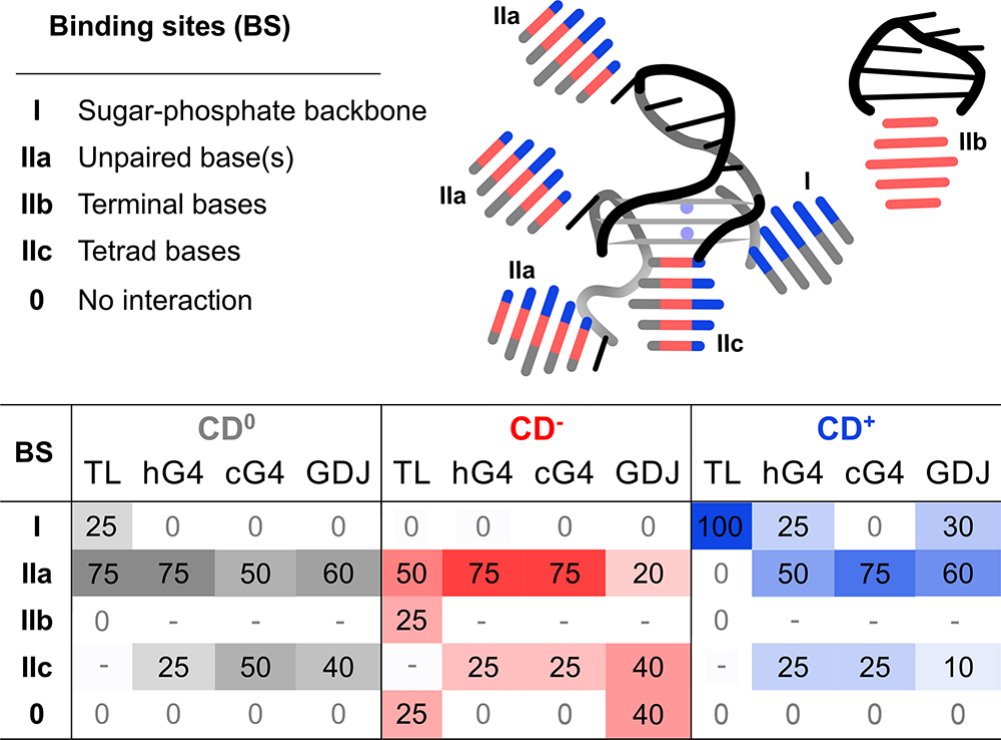
Schematic representation (top panel) shows the interaction modes of CDs with noncanonical NA structures. The color of the CD symbol represents the type of CD involved in the interaction: CD^0^, CD^–^, and CD^+^ are depicted in gray, red, and blue, respectively. The table summarizes the percentage of simulations that resulted in the given interaction mode. A dash ‘-’ indicates that the interaction mode is not applicable for the given NA motif. Binding modes for CD7^+^ are given in Table S7.

#### Tetraloop

CD^0^ and CD^–^ tended to interact with the TL via their graphitic face, either by stacking onto exposed unpaired loop nucleobases (Figure 4, IIa) or by stacking onto the terminal base pair (IIb). CD^–^ also showed additional contacts with the base edges and ribose rings (I). CD^+^ interacted similarly with base edges and ribose rings (I), but also frequently bound directly to the phosphates via its protonated amino groups (I). The overall structure of TL remained intact upon CD binding (Figure S25).

#### G-Quadruplexes

We selected hG4 and cG4 as representatives of biologically relevant G4s, chosen for their unique structural features. Both contain a large, solvent-exposed planar surface on their terminal tetrads, which is generally favorable for stacking interactions.

In hG4, the most frequent interaction mode involved stacking onto unpaired loop bases (Figure 4, IIa), followed by interaction with G-tetrads (IIc), and in the case of CD^+^, also the sugar–phosphate backbone (I). In cG4, we observed similar binding modes, however, stacking to overhanging bases surpassed the frequency of the interactions with G4 loops. Also, the overhangs sterically obstructed G-tetrads, likely hindering their interaction with CDs. Representative structures of the binding modes are shown in Figure 5 and all final binding modes are shown in Figures S26 and S27. Interestingly, in the case of stacking onto the G-tetrad, the CD layer directly in contact with the tetrad was not aligned with the G4 channel, but was off-centered (Figure S28).

**Figure 5:**
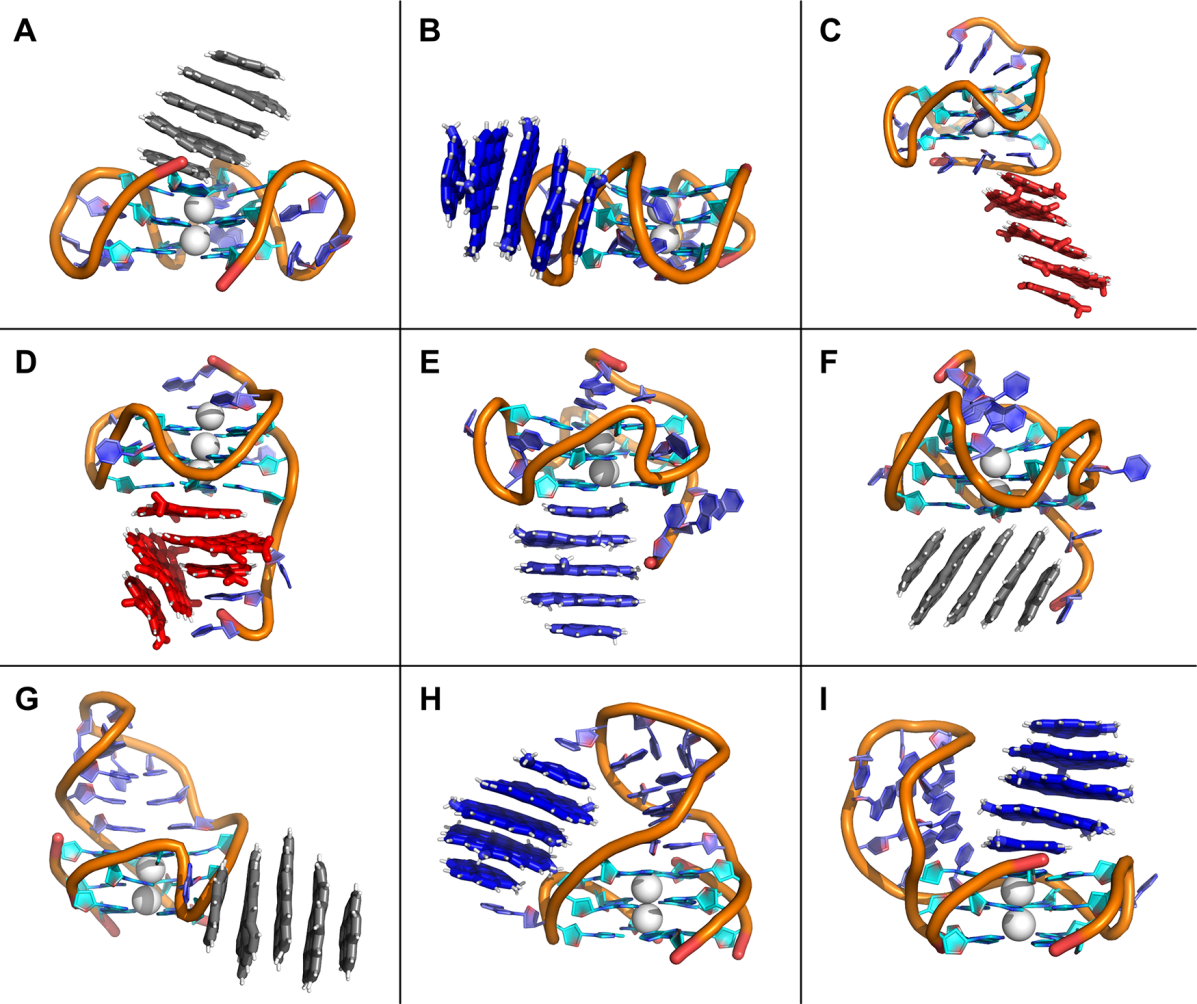
Representative snapshots of CD binding modes with G-quadruplexes (G4s) and the G4-duplex junction (QDJ). Panels A–F show various interaction modes of CDs with hG4 and cG4. (A) CD^0^ stacking onto hG4 tetrad, (B) CD^+^ interaction with both the backbone and the loop region of hG4, (C) CD^–^ stacking onto an overhang base that itself stacks on the cG4 tetrad, (D) CD^–^ sandwiched between the cG4 tetrad and overhang, (E) CD^+^ stacking onto the cG4 tetrad while forming hydrogen bonds with the overhang, (F) CD^0^ interacting with the cG4 tetrad and stacking onto the overhang nucleobase. Panels G–I show CD interactions with the G4-duplex junction (QDJ). (G) CD^0^ stacking onto the exposed nucleobase of the QDJ G4 loop region, (H) CD^+^ embedded between exposed nucleobases of QDJ G4 loop and hairpin loop, (I) CD^+^ stacking onto 3′-tetrad of QDJ representing the known binding site of small molecule ligands. G4 stems are depicted in cyan with 5′-tetrad at the bottom; other nucleotides are slate. CD^+^, CD^–^, and CD^0^ are shown as sticks in blue, red, and gray, respectively.

The G4 stem structure remained largely stable upon CD binding (Figures S26 and S27). The integrity of the tetrads was preserved in all simulations, regardless of the CD type or binding site. When a CD stacks directly to a terminal tetrad, a slight reduction in helical twist between adjacent tetrads was observed. Potassium ions remained stably coordinated within the G4 channel and did not exchange with the solvent throughout the simulations.

#### Quadruplex-Duplex Junction

QDJ effectively behaves as a hybrid of G4, DNA duplex, and hairpin loop. We observed that the simulations took a long time to evolve into a stable state (Figure S35), resulting in various interaction modes (Figure 4), including stacking to exposed unpaired nucleobases (IIa), electrostatic binding to the sugar–phosphate backbone in the hairpin region, the G4 grooves and the G4 loop (I), and stacking to G4 tetrads (IIc). Interestingly, the known small-ligand binding site present at the junction between the G4 and the duplex-hairpin was only rarely occupied by CDs (Figures S29 and S30).

The binding was strongly influenced by the CD charge. CD^–^ showed the weakest binding, likely due to electrostatic repulsion from the sugar–phosphate backbone. In two simulations, CD^–^ did not bind QDJ at all (Figures 4, S29, and S36). When binding did occur, it typically involved stacking onto the hairpin loop (IIa) or tetrad bases (IIc). CD^0^ bound more rapidly, predominantly via stacking to unpaired bases in the hairpin and G4 loop (Figure 4 IIa, Figure 5G, and Figure S36). Occasionally, CD^0^ was deformed to increase the level of stacking between its hydrophobic core with the exposed G-tetrad (Figure S29). CD^+^ exhibited the fastest binding (Figure S35). Unlike CD^0^ and CD^–^, it also interacted with the negatively charged backbone (Figure 4, I), often in combination with stacking interactions involving hairpin unpaired nucleobase (IIa). We also observed hairpin and G4 loop bases simultaneously stacked onto opposite sides of CDs (Figure 4 IIa and Figure 5H). CD^+^ was the only variant observed to bind at the small-molecule ligand site in the G4-duplex junction region (Figure 5I). In cases in which the outermost CD layer stacked directly onto the G-tetrad of QDJ, it was off-centered (), similar to its binding to the G4s.

In summary, CD interactions with QDJ and other studied noncanonical NA motifs were diverse but generally nonspecific. CD charge affected the affinity for the NA backbone, while stacking occurred opportunistically at exposed bases. We did not observe any significant size-dependent differences in CD interaction (Figures S26, S27, S30, and S31). Despite expectations that G-tetrads would be a primary binding site due to their large stacking-prone surfaces, loop and overhang regions were more frequently engaged. Even the QDJ structural modification, i.e., removal of a G4 loop resulting in a G-tetrad almost completely exposed to solvent, did not lead to increased CD binding (e.g., compared with cG4, which has terminal overhangs shielding the G-tetrads (cf. Figure 4)). These observations suggest that, unlike some small ligands, CDs may not selectively recognize or stabilize specific G4 topologies. Given the relative scarcity of G4s in the genome compared with double-stranded DNA, and the predicted nonspecific nature of CD binding, we therefore expect CD–G4 complexes to be relatively rare in vivo and unlikely to exert any defined biological function.

Experimental validation of these predictions could include fluorescence-based binding assays, electrophoretic mobility shift analysis, or calorimetric measurements to evaluate the binding specificity and affinity of CDs for G4s versus double-stranded DNA. In-cell fluorescence colocalization imaging may also be used to assess potential interactions of CDs with telomeric G4s.

### Nucleosome

To study a biologically relevant and structurally complex system we selected nucleosome (NS), a fundamental repeating unit of chromatin in eukaryotic cells, consisting of ∼146 base pairs of DNA wrapped ∼1.7 turns around the histone core (Figure S42).^88^ This tight DNA-histone association imposes unique geometrical and mechanical constraints, including sharp bending, groove compression, and overall topological underwinding accompanied by local helical overwinding, resulting in negative supercoiling and an average twist of ∼10.2 base pairs per helix turn - distinct from the canonical B-DNA form (10.5).^88^ The minor groove facing the histone core is significantly compressed, and DNA at histone contact sites exhibits altered rise, roll, and tilt parameters.^89^

Investigation of the CD interaction with NS allows us to evaluate the context of the higher-order organization, structural constraints, reduced accessibility, and protein–DNA interactions that define chromatin dynamics. Additionally, since positively charged CDs are known to penetrate the nucleus,^27^ it is crucial to examine their interactions with nucleosomal DNA and histones to understand their potential impact on chromatin structure. The nucleosome thus represents a crucial model for bridging observation from simplified NA motifs to a realistic chromatin-level environment.

#### Interactions of CDs with NS

The larger size of the NS allowed us to explore how multiple CDs interact concurrently with such a large macromolecular complex. We thus performed CD-NS simulations with five CDs simultaneously, initially randomly distributed in the solvent. Consistent with previous simulations of small systems, CD^+^ and CD^0^ interacted with the terminal base pairs of DNA chains (Figures 6D and 7C). The DNA chain termini also fluctuated more than the rest of the DNA chain (Figures S49–S54). However, compared with NS in the cellular chromatin environment, the termini in our model are loose and not part of a continuous chromatin chain. Therefore, we will focus here on other parts of the NS and biologically relevant interaction modes.

**Figure 6:**
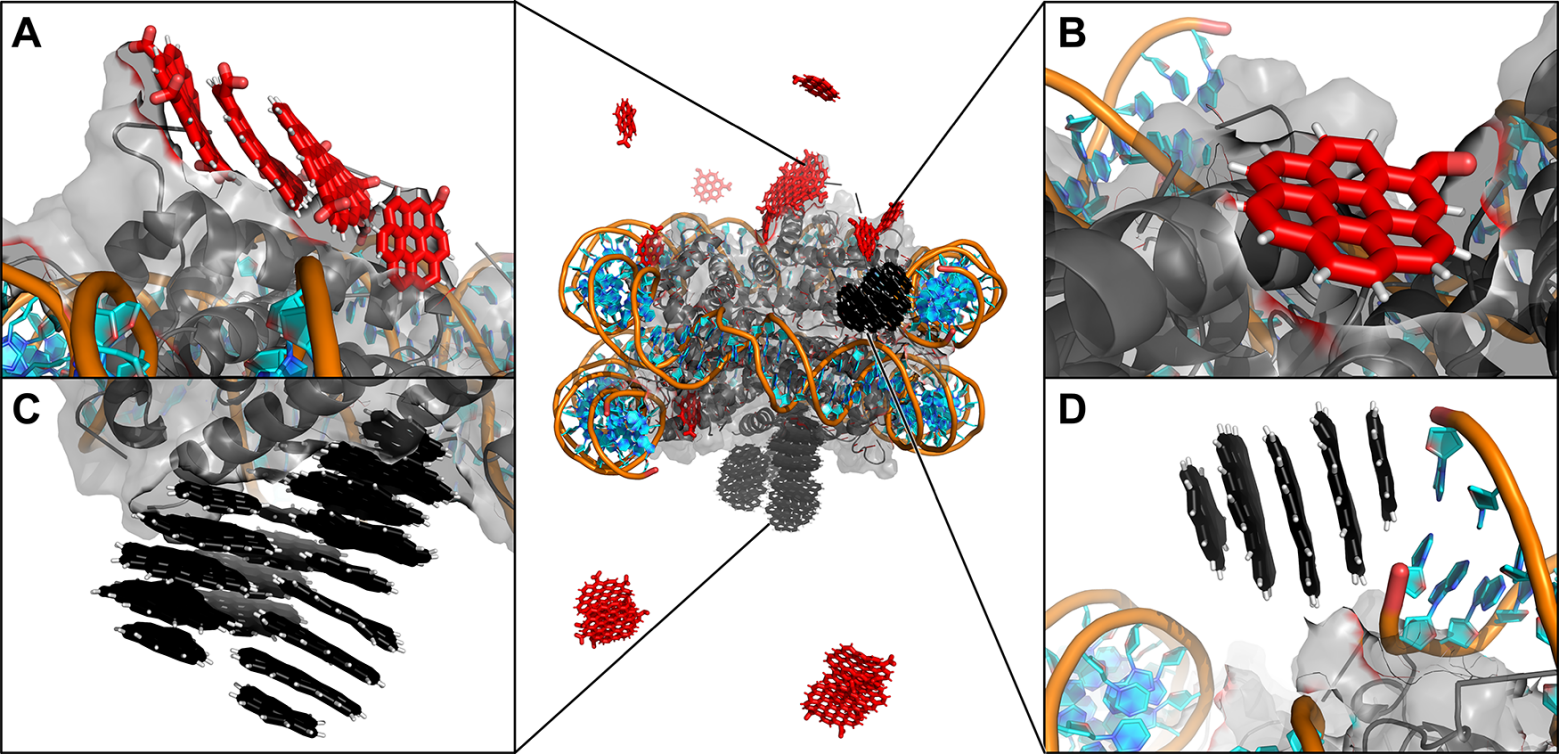
CD^–^ and CD^0^ interact with NS. The final distribution of CDs of both types is superimposed on a single NS structure. The insets show the details of individual interactions: (A) CD^–^ interacting with histones, (B) detached CD^–^ layer interacting with histones, (C) CD^0^ cluster interacting with histones, and (D) CD^0^ stacking on terminal bases. CD^–^ and CD^0^ are shown in red and black sticks, respectively, DNA in cyan/orange cartoon, and histones in gray cartoon/surface.

**Figure 7:**
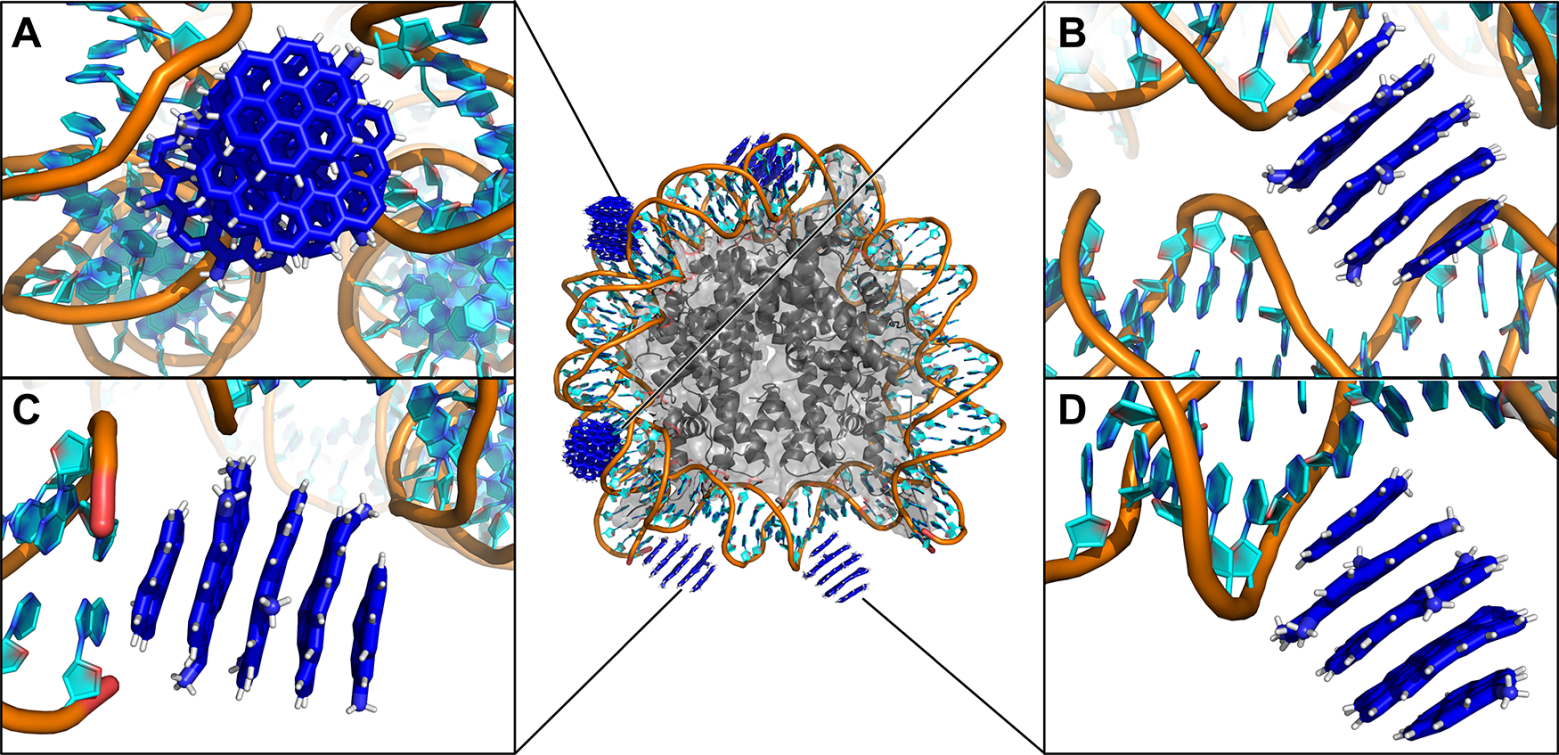
CD^+^ interacts with NS. (A) CD^+^ bound in the minor grooves of two DNA helices, (B) CD^+^ bound in the major grooves, (C) CD^+^ stacked on DNA terminal bases, and (D) CD^+^ bound in the major groove of one DNA helix. CDs are shown in blue sticks, DNA in cyan/orange cartoon, and histones in gray cartoon/surface. In the insets, the histones are omitted for clarity.

Both CD^0^ and CD^–^ preferred interactions with histones over DNA (Figure 6). This observation is consistent with previous simulations on simpler NA motifs, where CD^–^ exhibited minimal DNA affinity due to electrostatic repulsion. In the NS simulations, CD^–^ either remained in the solvent or was associated with the histone surface, sometimes releasing its smallest graphitic layers, which either diffused away or adhered to histones (Figure 6). Similarly, CD^0^ preferentially bound to the histone core, with four CD^0^ ions forming a stable aggregate on the histone surface (Figure 6C). This aggregation enabled by the presence of multiple CDs suggests that in crowded environments, neutral CDs tend to self-associate and/or bind other biomolecules rather than polyanionic NA surface. Due to minimal interaction with DNA, the nucleosomal DNA structure remained largely unaffected in these simulations.

In contrast, positively charged CD^+^ interacted extensively with DNA (Figure 7), consistent with their known nuclear localization.^27^ While one CD^+^ interacted with the DNA terminal base pair (Figure 7C), the others predominantly bound within the minor grooves (Figure 7A) or major grooves (Figure 7D). In four cases, CD^+^ localized between the two DNA gyres and formed hydrogen bonds with DNA backbone (Figure 7A,B). In one case, CD^+^ penetrated deeply between the DNA gyres (Figure S55). Changes in CD^+^ concentration did not play a significant role in the interaction modes, except for the possibility of CD^+^ aggregation (Figure S55).

The CD size strongly influenced the CD-NS interaction strongly. The larger CD7^+^ diffused more slowly in the solvent and, likely due to stronger electrostatic attraction, bound tightly to the DNA chains and usually stayed at the position of the initial contact. Overall, the interaction modes resembled those observed with smaller CD^+^, resulting in CD7^+^ interacting preferentially with major or minor grooves (Figure S54 and Table S6). Unlike in the case of small CD^+^, we did not observe any stacking of CD7^+^ on the terminal base pair. Only when CD7^+^ bound near the terminal DNA bases, it imposed greater stress to NS than the small CD^+^, and in some cases induced partial DNA unwrapping from histone (Figure S41). This effect is probably exaggerated by the absence of a continuous chromatin fiber in our model.

Notably, in any of our simulations, no matter the CD functionalization, amount, or size, we did not observe intercalation of CDs or their graphitic flakes into the DNA, differentiating them from many other DNA-interacting toxic compounds.

#### Effect of CDs on NS

To evaluate the potential structural impact of CDs on the NS, we performed a comprehensive analysis of DNA conformational parameters in systems containing CDs with varying charge. These analyses included both local and global helical parameters to capture possible DNA deformations.

Across all systems, the nucleosomal DNA maintained its canonical wrapping around the histone core, with no substantial deviations observed in any of the helical or groove parameters compared to control simulation without CDs (Figures S38–S41). This consistency held regardless of CD charge and was supported by the average values of the helical parameters. Localized fluctuations were slightly decreased in CD^+^ interaction sites (Figure S49), and no systematic trend indicating structural destabilization was detected. The robustness of the nucleosomal structure in the presence of CDs likely stems from the intrinsic architectural constraints imposed by the histone octamer. Extensive histone-DNA contacts strongly stabilize the DNA conformation (Figures S49–S54), limiting the ability of external agents, such as CDs to induce significant structural changes. This contrasts with smaller NA motifs, where CDs more readily induced modest shifts in helical parameters.

Small effect of CD attachment was observed in systems with positively charged CD^+^, where a subtle localized change in the buckle parameter was detected at a DNA site bound by CD (Figures S38–S41). This effect remained confined to a single base pair step and did not propagate or result in global destabilization. The presence of CD^+^ or CD7^+^ affected locally the distance between the DNA gyres, without a preferred trend – leading to its narrowing or widening; still, the change was only minor (Figure S55). Observing just tiny local changes supports the conclusion that CD^+^ binding does not perturb the nucleosomal DNA conformation (Table S10).

Overall, our results demonstrate that nucleosomal DNA is structurally resilient to the presence of CDs. While CDs can form stable contacts with both histones and DNA, these interactions do not translate into significant conformational changes (Figures S43–S55). This suggests that any potential biological effects of CDs on chromatin are unlikely to arise from direct structural disruption, but rather from interference with dynamic chromatin processes. For example, CDs bound to NS may hinder the access of DNA- or histone-modifying enzymes, thereby affecting epigenetic regulation, or impede NS unwrapping and remodeling by bridging adjacent DNA gyres. These hypotheses could be tested, for example, by immunoprecipitation assays, or in-cell chromatin imaging (tracking), respectively.

## Conclusions

Our atomistic simulations reveal how surface functionalization and charge govern the interaction of CDs with NAs. Although all three types of CDs used in this study, i.e., negatively charged CD^–^, neutral CD^0^, and positively charged CD^+^, sample transient contacts with DNAs and RNAs in solution, only CD^+^ is predicted to remain stably bound under physiological cellular conditions. Electrostatic repulsion drives CD^–^ away from the polyanionic backbone of NAs, diverting it toward basic protein patches (as illustrated here by the nucleosome simulations) or other cationic cellular components. Neutral hydrophobic CD^0^, not involved in long-range Coulombic interactions, instead tends to self-aggregate or partition into hydrophobic environments rather than anchor to NA surfaces.

When binding occurs, it is highly nonspecific. CD^+^ engages NAs through polar contacts between its protonated amino groups and the negatively charged sugar–phosphate backbone, complemented by stacking between its graphitic surface and exposed, noncanonical or terminal nucleobases. Depending on the particle size and shape, CD^+^ can bind to the major or minor groove or at loop/overhang regions. No sequence selectivity was detected.

Despite occasional local adjustments to helical parameters and grooves width, NA integrity is largely preserved. Across >150 μs of cumulative simulations we observed neither intercalation of aromatic flakes into double helices nor disruption of base pairing, even in topologically constrained motifs such as nucleosomes. The histone core stabilizes nucleosomal DNA, and - except for binding events occurring near the loose DNA terminus (a rather artificial scenario) - CDs bound to either DNA or histone do not provoke global unwrapping.

Consequently, potential adverse biological effects, if any, are expected to stem from function rather than structural perturbations. CD^+^ adsorbed to the nucleosome surface or wedged between adjacent DNA gyres may alter local chromatin dynamics and thereby slow processes that rely on rapid DNA accessibility, including transcription, replication, and repair. Our results therefore pose positively charged CDs as viable nanomaterials to target genomic sites. They also highlight the need to tune surface charge and particle size when engineering CDs for nuclear delivery or theranostic applications.

## Supplementary Material





## Data Availability

The data used to prepare this work, including MD run input files, simulation inputs, and data underlying the figures, are available at Zenodo (10.5281/zenodo.17105692).

## Notes

The data file contains average helical parameters for R14AU simulations (XLSX)
Table S1: List of performed simulations of solvated biomolecules without CD molecules; Table S2: List of performed simulations of solvated CD molecules; Table S3: Overview of biosystem simulations involving RNA and CD molecules; Table S4: Overview of biosystem simulations involving noncanonical molecules with CDs; Table S5: Overview of biosystem simulations involving canonical DNA including NS and CD molecules; Table S6: Overview of biosystem simulations involving canonical NA including NS and CD7^+^ molecules; Table S7: Overview of biosystem simulations involving noncanonical NA and CD7^+^ molecules; Table S8: Histidine protonation and tautomer assignments in the nucleosome MD simulations; Table S9: Average ΔSASA values; Table S10: ΔRMSD values for CD^+^ binding events in NS simulations; Figure S1: Properties of CDs; Figure S2: Final snapshots of noncanonical NAs in simulations without carbon dots; Figure S3: Illustration of the final interaction modes between CDs and canonical nucleic acid structures; Figure S4: Interaction of CDs in RNA minor grooves with ribose planes; Figure S5: Heatmaps showing the time evolution of the propeller parameter; Figure S6: Detail (from Figure S5) of the CD^+^ effect on the propeller parameter of the duplex structure; Figure S7: Heatmaps showing the time evolution of the shift parameter; Figure S8: Heatmaps showing the time evolution of the buckle parameter; Figure S9: Structures of CDs interacting with the terminal bases; Figure S10: Atom density maps with respect to the terminal bases (A–C) and on the CD surface, showing the rotation of layers (D–F); Figure S11: Detachment of CD^–^ outer layer; Figure S12: Final interaction modes of R10CG with carbon dots and reference structure; Figure S13: Final interaction modes of R14AU with carbon dots and reference structure; Figure S14: Final interaction modes of R19N with carbon dots and reference structure; Figure S15: Final interaction modes of D12N with carbon dots and reference structure; Figure S16: Interaction of CD7^+^ with canonical RNA and DNA structures; Figure S17: Effect of RNA anchoring on CD^+^ motion; Figure S18: ΔSASA evolution in R14AU system; Figure S19: ΔSASA evolution in R10CG system; Figure S20: ΔSASA evolution in R19N system; Figure S21: ΔSASA evolution in R20CG system; Figure S22: ΔSASA evolution in D12N system; Figure S23: ΔSASA evolution in D20CG system; Figure S24: ΔSASA evolution in canonical systems with CD7^+^; Figure S25: Final interaction modes of TL with carbon dots; Figure S26: Final interaction modes of hG4 with carbon dots; Figure S27: Final interaction modes of cG4 with carbon dots; Figure S28: Position of CD stacked on a tetrad; Figure S29: Final interaction modes of QDJ with CD^–^ and CD^0^; Figure S30: Final interaction modes of QDJ with CD^+^; Figure S31: Final interaction modes of noncanonical DNAs with CD7^+^; Figure S32: ΔSASA evolution in TL system; Figure S33: ΔSASA evolution in hG4 system; Figure S34: ΔSASA evolution in cG4 system; Figure S35: ΔSASA evolution in QDJ system with CD^+^; Figure S36: ΔSASA evolution in QDJ system with CD^–^ and CD^0^; Figure S37: ΔSASA evolution in noncanonical system with CD7^+^; Figures S38–S41: Final structures of NS with bound CD with DNA residues colored by buckle parameter; Figure S42: Structure of NS with a focus on histone; Figures S43 and S44: Per-residue root-mean-square fluctuations (RMSF) of histone subunits; Figures S45 and S46: Top view of the final structure of histones colored by per-residue RMSF; Figures S47 and S48: Dyad view of the final structure of histones colored by per-residue RMSF; Figures S49 and S50: Per-residue root-mean-square fluctuations (RMSF) of nucleosomal DNA; Figures S51 and S52: Top view of the final structure of DNA colored by per-residue RMSF; Figures S53 and S54: Opposite the dyad view of NS with DNA colored by per-residue RMSF; Figure S55: Opposite the dyad view of NS with DNA colored by distance between DNA gyre centers; Figure S56: ΔSASA evolution in NS with five CDs; Figure S57: ΔSASA evolution in NS with varying number and size of CD^+^ (PDF)
