# Supplementary material for: Interaction of Carbon Dots with Nucleic Acids Is Driven by Their Surface Charge
Source: J Chem Inf Model. 2025 Dec 19;66(1):591–604. doi: 10.1021/acs.jcim.5c02242 (PMC12801309; doi:10.1021/acs.jcim.5c02242)
Supplement: Supplementary file 2 [file ci5c02242_si_002.pdf]

Supporting Information
for

Interaction of Carbon Dots with Nucleic Acids Is Driven by Their Surface Charge

Andrea Nedělníková[1,2,3,4], Petr Stadlbauer[2,5], Pavel Banáš[1], Jiří Šponer[1,5], Michal Otyepka[1,3], Petra Kührová[1*] and Markéta Paloncýová[1*]

[1] Regional Center of Advanced Technologies and Materials, Czech Advanced Technology and Research Institute (CATRIN), Palacký University Olomouc, Šlechtitelů 27, 779 00 Olomouc, Czech Republic
[2] Department of Physical Chemistry, Faculty of Science, Palacký University Olomouc, 17. listopadu 12, 771 46 Olomouc, Czech Republic
[3] IT4Innovations, VŠB – Technical University of Ostrava, 17. listopadu 2172/15, 708 00 Ostrava-Poruba, Czech Republic
[4] Faculty of Electrical Engineering and Computer Science, VSB – Technical University of Ostrava, 17. listopadu 2172/15, 708 00 Ostrava-Poruba, Czech Republic
[5] Institute of Biophysics of the Czech Academy of Sciences, Královopolská 135, 612 00 Brno, Czech Republic

* corresponding authors, e-mails: petra.kuhrova@upol.cz, marketa.paloncyova@upol.cz

Table of Content

Supporting Tables.....5

Analyzed properties in Supporting Tables: **5**

Table S1: List of performed simulations of solvated biomolecules without CD molecules.....5

Table S2: List of performed simulations of solvated CD molecules 5

Table S3: Overview of biosystem simulations involving RNA and CD molecules.....6

Table S4: Overview of biosystem simulations involving non-canonical molecules with CDs.7

Table S5: Overview of biosystem simulations involving canonical DNA including NS and CD molecules.....8

Table S6: Overview of biosystem simulations involving canonical NA including NS and $CD7^+$ molecules. 9

Table S7: Overview of biosystem simulations involving non-canonical NA and $CD7^+$ molecules. 9

Table S8: Histidine protonation and tautomer assignments in the nucleosome MD simulations. 10

Table S9: Average ΔSASA values.....10

Event-aligned RMSD analysis of CD^+ binding to nucleosomal DNA ***10***

Table S10: ΔRMSD values for CD^+ binding events in NS simulations.....11

Carbon dots properties ***12***

Figure S1: Properties of CDs. 12

Reference simulations of nucleic acids ***13***

Figure S2: Final snapshots of non-canonical NAs in simulations without carbon dots. 13

Interaction modes between CDs and canonical NA helices 14

Figure S3: Illustration of the final interaction modes between CDs and canonical nucleic acid structures. 14

Figure S4: Interaction of CDs in RNA minor grooves with ribose planes 15

Effect of CD^+ on the structure of R20CG and D20CG ***16***

Figure S5: Heatmaps showing the time evolution of the propeller parameter 16

Figure S6: Detail (from Figure S5) of the CD+ effect on the propeller parameter of the duplex structure.....16

Figure S7: Heatmaps showing the time evolution of the shift parameter.....17

Figure S8: Heatmaps showing the time evolution of the buckle parameter 17

Figure S9: Structures of CDs interacting with the terminal bases 18

Figure S10: Atom density maps with respect to the terminal bases (A-C) and on the CD surface, showing the rotation of layers (D-F). 18

Figure S11: Detachment of CD^- outer layer.....19

Figure S12: Final interaction modes of **R10CG** with carbon dots and reference structure. ..19

Figure S13: Final interaction modes of **R14AU** with carbon dots and reference structure....20

Figure S14: Final interaction modes of **R19N** with carbon dots and reference structure.......21

Figure S15: Final interaction modes of **D12N** with carbon dots and reference structure.......22
Figure S16: Interaction of $CD7^+$ with canonical RNA and DNA structures..........................23
Figure S17: Effect of RNA anchoring on CD^+ motion. 24
Figure S18: ΔSASA evolution in **R14AU** system. 25
Figure S19: ΔSASA evolution in **R10CG** system. 26
Figure S20: ΔSASA evolution in **R19N** system. 27
Figure S21: ΔSASA evolution in **R20CG** system. 28
Figure S22: ΔSASA evolution in **D12N** system. 29
Figure S23: ΔSASA evolution in **D20CG** system. 30
Figure S24: ΔSASA evolution in canonical systems with $CD7^+$. 31
Interaction modes between CD and non-canonical NA structures 32
Figure S25: Final interaction modes of **TL** with carbon dots...32
Figure S26: Final interaction modes of **hG4** with carbon dots..33
Figure S27: Final interaction modes of **cG4** with carbon dots. 34
Figure S28: Position of CD stacked on a tetrad...35
Figure S29: Final interaction modes of **QDJ** with CD^- and CD^0. 36
Figure S30: Final interaction modes of **QDJ** with CD^+. 37
Figure S31: Final interaction modes of non-canonical DNAs with $CD7^+$.............................38
Figure S32: ΔSASA evolution in **TL** system...39
Figure S33: ΔSASA evolution in **hG4** system. 40
Figure S34: ΔSASA evolution in **cG4** system...41
Figure S35: ΔSASA evolution in **QDJ** system with CD^+...42
Figure S36: ΔSASA evolution in **QDJ** system with CD^- and CD^0.......................................43
Figure S37: ΔSASA evolution in non-canonical systems with CD7+..................................44
Nucleosome 45
Figure S38: Final structures of NS with bound CD...45
Figure S39: Final structures of NS with bound CD...46
Figure S40: Final structures of NS with bound CD...47
Figure S41: Final structures of NS with bound CD...48
Figure S42: Structure of NS with a focus on histone. 49
Figure S43: Per-residue root mean square fluctuations (RMSF) of histone subunits.............50
Figure S44: Per-residue root mean square fluctuations (RMSF) of histone subunits.............51
Figure S45: Top view of the final structure of histones colored by per-residue RMSF 52
Figure S46: Top view of the final structure of histones colored by per-residue RMSF 53
Figure S47: Dyad view of the final structure of histones colored by per-residue RMSF.......54
Figure S48: Dyad view of the final structure of histones colored by per-residue RMSF.......55
Figure S49: Per-residue root mean square fluctuations (RMSF) of nucleosomal DNA.........56

Figure S50: Per-residue root mean square fluctuations (RMSF) of nucleosomal DNA.........57
Figure S51: Top view of the final structure of DNA colored by per-residue RMSF..............58
Figure S52: Top view of the final structure of DNA colored by per-residue RMSF..............59
Figure S53: Opposite the dyad view of NS with DNA colored by per-residue RMSF 60
Figure S54: Opposite the dyad view of NS with DNA colored by per-residue RMSF 61
Figure S55: Opposite the dyad view of NS with DNA colored by distance between DNA gyre centers 62
Figure S56: ΔSASA evolution in **NS** with five CDs. 63
Figure S57: ΔSASA evolution in **NS** with varying number and size of CD^+. 64

Supporting Tables

Analyzed properties in Supporting Tables:
BT – binding time, i.e., the time when the number of nonnative contacts between CD and NA exceeded 100
BM – resulting binding mode, see main text Figures 2 and 4
HB – average number of hydrogen bonds between the biomolecule and CD calculated from the last 200 ns of simulation
CD global elongation – see Figure S1 and Eq. S1 for definition. Elongation is not given for CDs that have disintegrated. The presented number was calculated from the last 200 ns of simulation

Table S1: *List of performed simulations of solvated biomolecules without CD molecules*

Type	System	Box size (nm)	Length (μs)	Ions	
				K^+	Cl^-
RNA	Free R14AU	5.2 × 5.5 × 5.8	1	36	10
	Free R10CG	4.1 × 4.3 × 5.6	1	27	9
	Free R19N	4.2 × 4.6 × 7.6	1	39	13
	Free TL	4.6 × 4.9 × 5.2	1	18	11
DNA	Free D12N	4.4 × 4.6 × 6.4	1	43	21
	Free cG4	4.9 × 5.2 × 6.4	1	30	13
	Free hG4	5.9 × 6.6 × 7.5	1	44	26
	Free QDJ	7.1 × 6.1 × 6.3	1	38	19
	Free NS	15 × 15 × 15	0.4	495	289

Table S2: *List of performed simulations of solvated CD molecules*

Type	System	Box size (nm)	Length (μs)	Ions		CD global elongation
				Na^+	Cl^-	
CD	Free CD^-	5.3 × 5.6 × 6.0	1	27	16	disintegrated
	Free CD^+	5.5 × 5.6 × 5.6	1	16	25	1.02
	Free CD^0	5.3 × 5.5 × 5.6	1	15	15	1.02
	Free $CD7^+$	6.7 × 6.6 × 6.2	1	25	51	not calculated

Table S3: *Overview of biosystem simulations involving RNA and CD molecules. The Results section includes the following resulting binding modes: 0 – No interaction; Ia – Minor groove; Ib – Major groove, II – Base(s) (see main text Figure 2).*

System		#sim	Simulation settings: Box size (nm)	Ions: K^+	Ions: Cl^-	Length (µs)	Results: BT (ns)	BM	HB	CD global elongation
R14AU	CD^-	1	7	67	30	1	79	II	3	1.36
		2	9	103	66	1	651	II	2	1.24
		3	9	103	66	1	844	II	3	1.22
		4	9	103	66	1	-	0	0	1.22
	CD^+	1	7	47	30	1	45	II	1	1.02
		2	9	83	66	1	277	II	1	1.02
		3	9	83	66	1	51	Ia	2	1.02
		4	9	83	66	1	176	II+Ib	4	1.02
	CD^0	1	7	56	30	1	80	II	0	1.02
		2	9	92	66	1	260	Ia	0	1.00
		3	9	66	66	1	2	II	0	1.02
		4	9	92	66	1	232	Ia	0	1.02
R10CG	CD^-	1	7	59	30	1	157	II	2	1.26
		2	8	75	46	1	884	II	2	1.20
		3	8	75	46	1	303	Ia	2	disintegrated
		4	8	75	46	1	541	II	2	1.53
	CD^+	1	7	39	30	1	222	II+Ib	3	1.02
		2	8	55	46	1	324	II	1	1.02
		3	8	55	46	1	49	Ib	8	1.02
		4	8	55	46	1	111	II+Ib	2	1.01
	CD^0	1	7	48	30	1	224	Ia	0	1.02
		2	8	64	46	1	315	Ia	0	1.02
		3	8	64	46	1	61	II	0	1.02
		4	8	64	46	1	56	Ia	0	1.02
R19N	CD^-	1	10	137	90	1	-	0	0	1.21
		2	10	137	90	1	554	II	2	1.25
		3	10	137	90	1	40	II	2	2.20
		4	10	137	90	1	332	Ia	2	1.31
	CD^+	1	10	117	90	1	199	II	1	1.02
		2	10	117	90	1	338	II	1	1.02
		3	10	117	90	1	38	Ib	6	1.01
		4	10	117	90	1	46	Ia	2	1.03
	CD^0	1	10	127	91	1	238	Ia	0	1.02
		2	10	127	91	1	-	0	0	1.02
		3	10	127	91	1	2	II	0	1.02
		4	10	127	91	1	762	II	0	disintegrated
R20CG	CD^+	1	10	119	90	1	2	Ib	6	1.02
		2	10	119	90	1	15	Ia	1	1.02
		3	10	119	90	1	5	Ib	3	1.02
		4	10	119	90	1	4	Ib	5	1.01
		5	10	119	90	1	40	Ib	5	1.03
		6	10	119	90	1	4	II	0	1.02
		7	10	119	90	1	10	Ia	1	1.03
		8	10	119	90	1	13	Ia	1	1.02
		9	10	119	90	1	4	II	1	1.02
		10	10	119	90	1	48	Ib	5	1.01

Table S4: *Overview of biosystem simulations involving non-canonical molecules with CDs. The Results section includes the following resulting binding modes: 0 – No interaction, I – Sugar-phosphate backbone, IIa – Unpaired bases, IIb – Terminal bases, IIc – Tetrad bases (see main text Figure 4).*

System		#sim	Simulation settings				Results			
			Box size (nm)	Ions		Length (µs)	BT (ns)	BM	HB	CD global elongation
				K^+	Cl^-					
TL	CD^-	1	7	49	31	1	-	0	0	1.23
		2	7	49	31	1	559	IIa	1	1.22
		3	7	49	31	1	31	IIa	2	1.27
		4	7	49	31	1	744	IIb	2	1.21
	CD^+	1	7	29	31	1	163	I	4	1.01
		2	7	29	31	1.2	55	I	3	1.02
		3	7	29	31	1	377	I	1	1.02
		4	7	29	31	1.2	0	I	1	1.02
	CD^0	1	7	38	31	1	133	IIa	0	1.02
		2	7	38	31	1	48	I	0	1.03
		3	7	38	31	1	445	IIa	0	1.02
		4	7	38	31	1.2	179	IIa	0	1.02
hG4	CD^-	1	8	79	46	1	857	IIa	1	1.23
		2	8	79	46	1	94	IIc	-	1.22
		3	8	79	46	1	58	IIa	3	1.26
		4	8	79	46	1	80	IIa	2	1.81
	CD^+	1	8	59	46	1	72	IIc	5	1.02
		2	8	59	46	1	113	I	4	1.02
		3	8	59	46	1	52	IIa	4	1.01
		4	8	59	46	1	493	IIa	4	1.02
	CD^0	1	8	68	46	1	305	I	0	1.02
		2	8	68	46	1	333	I	0	1.02
		3	8	68	46	1	196	I	0	1.03
		4	8	68	46	1	857	I	0	1.02
cG4	CD^-	1	8	78	46	1.5	4	IIc+ IIa	0	disintegrated
		2	8	78	46	1.5	374	IIa	0	1.23
		3	8	78	46	1.5	143	IIa	0	1.23
		4	8	78	46	1.5	1045	IIa	0	1.21
	CD^+	1	8	58	46	1.5	5	IIa	3	1.02
		2	8	58	46	1.5	9	IIc	3	1.03
		3	8	58	46	1.5	130	IIa	3	1.02
		4	8	58	46	1.5	217	IIa	3	1.03
	CD^0	1	8	67	46	1.5	7	IIa	0	1.02
		2	8	67	46	1.5	42	IIc	0	1.04
		3	8	67	46	1.9	65	IIa+IIc	0	1.02
		4	8	67	46	1.5	122	IIc+IIa	0	1.43
QDJ	CD^-	1	8	78	46	2	-	0	0	1.22
		2	8	78	46	2.4	743	IIc	0	1.18
		3	8	78	46	2	-	0	0	1.24
		4	8	78	46	2	4	IIc	0	1.25
		5	8	78	46	2	304	IIa	0	1.51
	CD^+	1	8	58	46	2.4	23	I	2	1.01
		2	8	58	46	2.4	32	IIa	1	1.01
		3	8	58	46	2	40	IIa	3	1.03
		4	8	58	46	2	36	IIc	1	1.04
		5	8	58	46	2.4	31	IIa	1	1.02
		6	8	58	46	2.4	17	I	2	1.02

	7	8	58	46	2	68	I	1	1.02
	8	8	58	46	2	2	IIa	3	1.01
	9	8	58	46	2	2	IIa	2	1.02
	10	8	58	46	2.4	24	IIa	3	1.01
CD^0	1	8	67	46	2	6	IIc	0	1.02
	2	8	67	46	2	249	IIa	0	1.02
	3	8	67	46	2	345	IIa	0	1.02
	4	8	67	46	2	54	IIa	0	1.05
	5	8	67	46	2	33	IIc	0	1.04

Table S5: *Overview of biosystem simulations involving canonical DNA including NS and CD molecules. The Results section includes the following resulting binding modes:0 – No interaction; Ia – Minor groove; Ib – Major groove, II – Base(s) (see main text Figure 2).*

System		#sim	Simulation settings				Results			
			Box size (nm)	Ions		Length (µs)	BT (ns)	BM	HB	CD global elongation
				K^+	Cl^-					
D12N	CD^-	1	8	79	46	1	857	II	1	1.23
		2	8	79	46	1	94	0	-	1.22
		3	8	79	46	1	58	II	3	1.26
		4	8	79	46	1	80	II	2	1.81
	CD^+	1	8	59	46	1	72	Ia	5	1.02
		2	8	59	46	1	113	Ia	4	1.02
		3	8	59	46	1	52	Ib	4	1.01
		4	8	59	46	1	493	Ib	4	1.02
	CD^0	1	8	68	46	1	305	II	0	1.02
		2	8	68	46	1	333	II	0	1.02
		3	8	68	46	1	196	Ia	0	1.03
		4	8	68	46	1	857	II	0	1.02
D20CG	CD^+	1	10	119	90	0.4	20	Ia	3	1.02
		2	10	119	90	0.4	16	Ia	2	1.01
		3	10	119	90	0.4	7	Ib	2	1.01
		4	10	119	90	0.4	3	Ib	3	1.01
		5	10	119	90	0.4	31	Ib	2	1.01
		6	10	119	90	0.4	9	Ia	3	1.01
		7	10	119	90	0.4	11	Ia	4	1.01
		8	10	119	90	0.4	37	Ib	2	1.01
		9	10	119	90	0.4	22	Ia	2	1.01
		10	10	119	90	0.4	15	Ia	3	1.01
NS*	CD^-	1	15	549	288	1	No interaction (4x), Histone (1x)			
		2	15	549	288	0.5	No interaction (3x), Histone (2x)			
	CD^+	1	15	449	288	1	Interaction in groove/s (4x), Terminal bases (1x)			
		2	15	449	288	0.4	Interaction in groove/s (4x), Terminal bases (1x)			
	$3CD^+$	1	15	467	288	0.5	Minor groove (2x), Major grooves (1x)			
	$8CD^+$	1	15	421	287	0.5	Minor groove (3x), Major groove/s (4x), Stacking on CD (1x)			
	CD^0	1	15	494	288	1	Terminal bases (1x), Histone (4x)			

**NS systems with $3CD^+$ and $8CD^+$ describe systems with three and eight CD^+, respectively. All other NS systems contain five CDs.*

Table S6: *Overview of biosystem simulations involving canonical NA including NS and CD7^{+} molecules. The Results section includes the following resulting binding modes: Ia – Minor groove; Ib – Major groove, II – Base(s) (see main text Figure 2).*

System		#sim	Simulation settings				Results
			Box size (nm)	Ions		Length (µs)	BM
				K^{+}	Cl^{-}		
**R14AU**	**CD7^{+}**	1	9	65	65	1	II,Ib
		2	9	65	65	0.8	Ia
		3	9	65	65	0.8	Ib
**R19N**	**CD7^{+}**	1	10	100	90	1	Ib
		2	11	130	120	1	Ib
**D12N**	**CD7^{+}**	1	8	61	65	0.9	Ia
		2	8	61	65	0.8	Ia
		3	8	61	65	0.8	Ia
**NS**	**CD7^{+}**	1	15	360	284	0.5	Minor groove (2x), Major groove/s (3x)
		2	15	361	285	0.5	Minor groove (5x)
		3	16.4	206	0	0.5	Minor groove (1x), Major groove/s (3x)

Table S7: *Overview of biosystem simulations involving non-canonical NA and CD7^{+} molecules. The Results section includes the following resulting binding modes: 0 – No interaction, I – Sugar-phosphate backbone, IIa – Unpaired bases, IIb – Terminal bases, IIc – Tetrad bases (see main text Figure 4).*

System		#sim	Simulation settings				Results
			Box size (nm)	Ions		Length (µs)	BM
				K^{+}	Cl^{-}		
**hG4**	**CD7^{+}**	1	10	84	90	1.1	I
		2	10	84	90	1.2	I+IIa
		3	10	84	90	1.1	I
**cG4**	**CD7^{+}**	1	10	85	90	1.1	I
		2	10	85	90	1.2	IIa
		3	10	85	90	1.1	I
**QDJ**	**CD7^{+}**	1	10	85	90	0.6	I+IIa
		2	10	85	90	0.8	I
		3	10	85	90	1	I

Table S8: Histidine protonation and tautomer assignments in the nucleosome MD simulations. Residue numbering follows the simulated PDB; AMBER residue names are used.

ResID	Residue name	Tautomer	Protonation
2	HIP	n/a (diprotonated (ND1-H & NE2-H)	+1
76	HID	δ-tautomer (ND1-H)	0
148	HIE	ε-tautomer (NE2-H)	0
196	HIE	ε-tautomer (NE2-H)	0
247	HID	δ-tautomer (ND1-H)	0
303	HIP	n/a (diprotonated (ND1-H & NE2-H)	+1
336	HIP	n/a (diprotonated (ND1-H & NE2-H)	+1
363	HIP	n/a (diprotonated (ND1-H & NE2-H)	+1
382	HIP	n/a (diprotonated (ND1-H & NE2-H)	+1
456	HID	δ-tautomer (ND1-H)	0
535	HIE	ε-tautomer (NE2-H)	0
579	HIE	ε-tautomer (NE2-H)	0
630	HID	δ-tautomer (ND1-H)	0
683	HIP	n/a (diprotonated (ND1-H & NE2-H)	+1
716	HIP	n/a (diprotonated (ND1-H & NE2-H)	+1
743	HIP	n/a (diprotonated (ND1-H & NE2-H)	+1

Table S9: *Average ΔSASA values (in Å^2). The ΔSASA results for individual binding modes are averaged over the last 200 ns of simulations over all types of CD as evaluated in Tables S3-S5 except for D20CG and R20CG. The simulations resulting in combination of multiple binding modes were excluded. ΔSASA time evolution graphs of individual systems are shown in Figures S18-S24 and S32-S37.*

	Canonical structures		Non-canonical structures	
**Binding mode**	**RNA**	**DNA**	**RNA**	**DNA**
I			-4.78±1.28	-4.50±1.75
Ia	-5.61±0.84	-6.49±1.90		
Ib	-7.51±1.80	-6.40±1.19		
II	-4.46±1.38	-4.25±1.19		
IIa			-3.18±0.98	-5.62±1.81
IIb			-3.79±0.39	
IIc				-7.91±2.20

Event-aligned RMSD analysis of CD$^+$ binding to nucleosomal DNA

To quantify local structural changes in DNA upon CD$^+$ binding, we computed per-residue differences in RMSD (ΔRMSD) aligned to individual binding events. All RMSD values were computed for each DNA residue relative to the initial structure.

Definition of binding events:
A residue was considered to be involved in a binding event if the number of non-native contacts (NNC) with a CD$^+$ particle increased from zero to NNC ≥ 1, marking the first contact frame.

For each event, we extracted RMSD values for the involved residue from five frames before and after the binding event (i.e., a ±5-frame window, corresponding to ±0.5 ns). ΔRMSD was calculated as the difference between the average RMSD in the post-event and pre-event

windows. For each system, we report the number of affected residues (n), median ΔRMSD, 95% confidence interval, and one-sided sign-test p-value.

Table S10: *ΔRMSD values for CD^+ binding events in NS simulations. Residue-level median ΔRMSD values were computed in a ±5-frame (i.e., 1 ns total) window around CD^+ binding events. Each frame corresponds to 100 ps. The table summarizes the number of residues (n), median ΔRMSD, 95% confidence intervals (CI), and one-sided sign test p-values. Here, n indicates the number of residues that experienced at least one binding event, defined by a contact threshold (non-native contacts).*

System		n (residues)	Median ΔRMSD [Å]	95% CI [Å]	Sign-test p-value
CD^+	1	122	-0.019	[-0.032, -0.003]	0.0232
	2	94	0.032	[0.000, 0.070]	0.0747
$3CD^+$	1	46	0.031	[-0.009, 0.164]	0.1893
$8CD^+$	1	98	0.127	[0.077, 0.191]	0.0020
$CD7^+$	1	64	0.075	[0.013, 0.152]	0.0169
	2	40	0.094	[-0.007, 0.226]	0.2682
	3	80	0.071	[-0.041, 0.174]	0.4340

Carbon dots properties

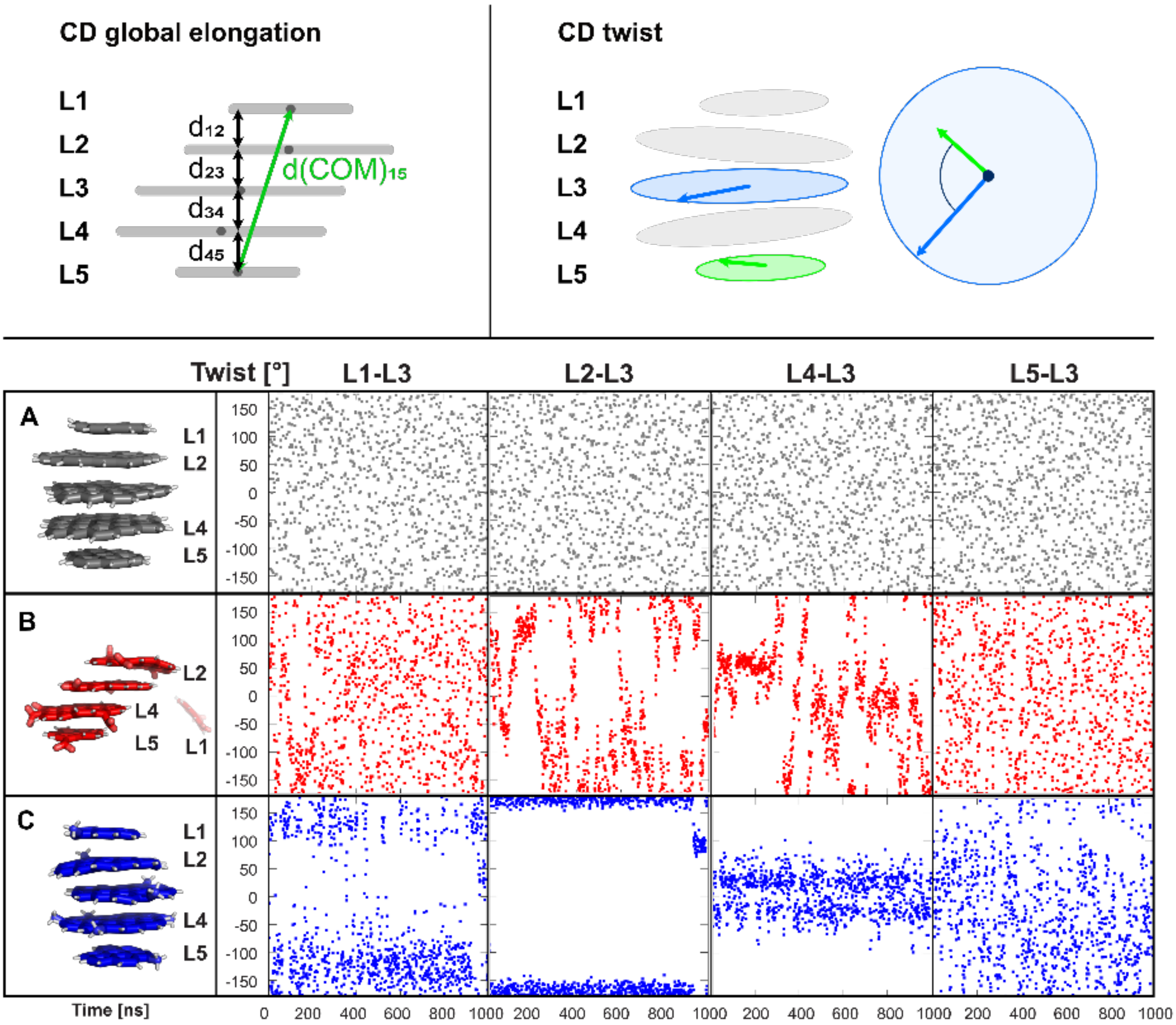

***Figure S1**: Properties of CDs.*

Top left: Definition of CD global elongation as used in Tables S2-S5, calculated as the ratio of the distances between the centers of mass (COMs) of the two opposite outermost layers and the sum of the distances between adjacent layers along the z-axis (rise) (Eq. S1):

$$elongation = \frac{d(COM)_{1|n}}{dz_{1|2} + dz_{2|3} + \cdots + dz_{m|n}} \qquad \text{Eq. S1}$$

For example, the value '1' means no horizontal slide.

Top right: Definition of twist between CD layers, measuring relative orientation between individual CD layers. The vector of a layer is defined as the direction from the layer COM and to one of the "corners" of the layer hexagon. The presented twist is calculated as the angle between the reference layer (middle layer) vector and the monitored layer vector projected on the reference layer plane.

Bottom panel: Evolution of the twist of individual CD layers in selected simulations: A) CD^0 preserved spherical shape and free rotation of all layers; B) CD^- outermost layers were transiently detached; C) CD^+ preserved a spherical shape and reduced rotation to avoid electrostatic interactions between the charged functional groups.

Reference simulations of nucleic acids

For overview of all performed simulations see Table S1. In all only-canonical structures simulations (without CDs) we observed a fraying of at least one base pair. The most significant effect on the NA structure was observed with R10CG, where 5' ends stacked on each other (Figure S12). Longer R14AU (Figure S13) terminal bases frayed as well, with temporary interactions of the frayed terminal base in the minor groove. The structure relaxed back afterwards, therefore for the reference properties evaluation we used the non-fraying simulations parts.

In the simulations of non-canonical structures we did not observe any fraying (Figure S2). The ions embedded in the channels of hG4, cG4 and QDJ originating from the crystal structures stayed in their positions without exchange with other ions in solvent. In cG4 we observed stacking of the overhand bases on the tetrad.

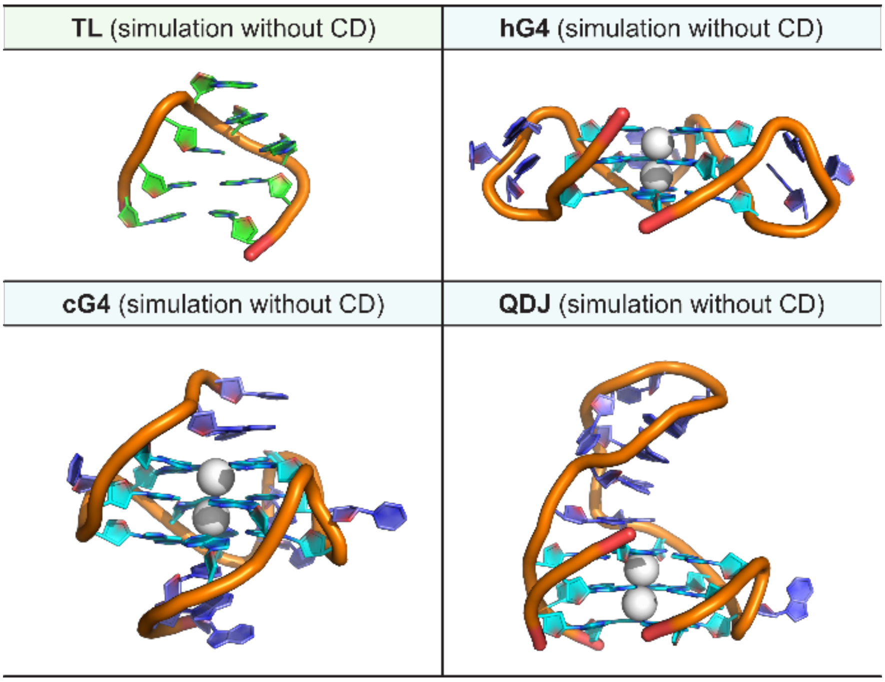

Figure S2: *Final snapshots of non-canonical NAs in simulations without carbon dots.*

Interaction modes between CDs and canonical NA helices

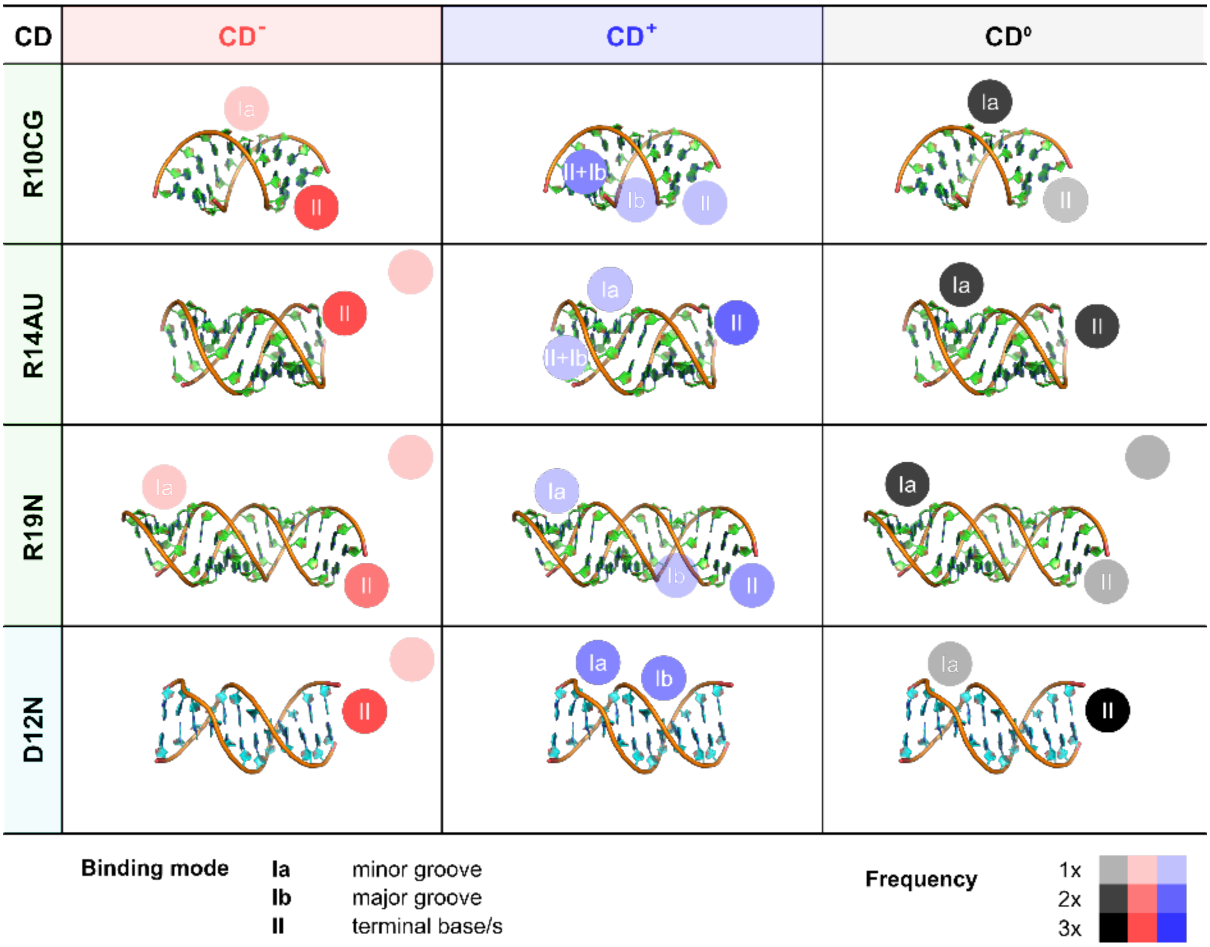

Figure S3: *Illustration of the final interaction modes between CDs and canonical nucleic acid structures. The labels indicate the interaction mode and representative binding position, while color intensity reflects the frequency of observations.*

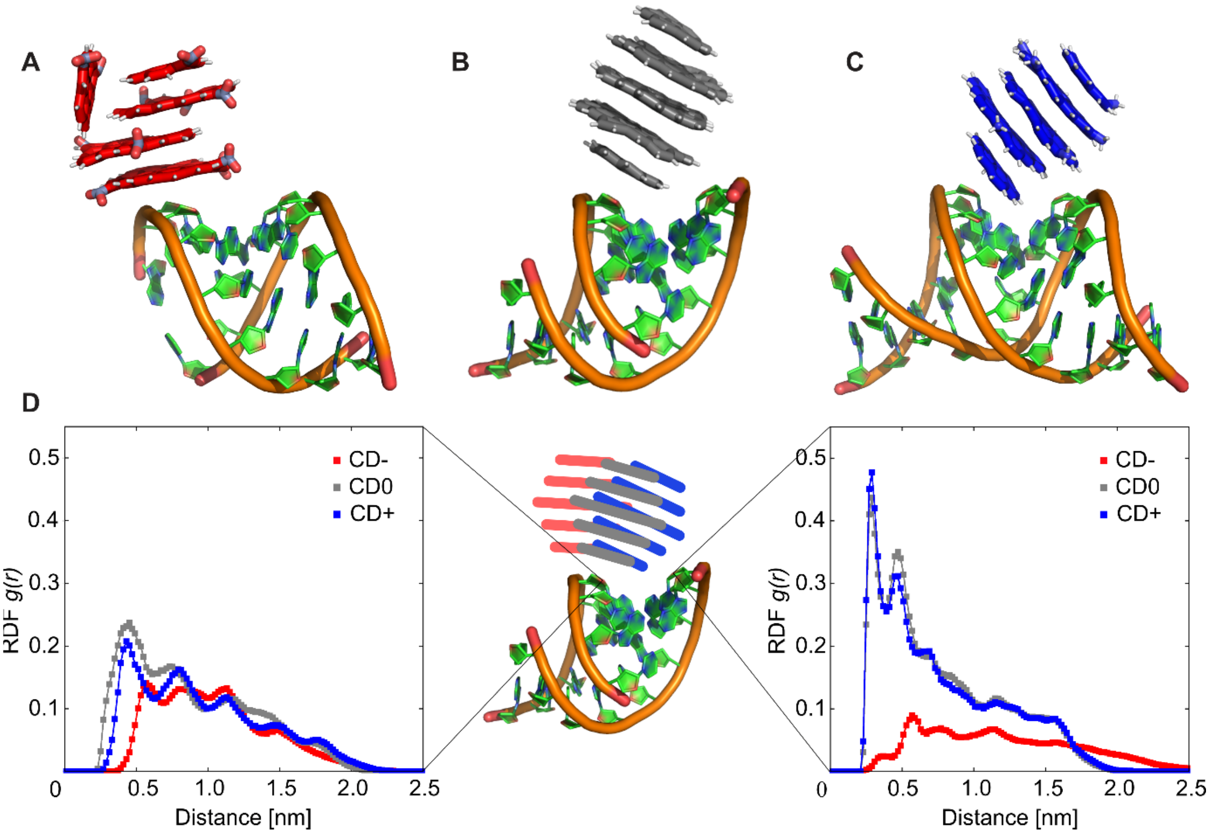

Figure S4: *Interaction of CDs in RNA minor grooves with ribose planes: A) R10CG; B) R10CG; C) R14AU. Graphs of radial distribution functions (RDF) between CD atoms and the displayed attached-ribose oxygen (left, lines indicate the mentioned oxygen) or between CD atoms and ribose oxygen at the other strand of the helix (right).*

Effect of CD^+ on the structure of R20CG and D20CG

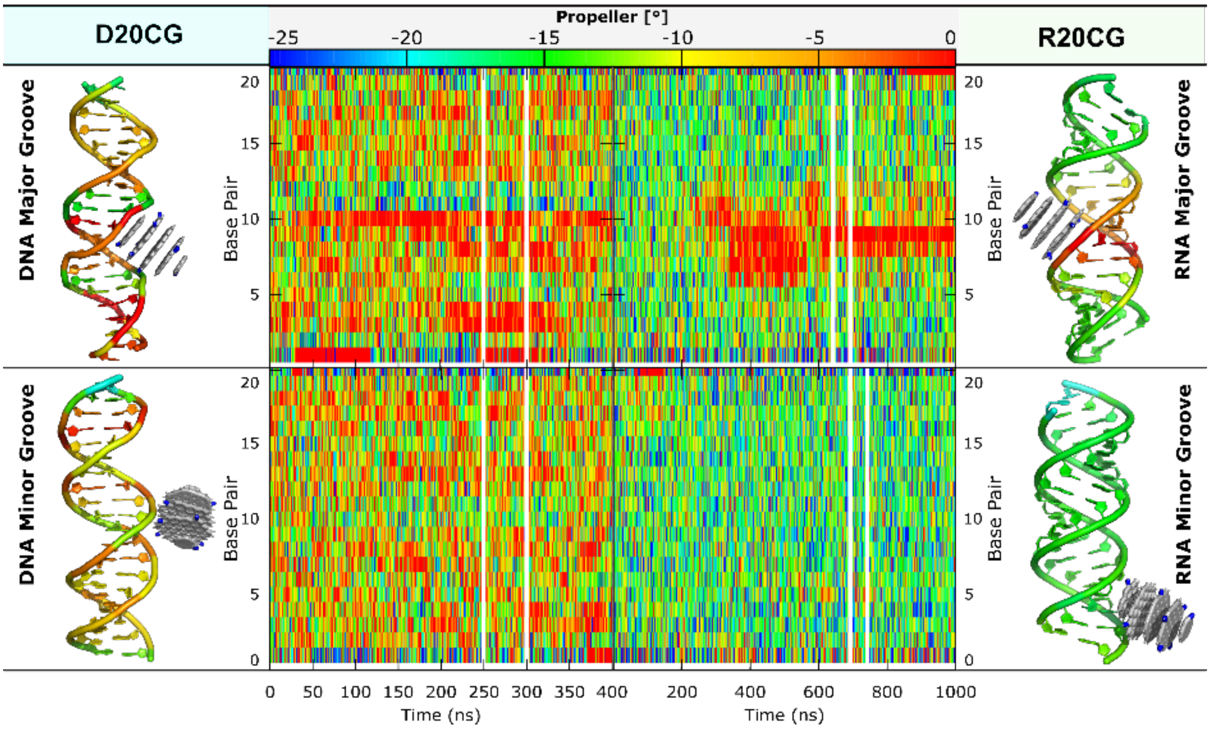

Figure S5: *Heatmaps showing the time evolution of the propeller parameter for DNA (left) and RNA (right) duplexes with a CD bound in the major (top) or minor (bottom) groove. Colors indicate the value of the parameter per base pair over time. Side panels show representative structures with the CD in the corresponding groove colored by the per-residue average of propeller parameter from the highlighted part of the simulation (indicated in white).*

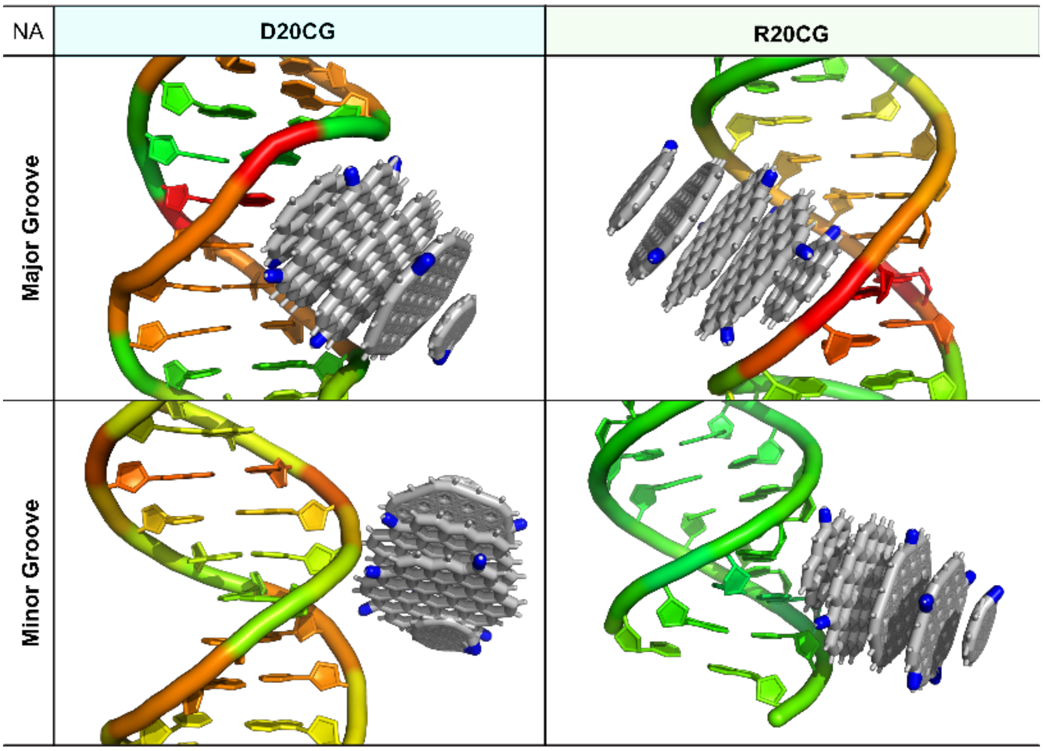

Figure S6: Detail (from Figure S5) of the CD+ effect on the propeller parameter of the duplex structure.

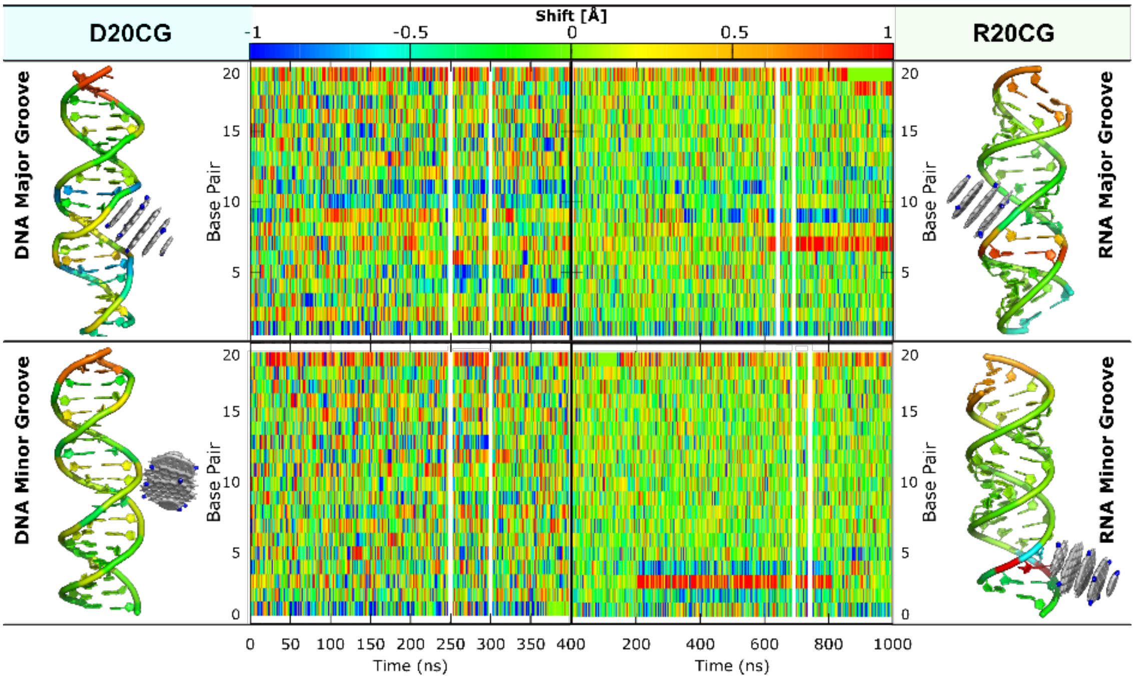

Figure S7: Heatmaps showing the time evolution of the shift parameter for DNA (left) and RNA (right) duplexes with a CD bound in the major (top) or minor (bottom) groove. Colors indicate the value of the parameter per base pair over time. Side panels show representative structures with the CD in the corresponding groove colored by the per-residue average of shift parameter from the highlighted part of the simulation (indicated in white).

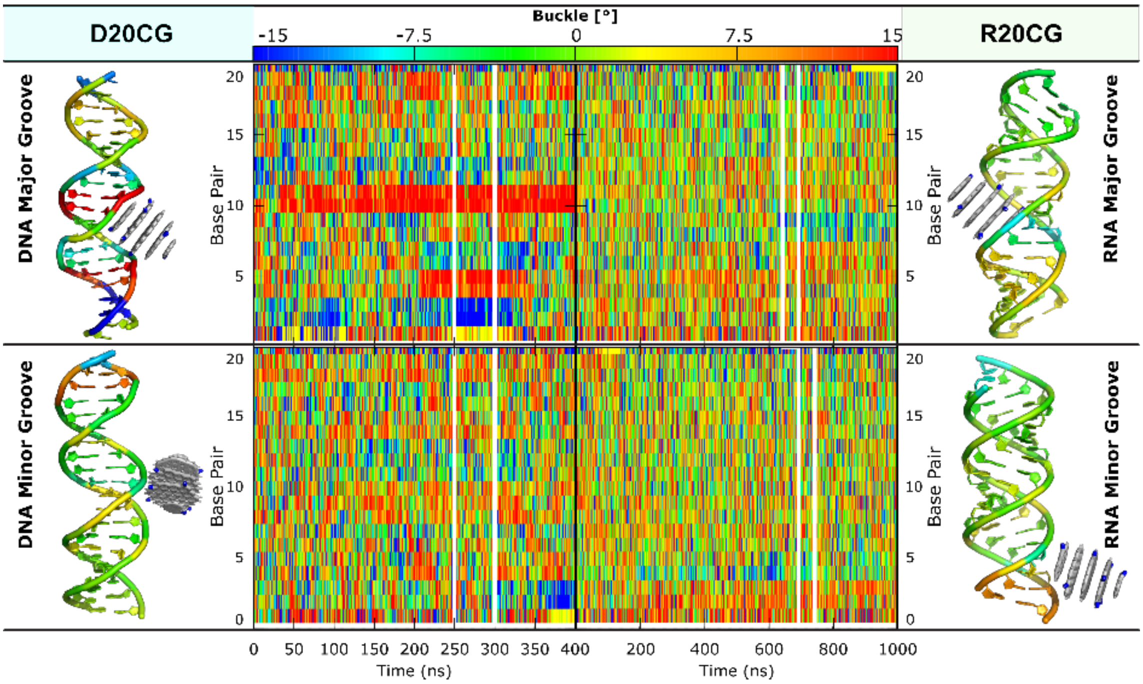

Figure S8: Heatmaps showing the time evolution of the buckle parameter for DNA (left) and RNA (right) duplexes with a CD bound in the major (top) or minor (bottom) groove. Colors indicate the value of the parameter per base pair over time. Side panels show representative structures with the CD in the corresponding groove colored by the per-residue average of buckle parameter from the highlighted part of the simulation (indicated in white).

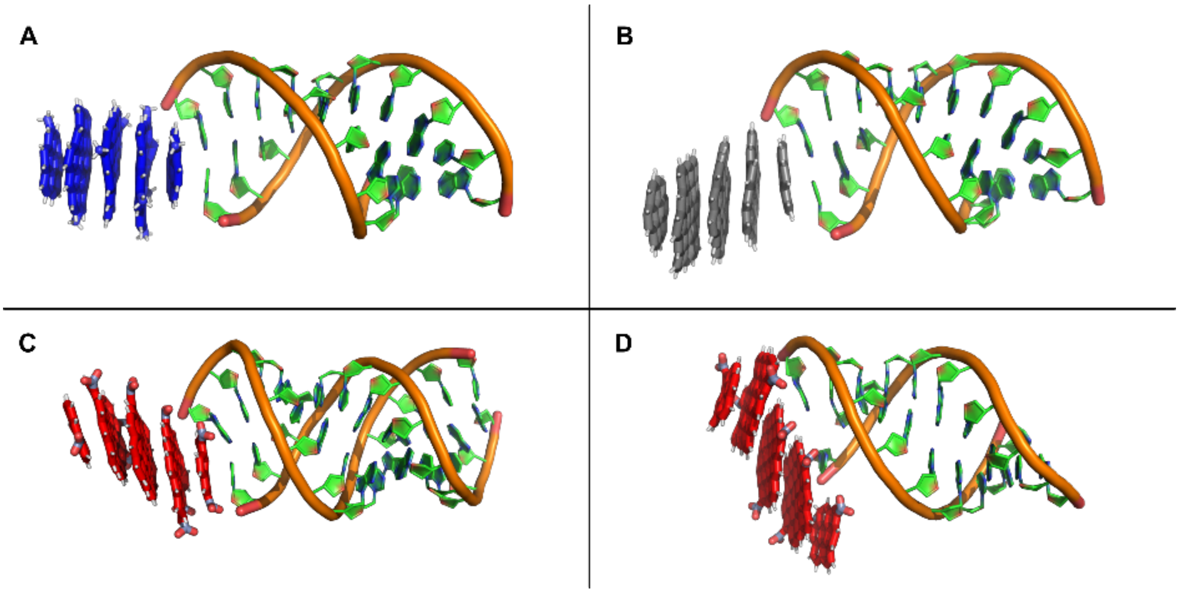

Figure S9: *Structures of CDs interacting with the terminal bases: A) R10CG; B) R10CG; C) R14AU, D) R10CG*

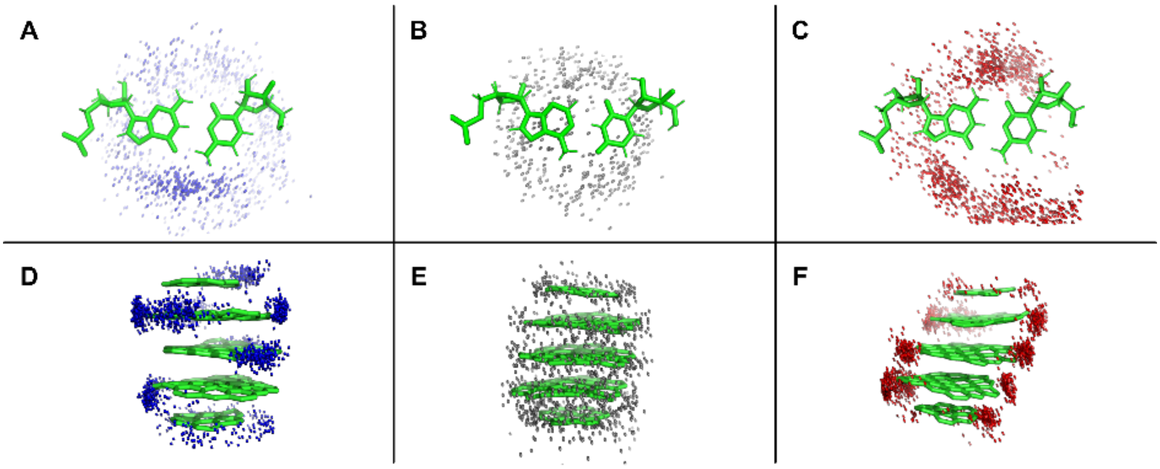

Figure S10: *Atom density maps with respect to the terminal bases (A-C) and on the CD surface, showing the rotation of layers (D-F). Trajectories in panels A-C were fitted to the terminal bases, trajectories in panels D-F were fitted on the whole CD structure. The displayed atoms are nitrogens in ammonium groups (A, D), hydrogens on CD^0 (B, E) and oxygens in carboxylate groups (C,F).*

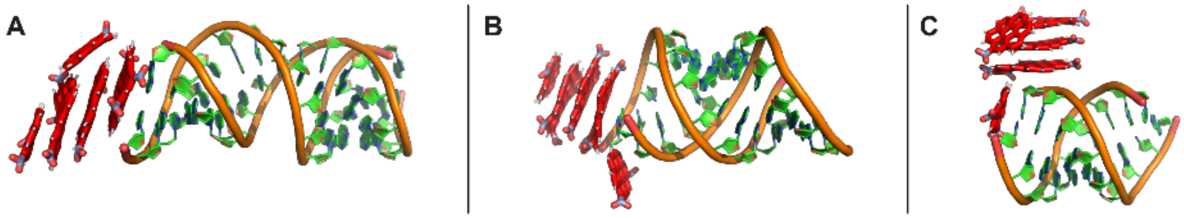

Figure S11: Detachment of CD⁻ outer layer to interact A) with CD⁻ side (R19N), B) with minor groove (R14AU), C) with CD⁻ side and terminal bases (R10CG).

Averaged parameters			
Parameter	(Å)	Parameter	(°)
Shear	-0.20	Opening	2.87
Stagger	0.01	Buckle	-0.90
Stretch	0.04	Propeller	-16.07
X-disp.	-3.77	Inclination	16.18
Y-disp.	0.23	Tip	1.98
Shift	0.07	Tilt	1.93
Slide	-1.68	Roll	10.04
Rise	2.30	Twist	31.10

R10CG (simulation without CD)

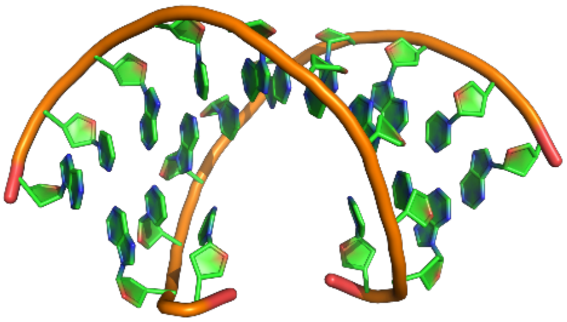

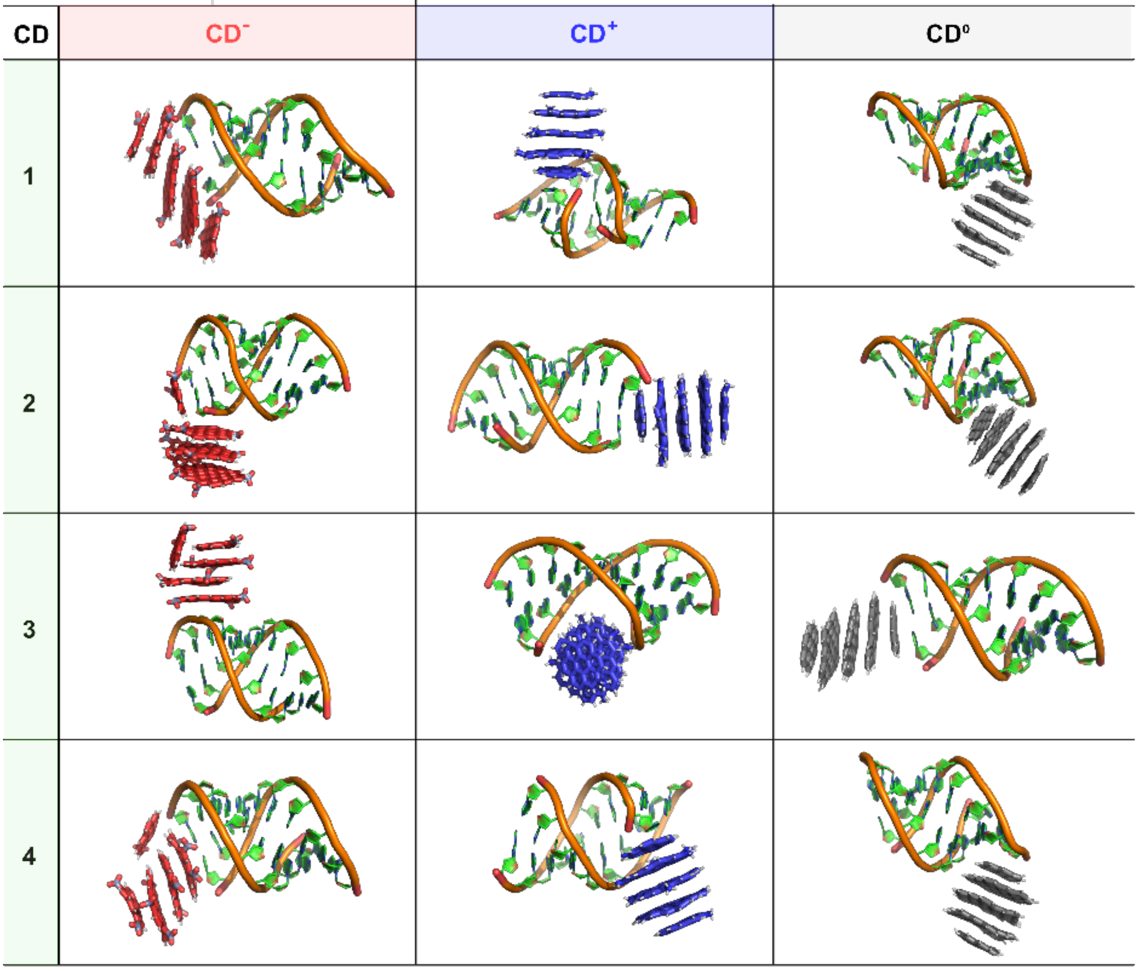

*Figure S12: Final interaction modes of **R10CG** with carbon dots and reference structure.*

Averaged parameters				R14AU (simulation without CD)
Parameter	(Å)	Parameter	(°)	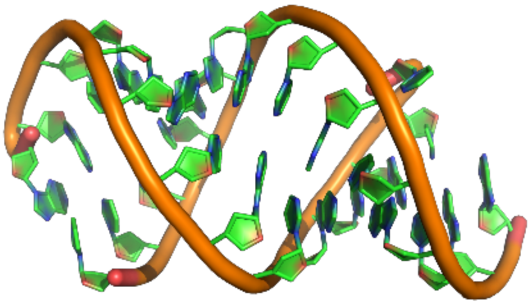
Shear	0.02	Opening	-0.20	
Stagger	0.08	Buckle	-0.03	
Stretch	-0.07	Propeller	-12.26	
X-disp.	-4.32	Inclination	13.89	
Y-disp.	0.00	Tip	0.00	
Shift	0.00	Tilt	0.02	
Slide	-1.73	Roll	8.02	
Rise	2.75	Twist	33.08	

CD	CD$^-$	CD$^+$	CD^0
1	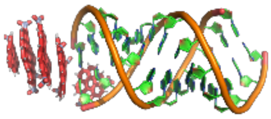	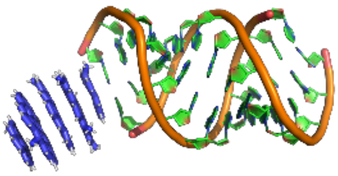	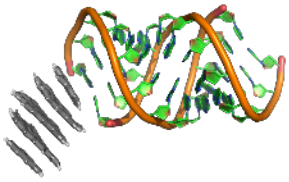
2	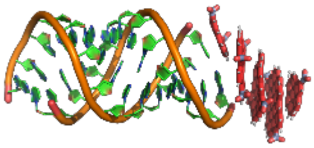	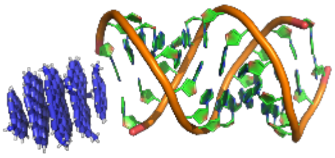	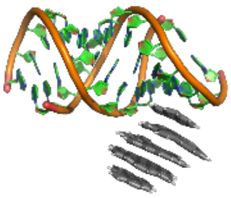
3	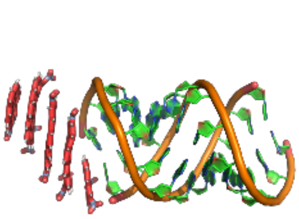	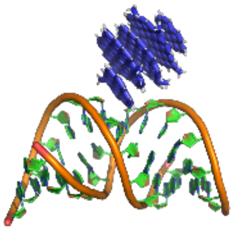	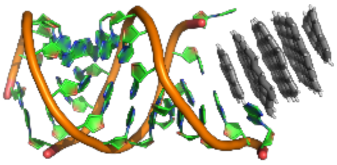
4	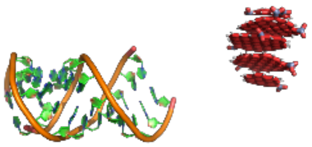	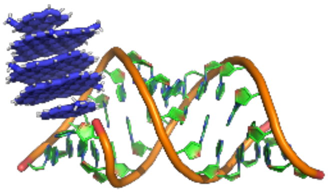	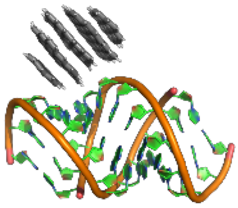

Figure S13: *Final interaction modes of* ***R14AU*** *with carbon dots and reference structure.*

Averaged parameters			
Parameter	(Å)	Parameter	(°)
Shear	-0.12	Opening	-0.87
Stagger	0.23	Buckle	-2.16
Stretch	-0.03	Propeller	-10.02
X-disp.	-4.45	Inclination	14.17
Y-disp.	-0.02	Tip	-0.33
Shift	-0.01	Tilt	0.93
Slide	-1.78	Roll	6.96
Rise	2.51	Twist	32.04

R19N (simulation without CD)

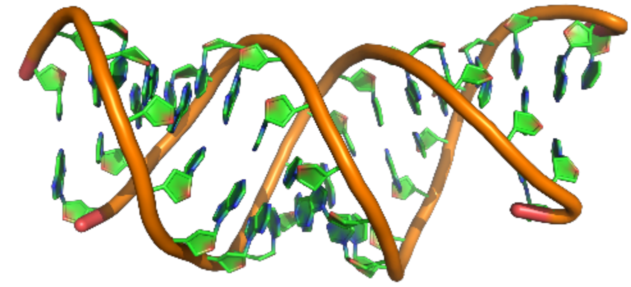

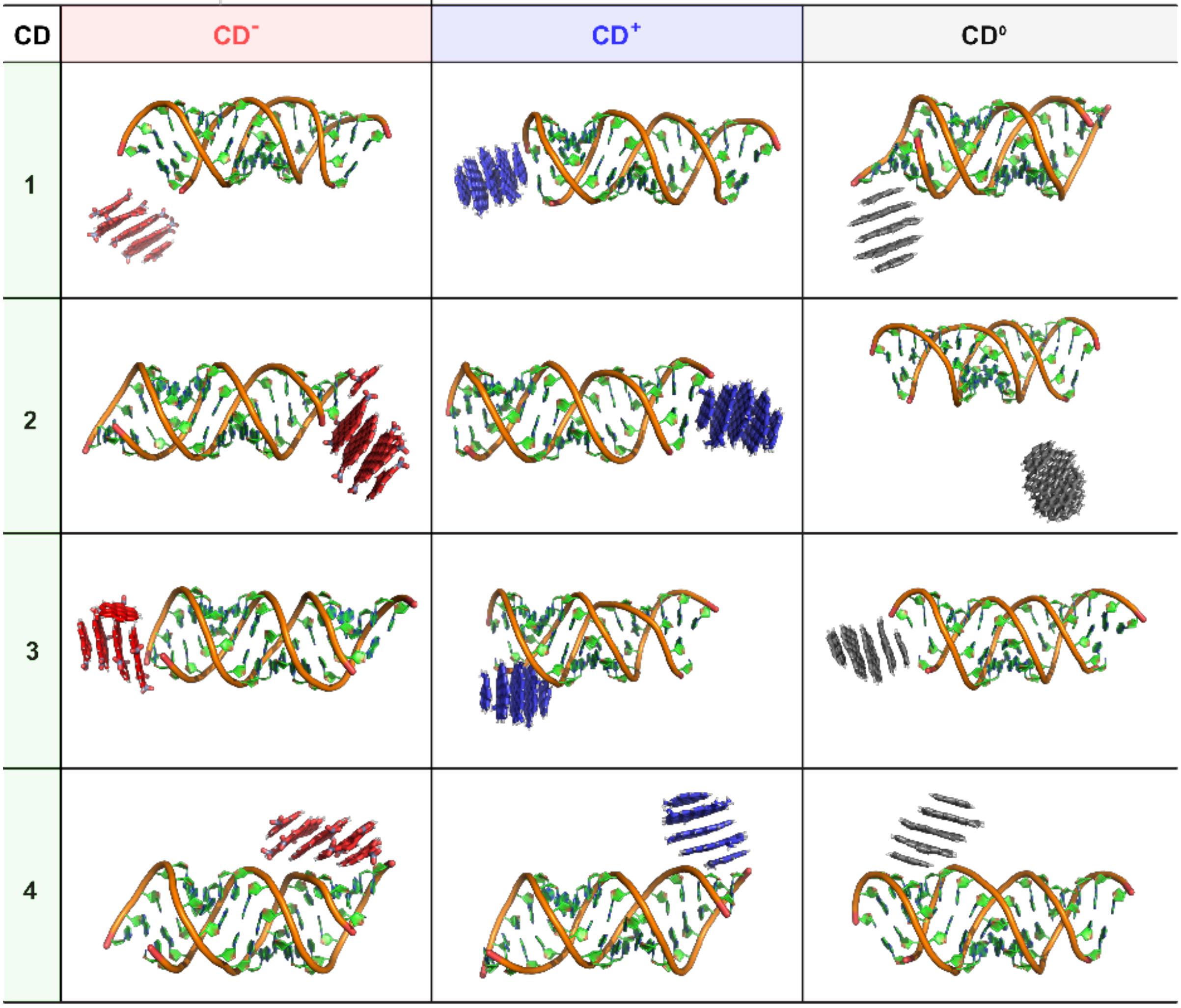

Figure S14: *Final interaction modes of* ***R19N*** *with carbon dots and reference structure.*

Averaged parameters			
Parameter	(Å)	Parameter	(°)
Shear	-0.01	Opening	0.31
Stagger	0.01	Buckle	-0.34
Stretch	0.05	Propeller	-10.38
X-disp.	-0.56	Inclination	3.97
Y-disp.	0.01	Tip	-0.02
Shift	0.00	Tilt	0.04
Slide	-0.06	Roll	2.23
Rise	3.22	Twist	35.43

D12N (simulation without CD)

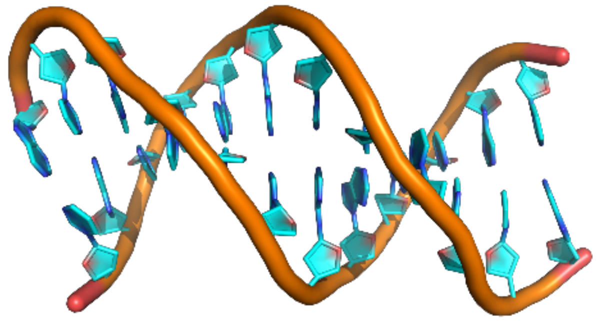

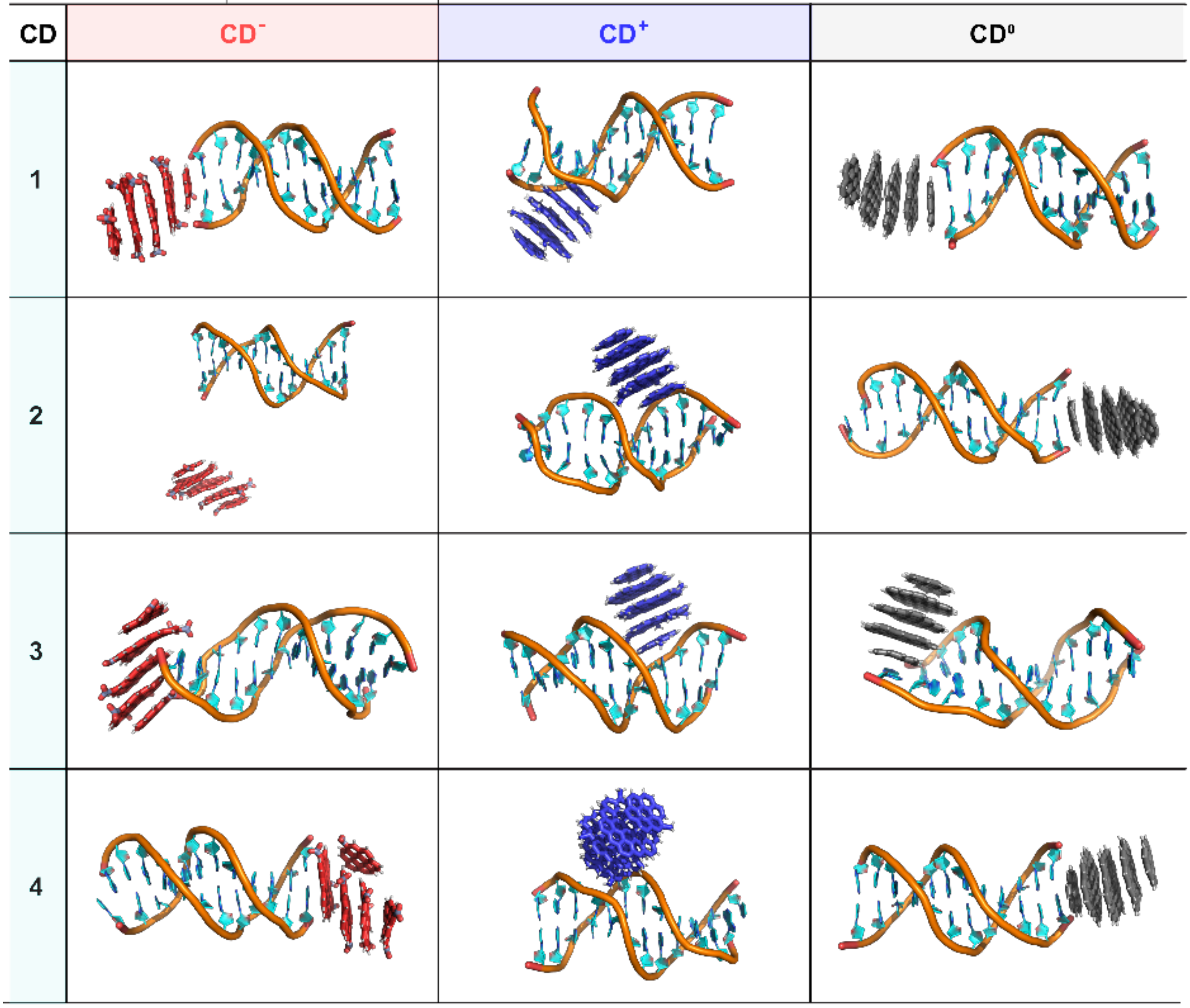

Figure S15: *Final interaction modes of **D12N** with carbon dots and reference structure.*

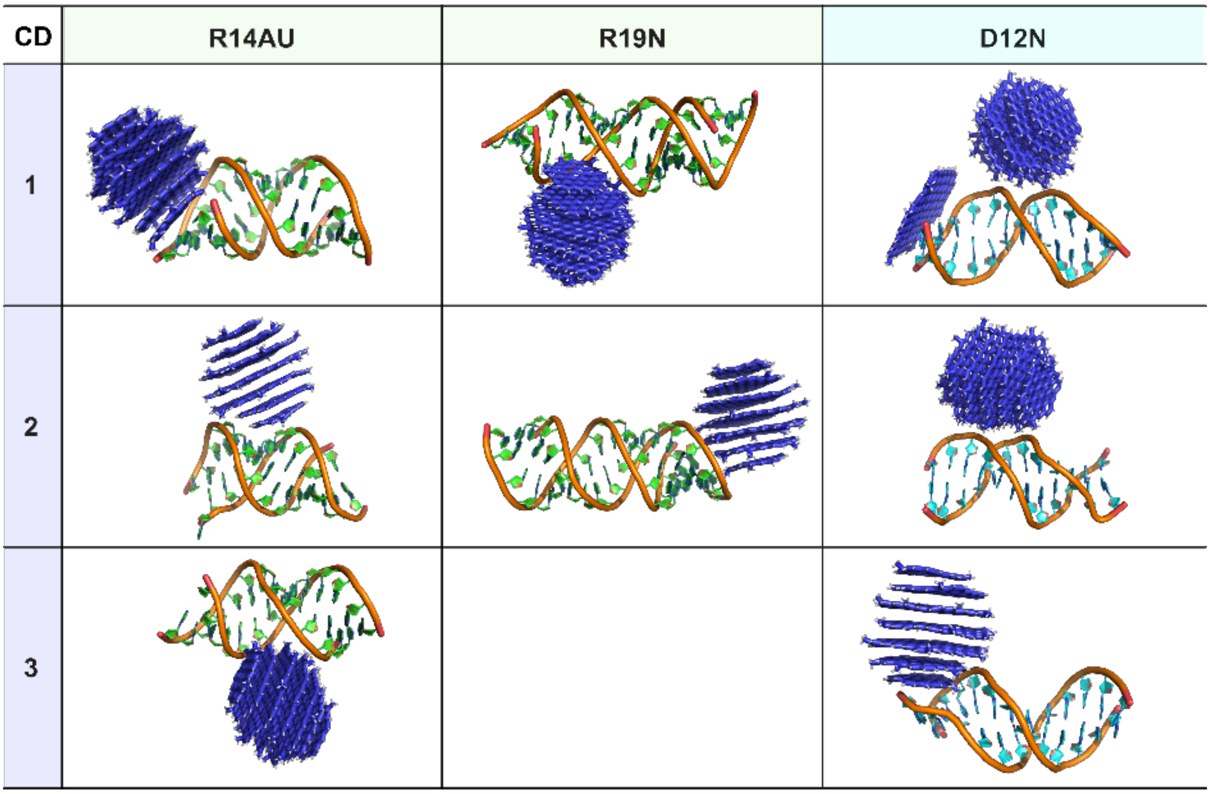

Figure S16: *Interaction of CD7^{+} with canonical RNA and DNA structures. CD7^{+} interacted with ribose and hydrogen bonded to the phosphate of the other backbone with RNA. The interactions of CD7^{+} with terminal bases included also tilting of CD7^{+} to the major groove, hydrogen bonding with the sugar-phosphate backbone. The sole interaction with the RNA major groove was observed, but unlike with smaller CDs, CD7^{+} did not fully embed into the major groove due to its larger radius. With dsDNA, CD7^{+} interacted with DNA minor groove, with CD7^{+} ammonium groups interacting by hydrogen bonds with phosphates on both backbones. The structure of CD7^{+} in D12N_1 simulation disassembled during the equilibration phase (considered as artifact). No interaction with terminal bases or the major groove was observed with DNA.*

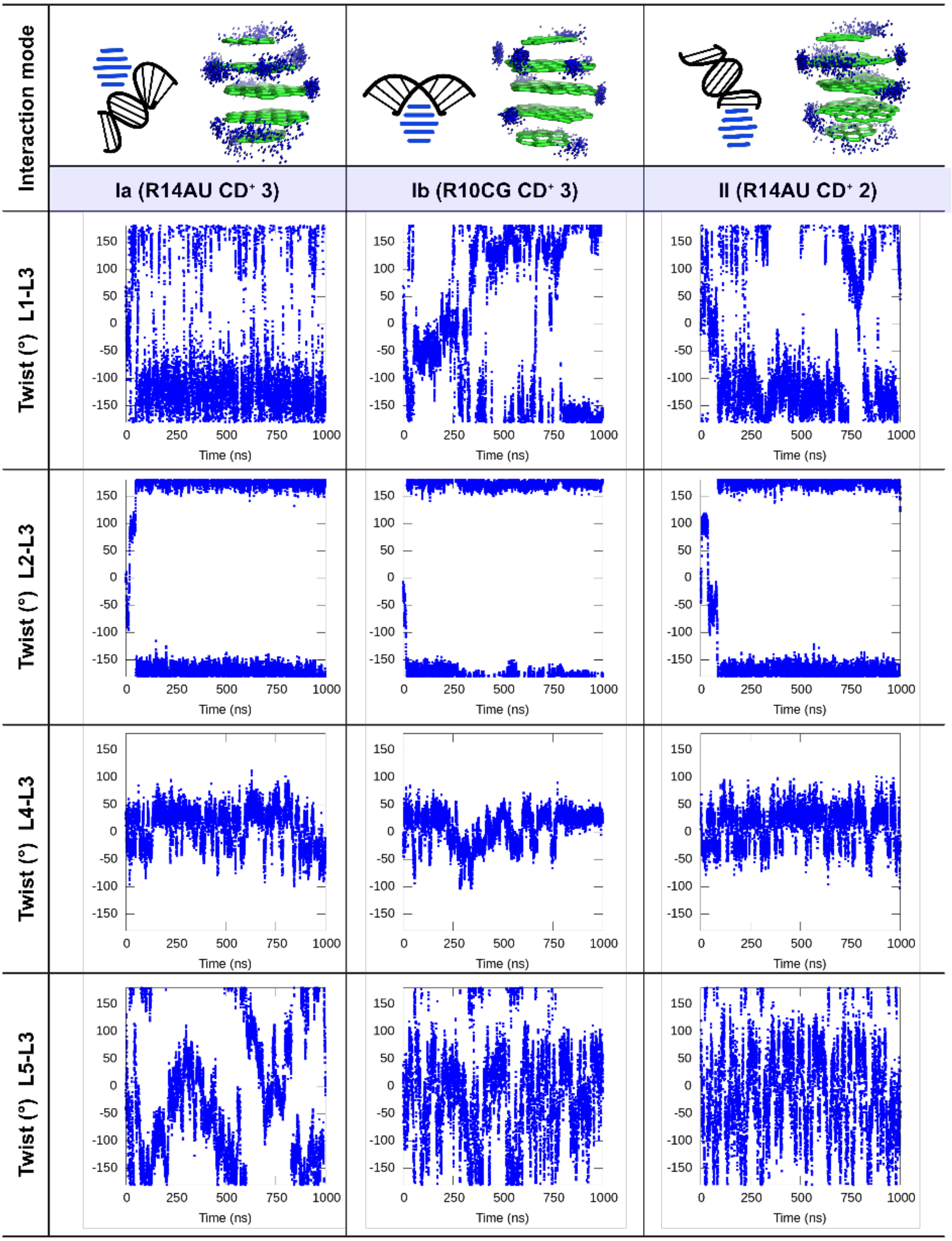

Figure S17: *Effect of RNA anchoring on CD$^+$ motion. Upper panel: Density maps of amonium nitrogens interacting in RNA minor groove (Ia, left), major groove (Ib, middle) and with terminal bases (II, right) and the corresponding evolution of the global twist of individual CD layers in respect to the middle layer (L3) (lower panels). CD$^+$ is anchored after binding to RNA and internal rotation of CD layers is reduced by interaction with the backbone. The density maps were calculated on the trajectories fitted to the whole CD structure.*

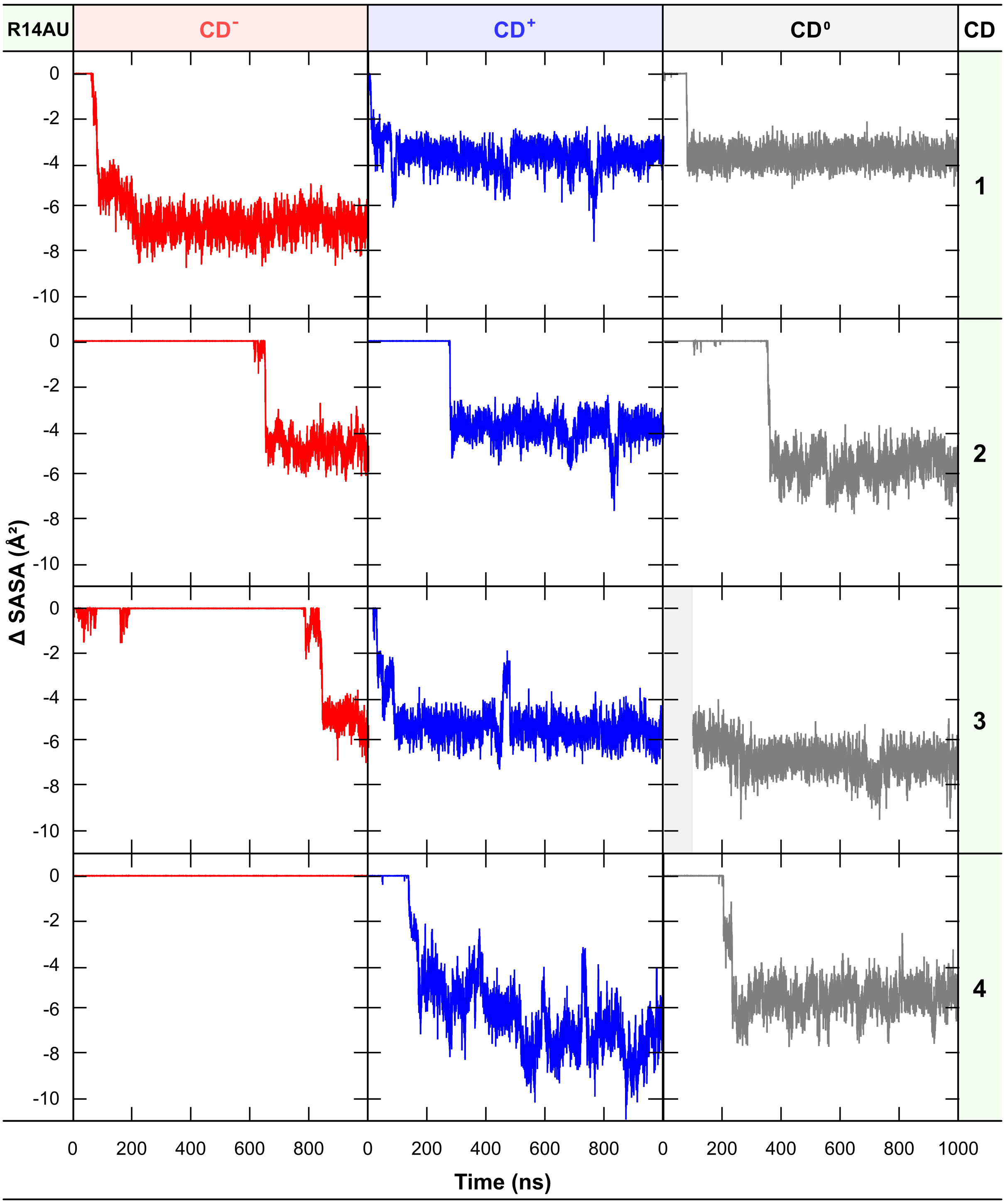

Figure S18: *ΔSASA evolution in* ***R14AU*** *system. ΔSASA corresponds to the difference between SASA calculated for the whole NA+CD complex and the sum of SASA of NA and CD separately. The first 100 ns of replicate 3 of CD^0 simulation is lacking data.*

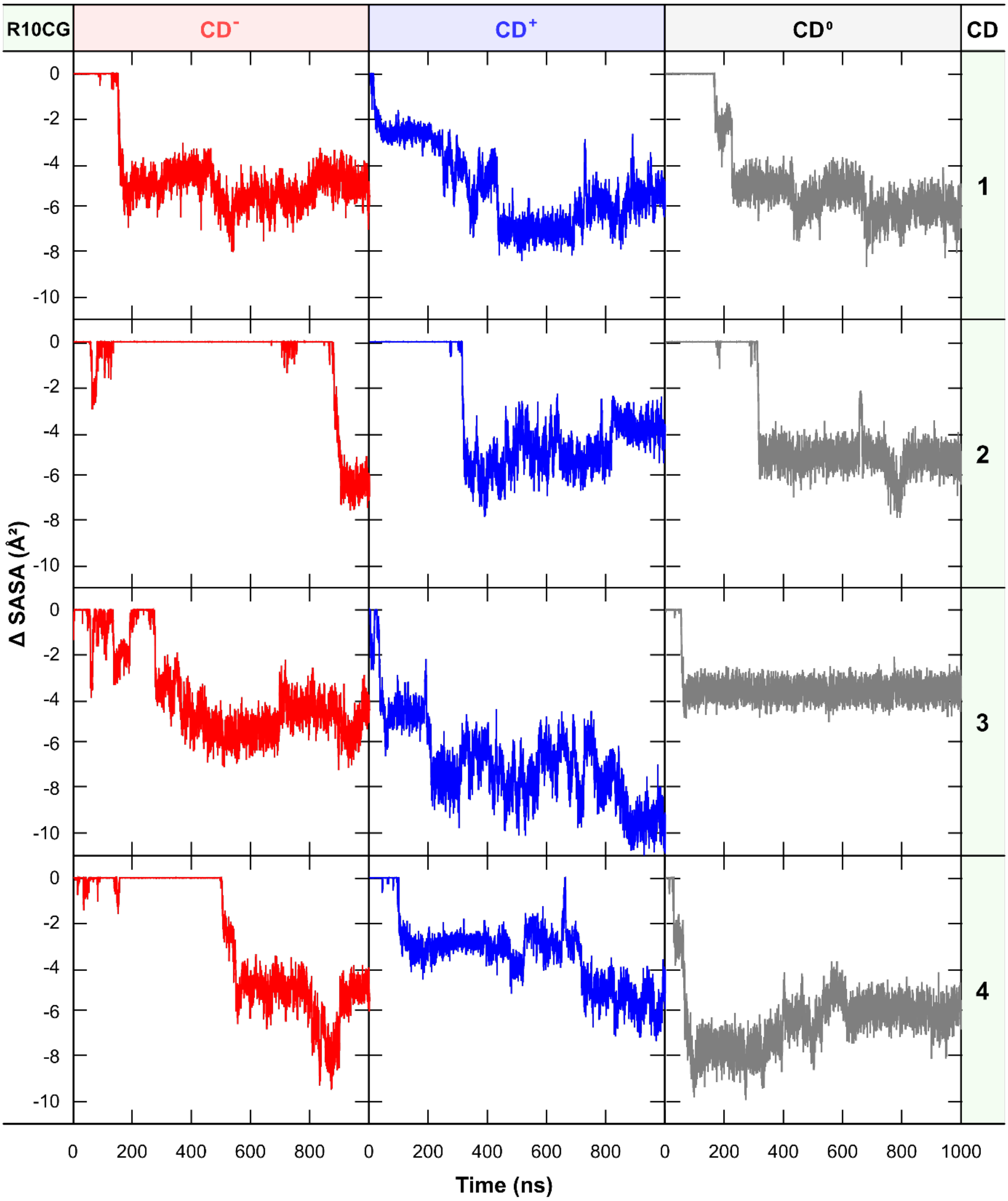

***Figure S19:** ΔSASA evolution in **R10CG** system. ΔSASA corresponds to the difference between SASA calculated for the whole NA+CD complex and the sum of SASA of NA and CD separately.*

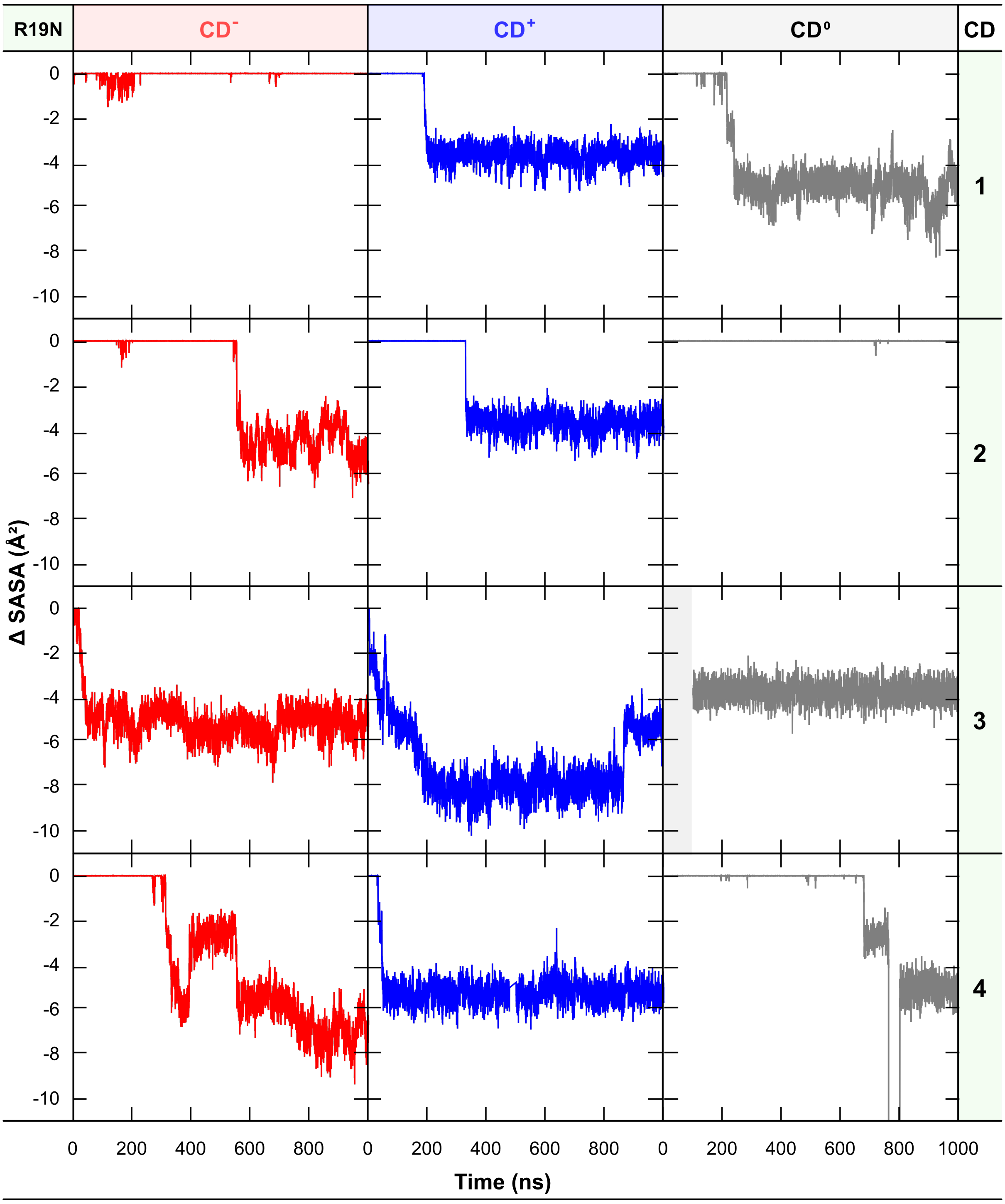

***Figure S20:** *ΔSASA evolution in **R19N** system.* ΔSASA corresponds to the difference between SASA calculated for the whole NA+CD complex and the sum of SASA of NA and CD separately. The first 100 ns of replicate 3 of CD^0 simulation is lacking data. A part of replicate 4 of CD^0 simulation (750-800 ns) includes an artifact and is not displayed.*

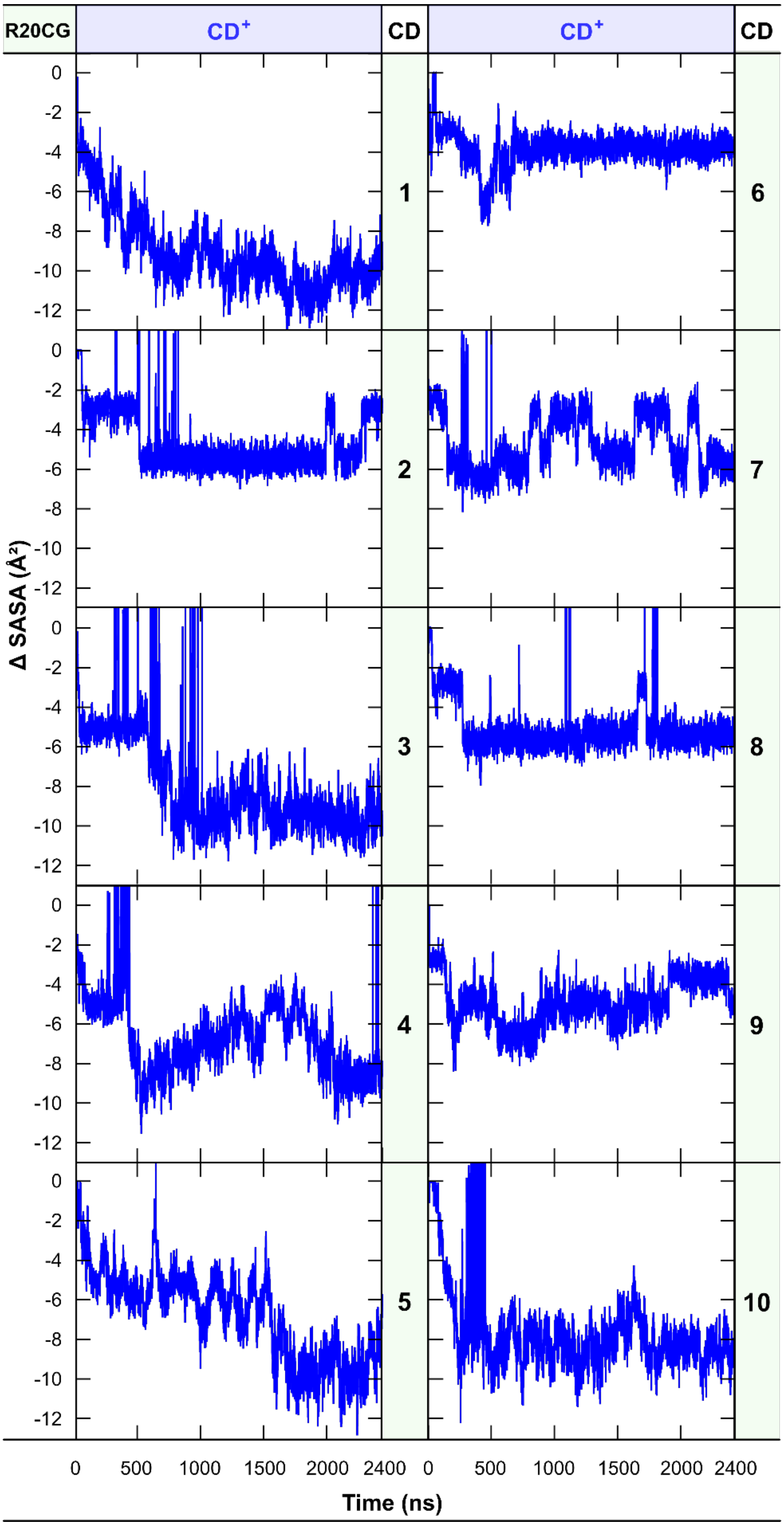

Figure S21: *ΔSASA evolution in* ***R20CG*** *system. ΔSASA corresponds to the difference between SASA calculated for the whole NA+CD complex and the sum of SASA of NA and CD separately.*

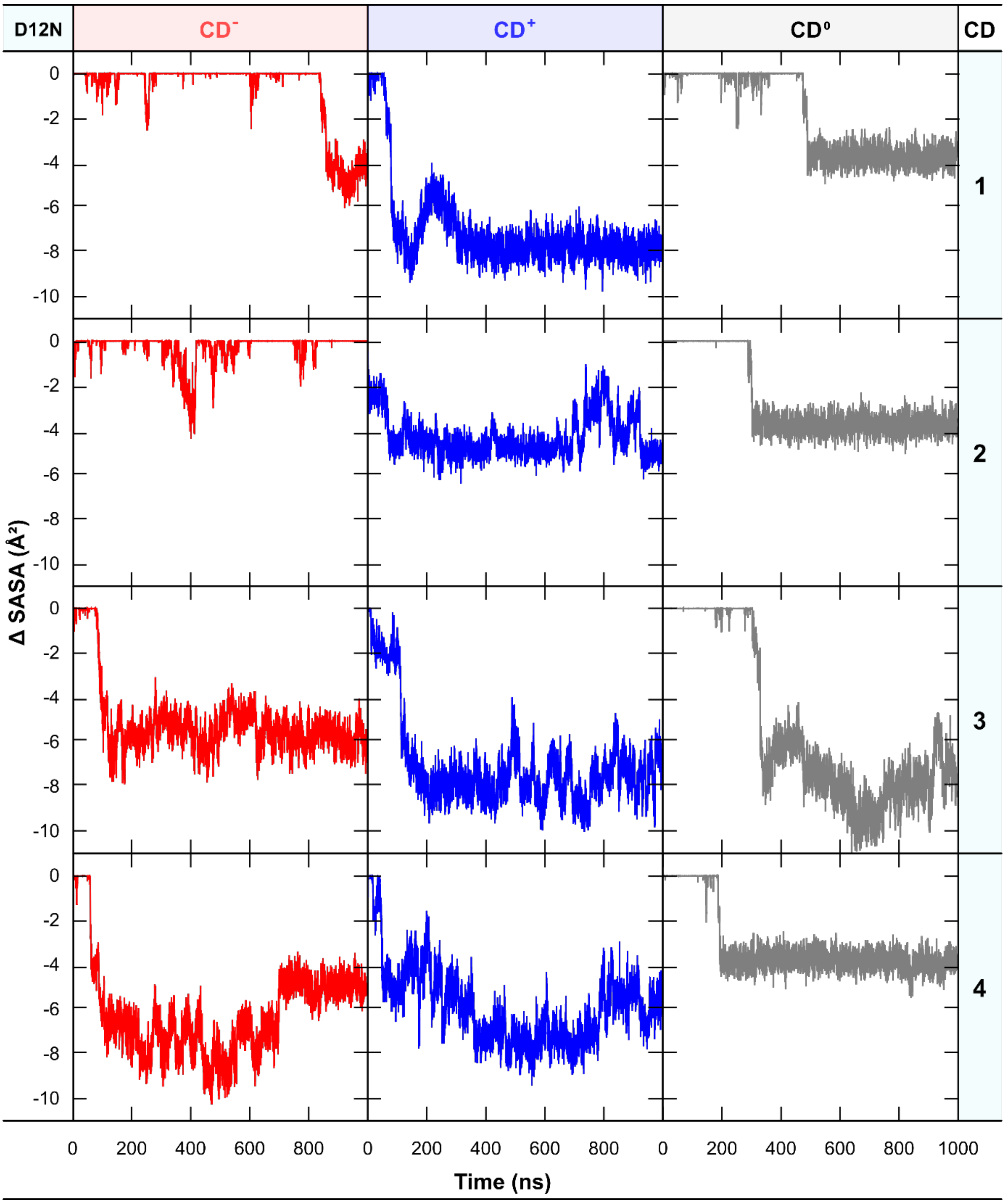

Figure S22: *ΔSASA evolution in* ***D12N*** *system. ΔSASA corresponds to the difference between SASA calculated for the whole NA+CD complex and the sum of SASA of NA and CD separately.*

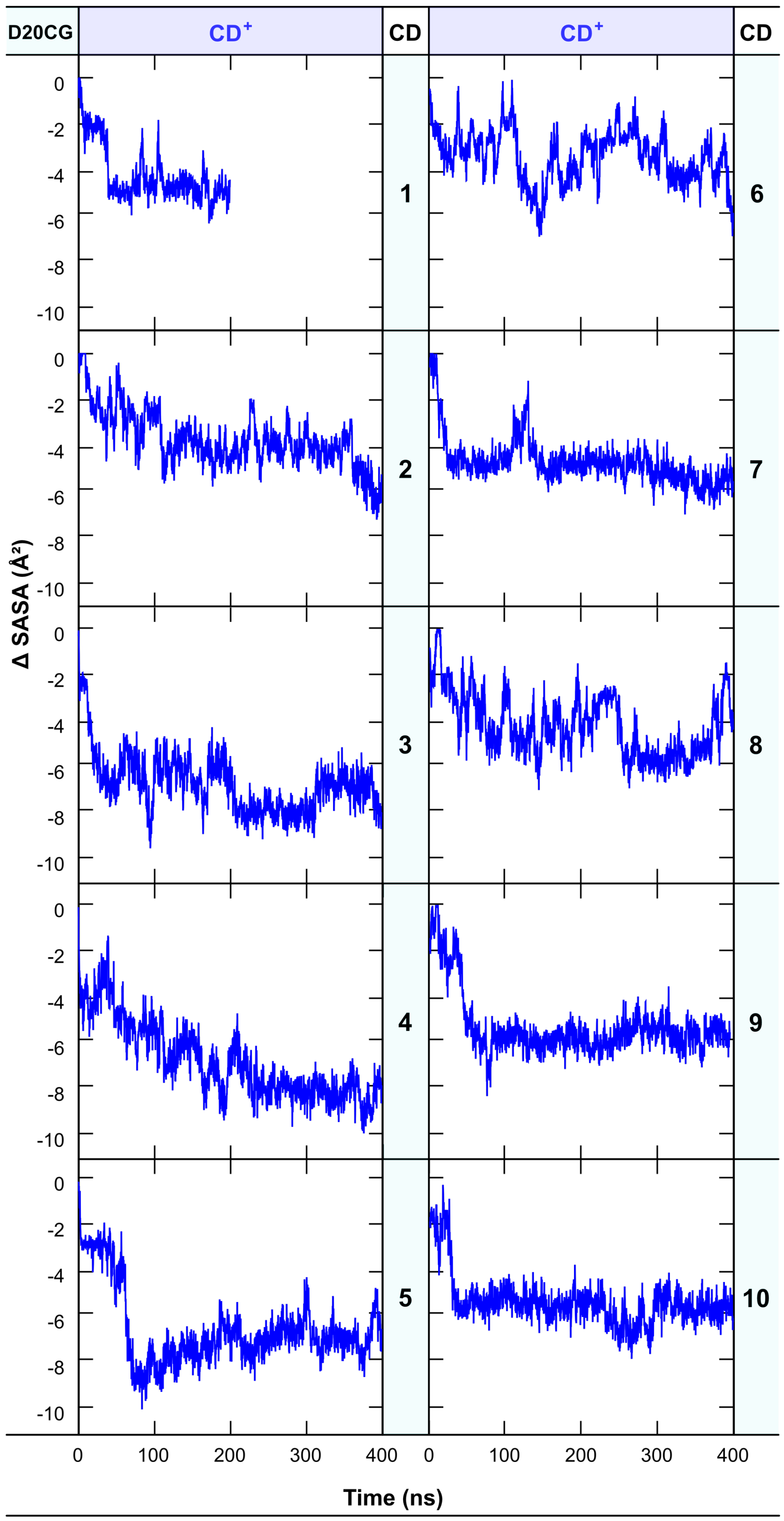

Figure S23: ΔSASA evolution in ***D20CG*** *system. ΔSASA corresponds to the difference between SASA calculated for the whole NA+CD complex and the sum of SASA of NA and CD separately. The last 200 ns of replicate 1 is lacking data.*

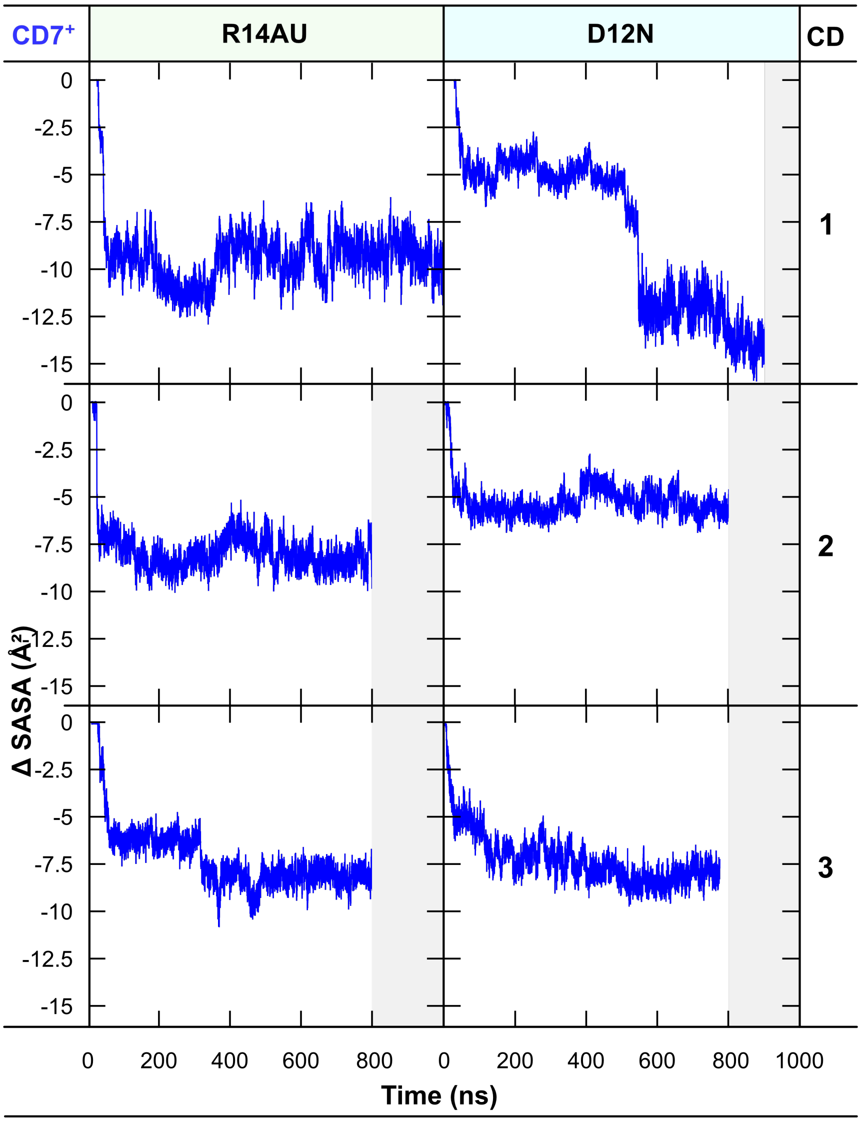

Figure S24: *ΔSASA evolution in canonical systems with CD7$^+$. ΔSASA corresponds to the difference between SASA calculated for the whole NA+CD complex and the sum of SASA of NA and CD separately.*

Interaction modes between CD and non-canonical NA structures

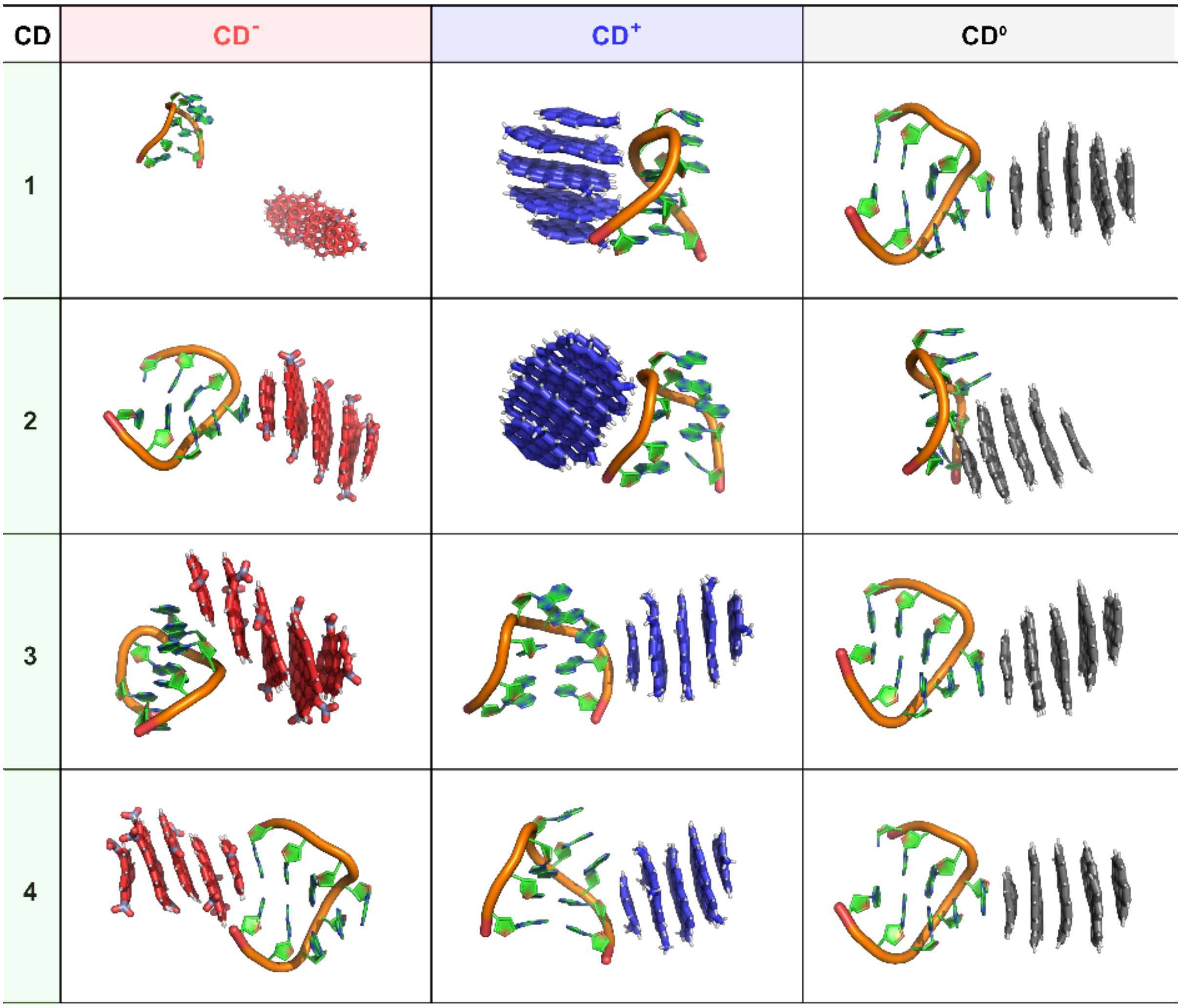

Figure S25: *Final interaction modes of **TL** with carbon dots.*

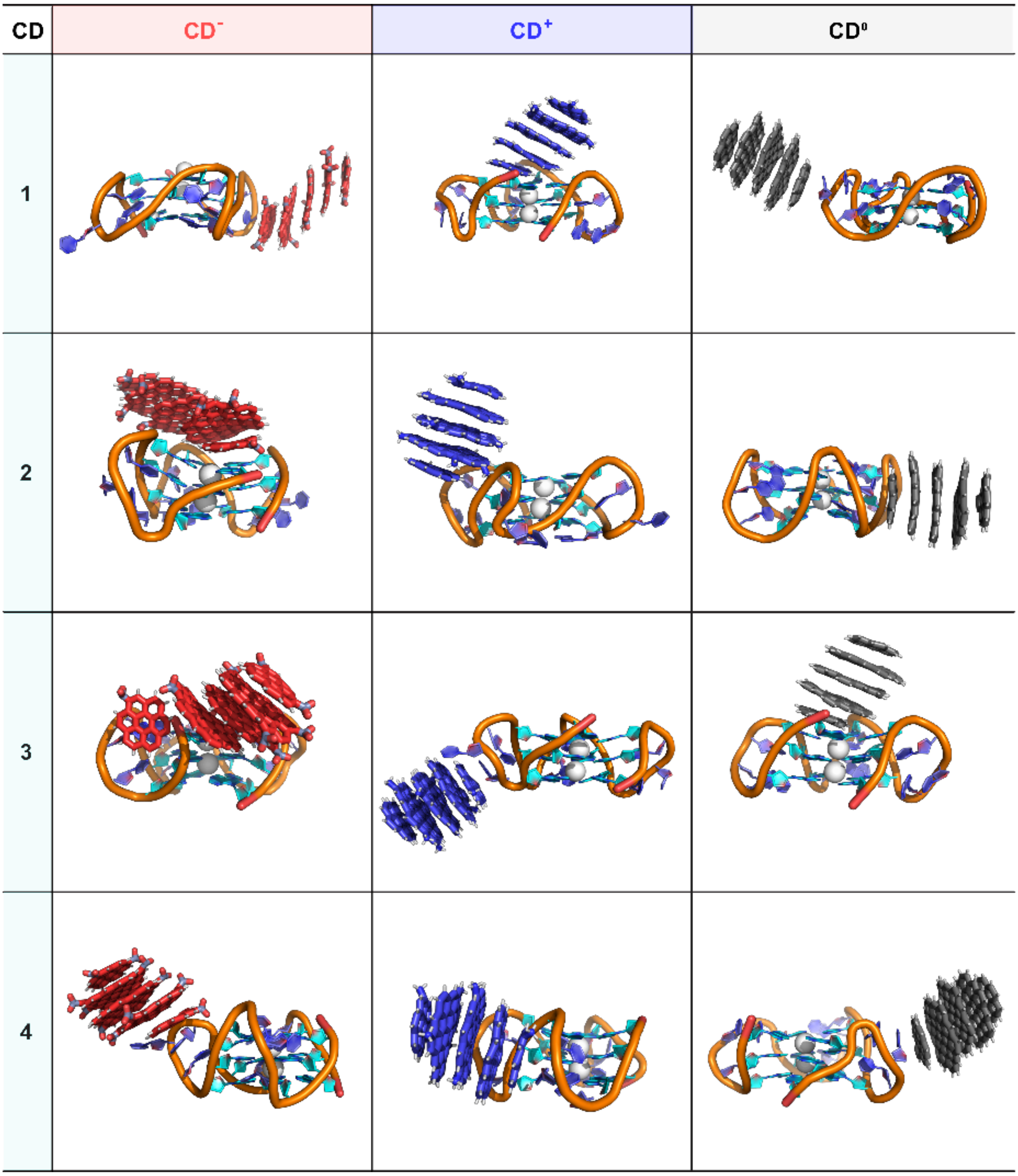

***Figure S26:** Final interaction modes of **hG4** with carbon dots.*

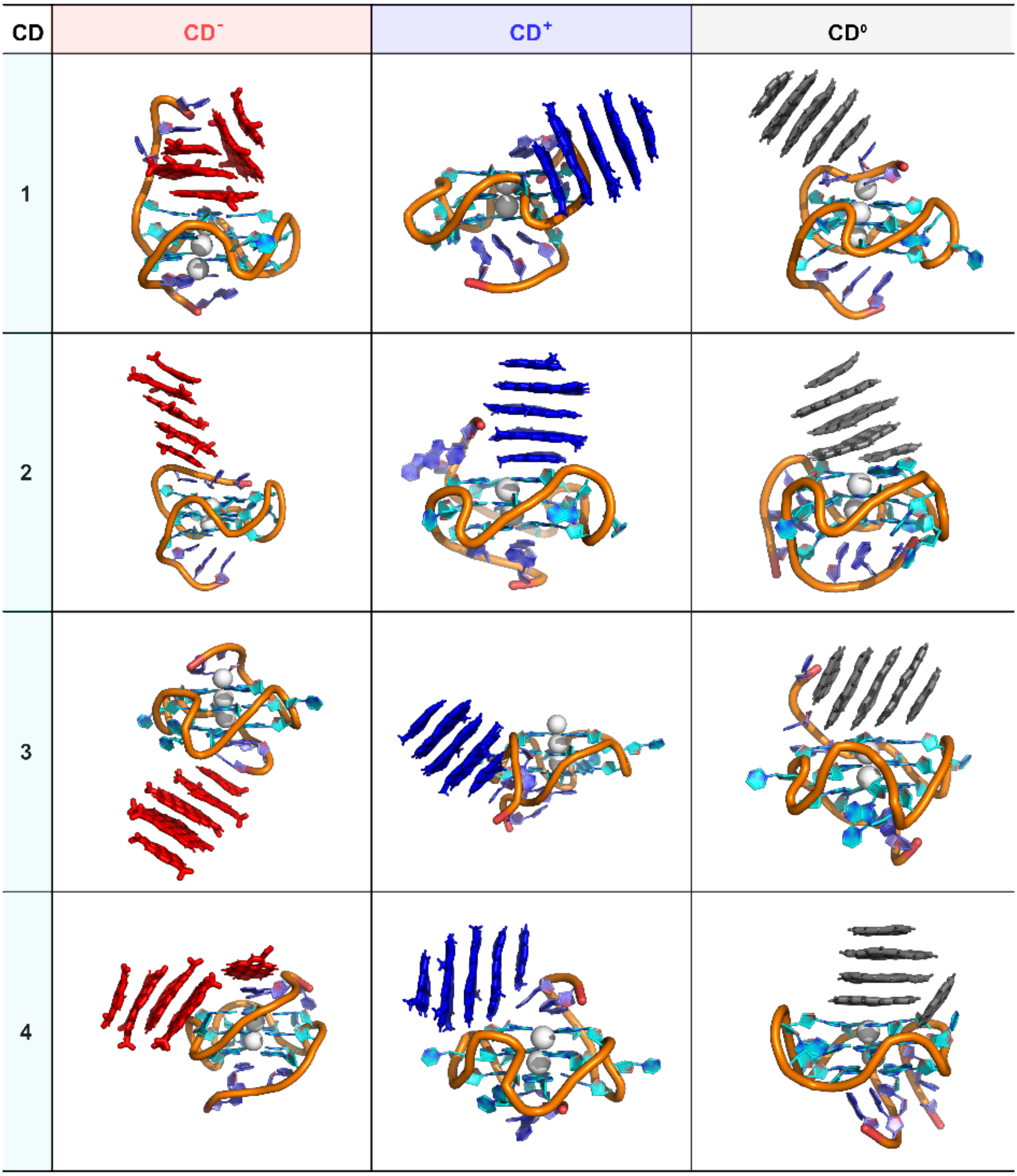

Figure S27: *Final interaction modes of* ***cG4*** *with carbon dots.*

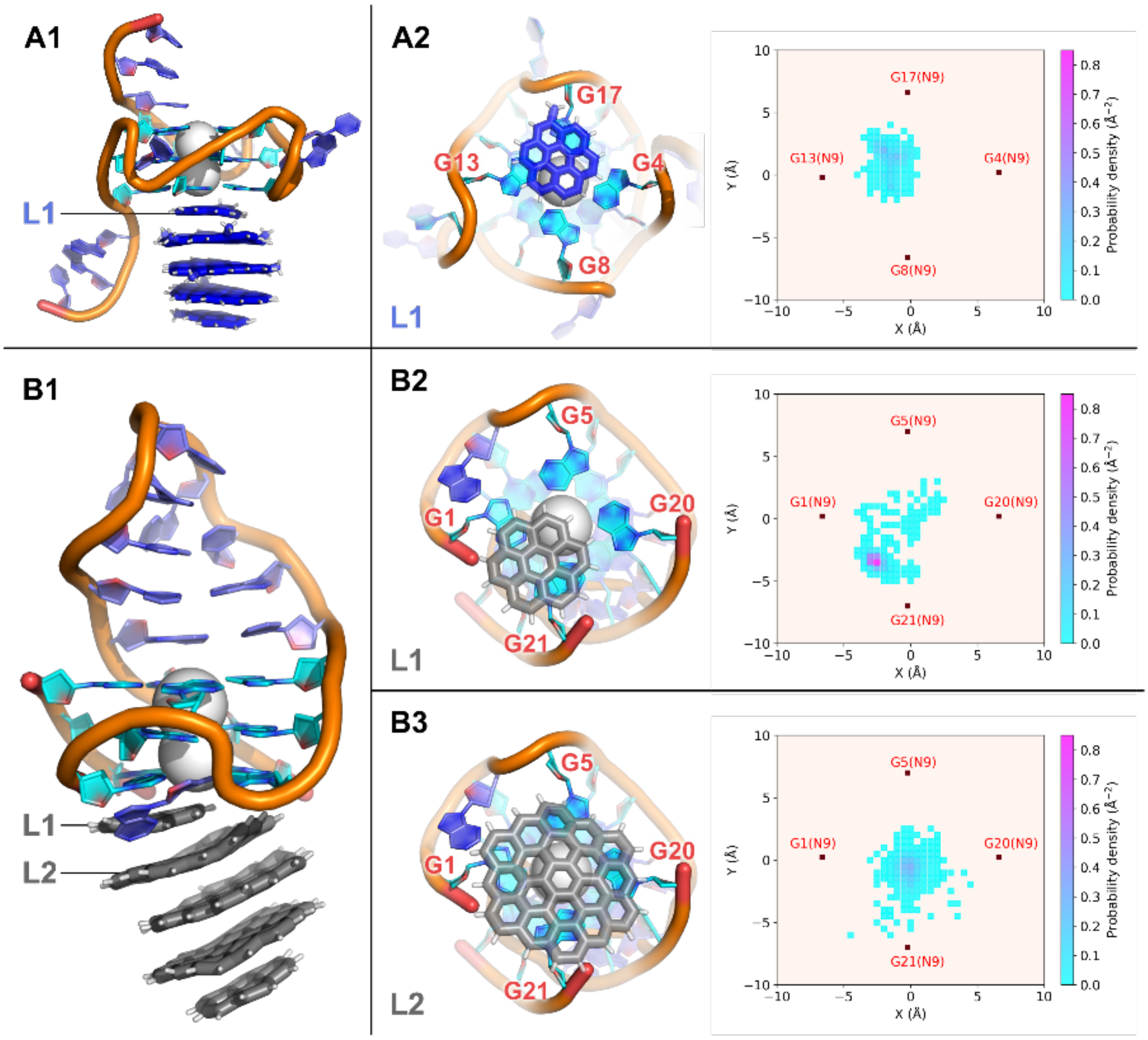

Figure S28: *Position of CD stacked on a tetrad. A) CD^{+} stacked on the 5'-tetrad of cG4; Panel A1 depicts the whole complex, while A2 is bottom view focused on the slightly off-centered position of L1. B) CD^{0} stacked on the 5'-tetrad of QDJ. Panel B1 depicts the whole complex, while B2 is bottom view focused on the off-centered position of L1, and B3 shows L2 almost perfectly aligned with the central channel. The graphs on the right indicate the position of a given CD layer's center projected onto the adjacent tetrad. The shown structures correspond to the point of highest density and best direction fit in the graphs.*

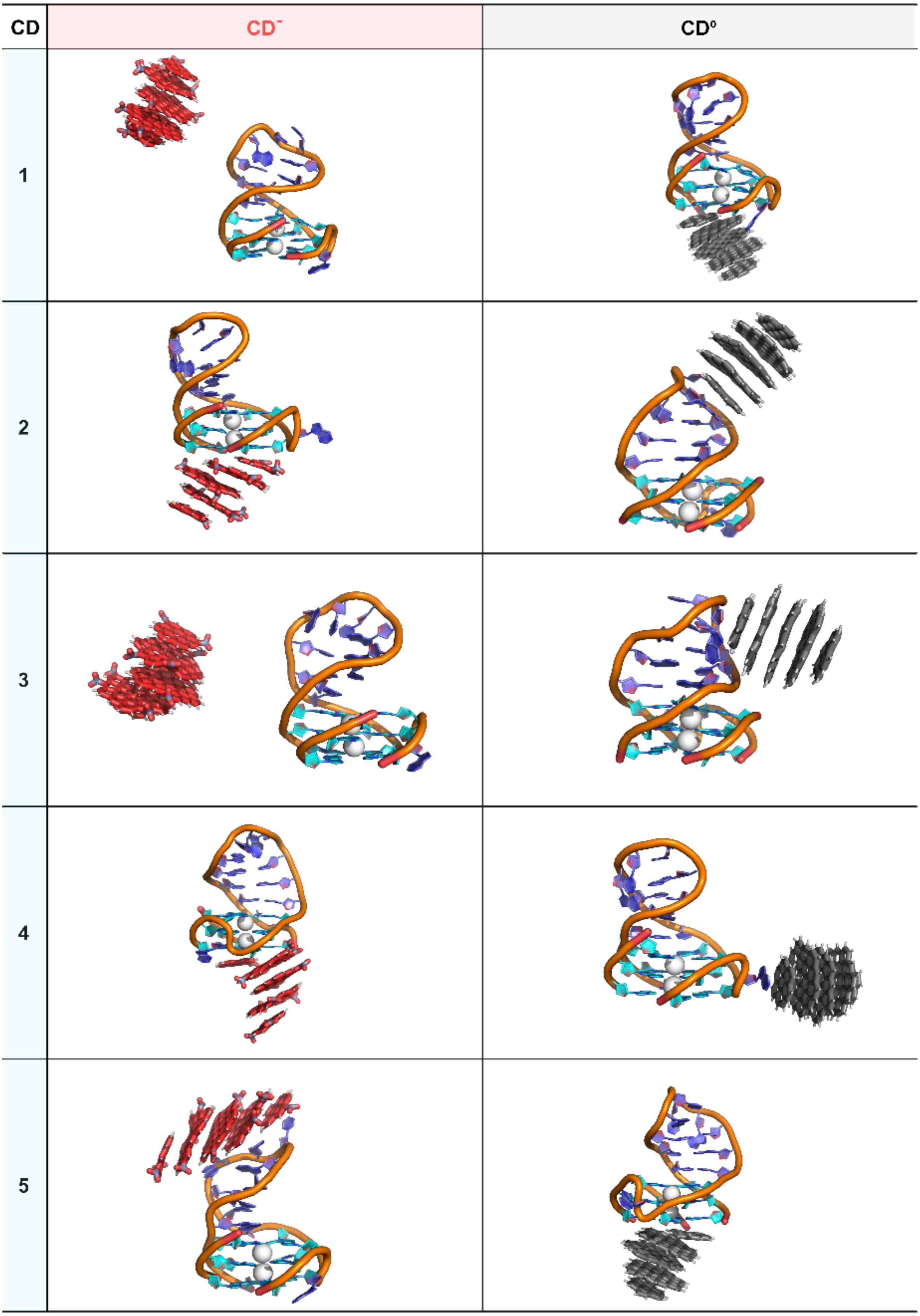

Figure S29: *Final interaction modes of **QDJ** with* CD^- *and* CD^0.

CD	CD⁺	CD	CD⁺
1	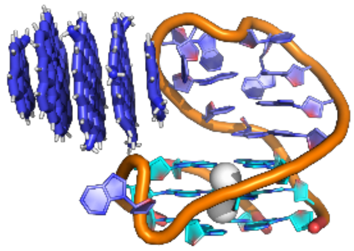	6	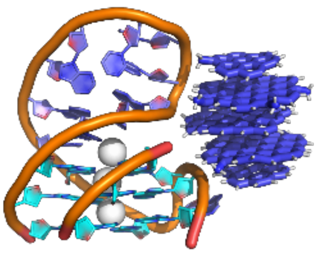
2	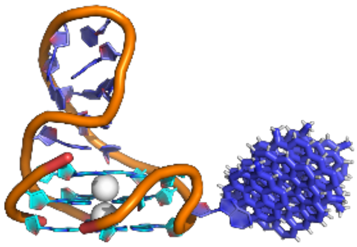	7	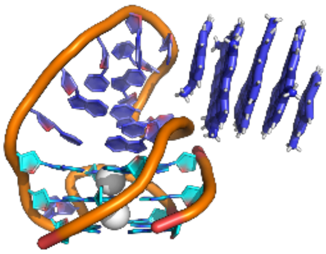
3	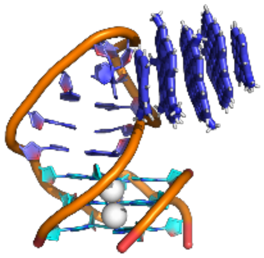	8	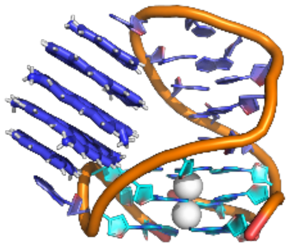
4	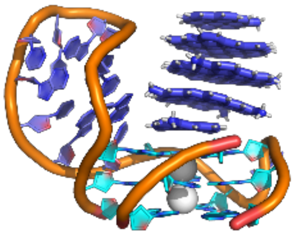	9	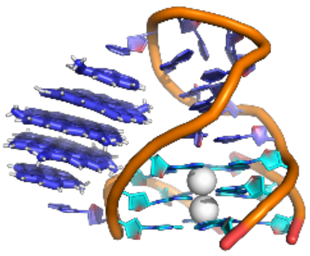
5	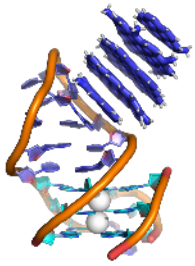	10	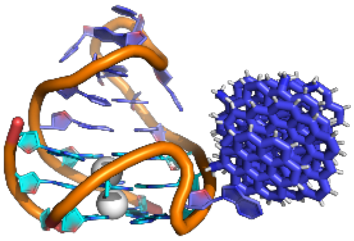

Figure S30: *Final interaction modes of* ***QDJ*** *with CD⁺.*

CD	hG4	cG4	QDJ
1	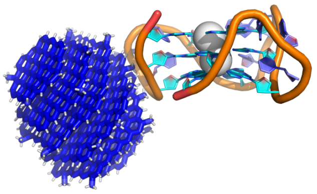	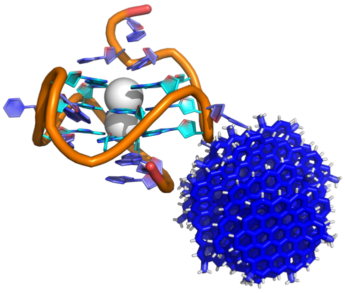	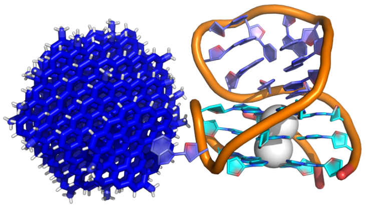
2	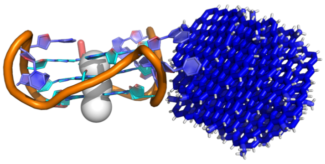	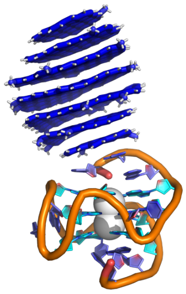	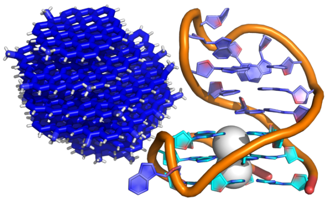
3	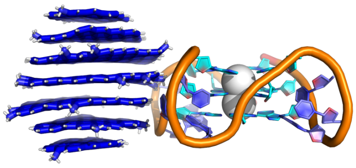	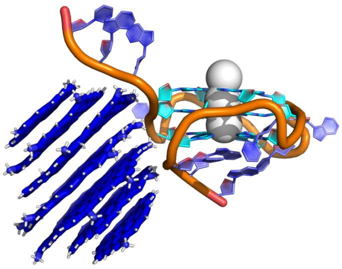	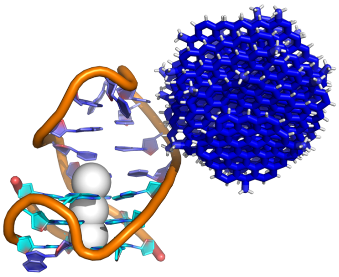

Figure S31: *Final interaction modes of non-canonical DNAs with CD7^{+}.*

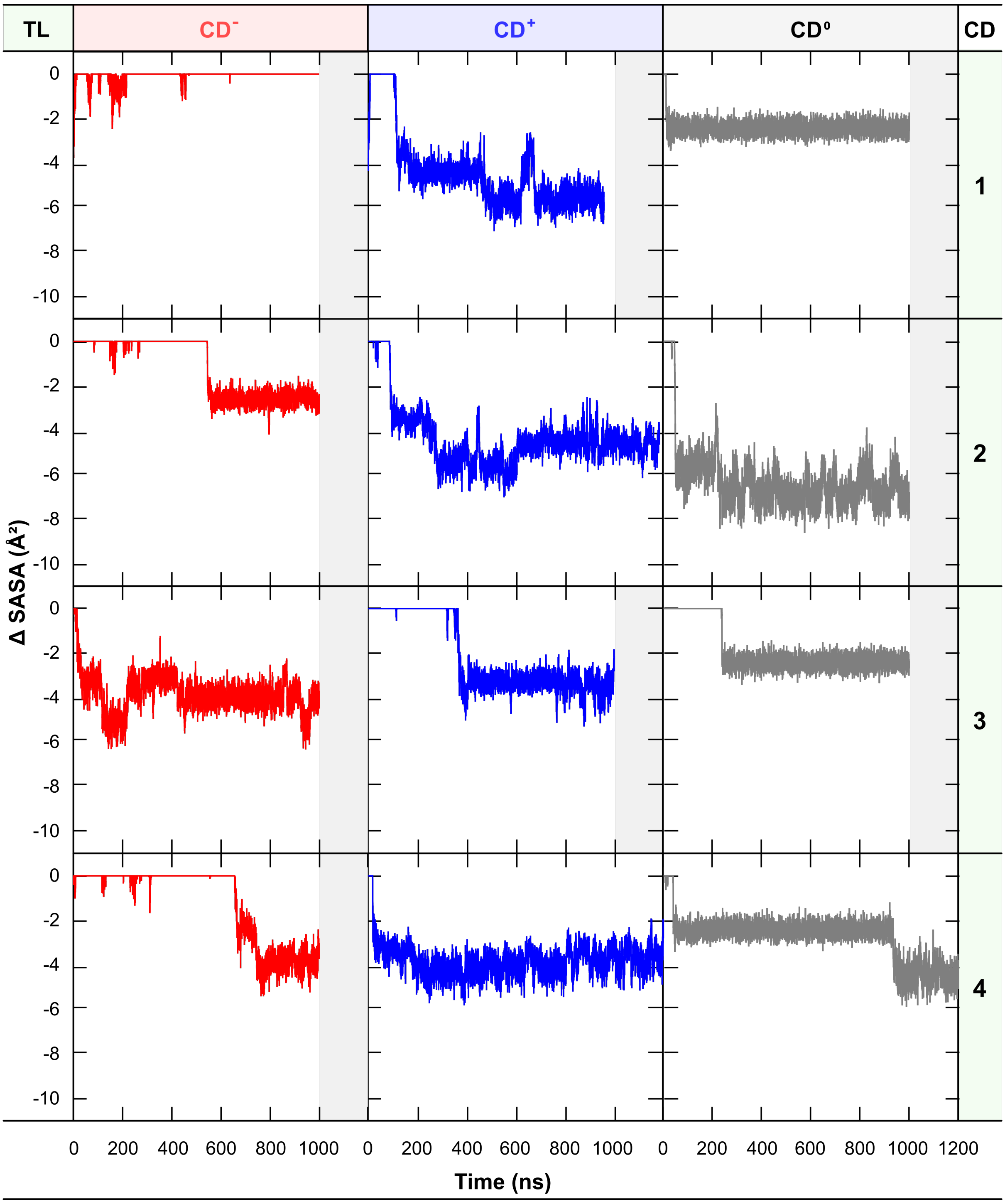

***Figure S32:** ΔSASA evolution in **TL** system. ΔSASA corresponds to the difference between SASA calculated for the whole NA+CD complex and the sum of SASA of NA and CD separately.*

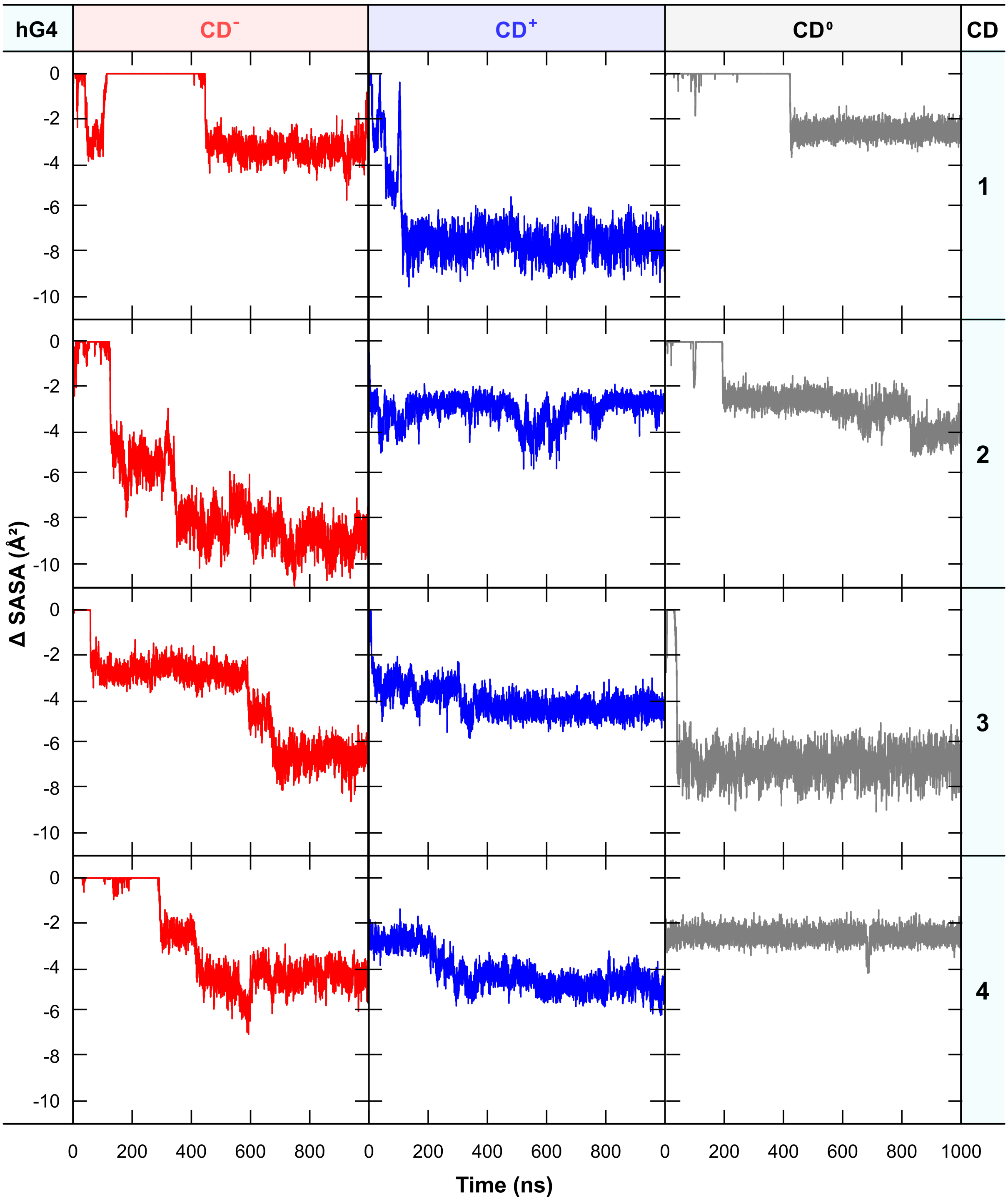

Figure S33: *ΔSASA evolution in* ***hG4*** *system. ΔSASA corresponds to the difference between SASA calculated for the whole NA+CD complex and the sum of SASA of NA and CD separately.*

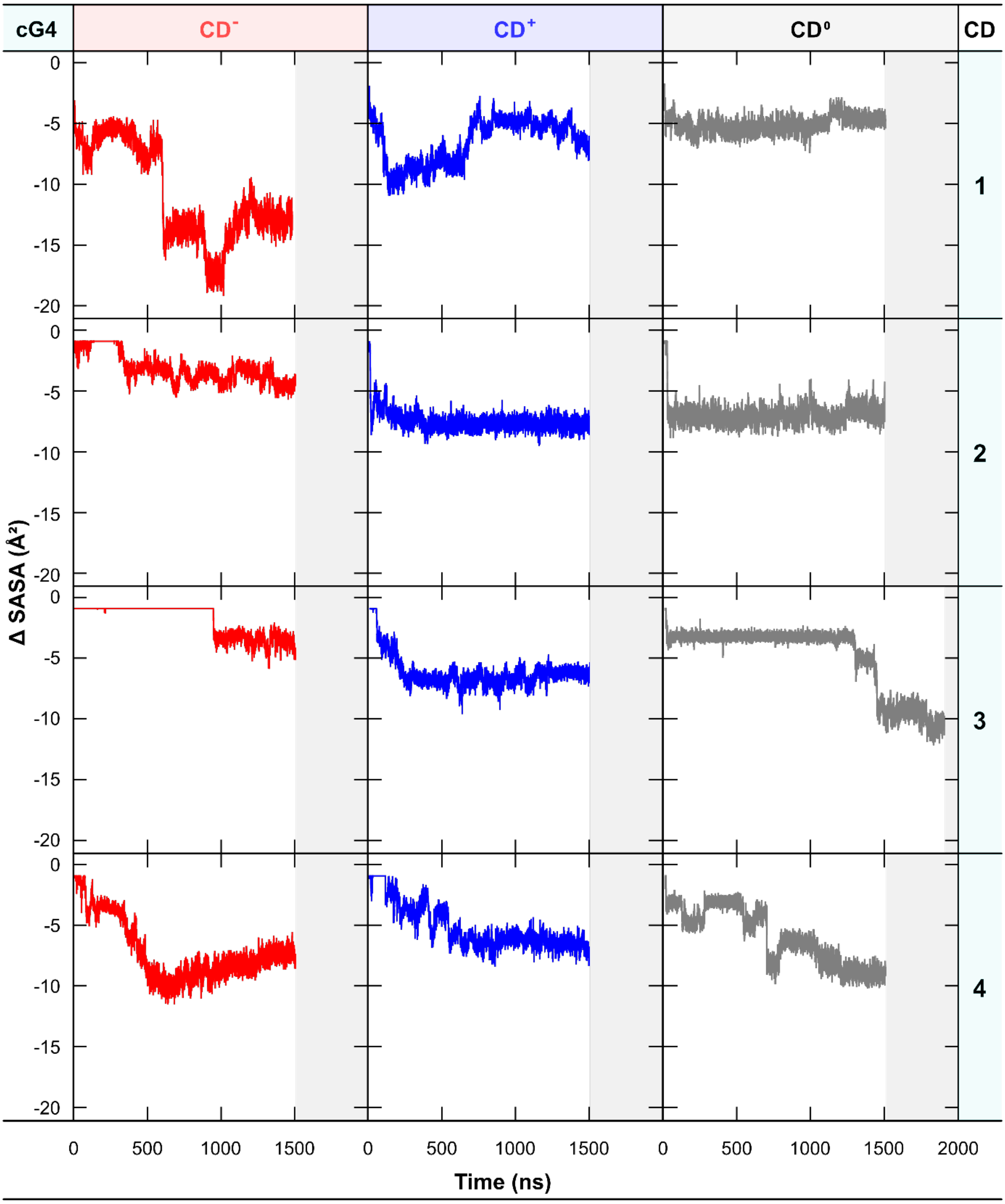

Figure S34: *ΔSASA evolution in* ***cG4*** *system. ΔSASA corresponds to the difference between SASA calculated for the whole NA+CD complex and the sum of SASA of NA and CD separately.*

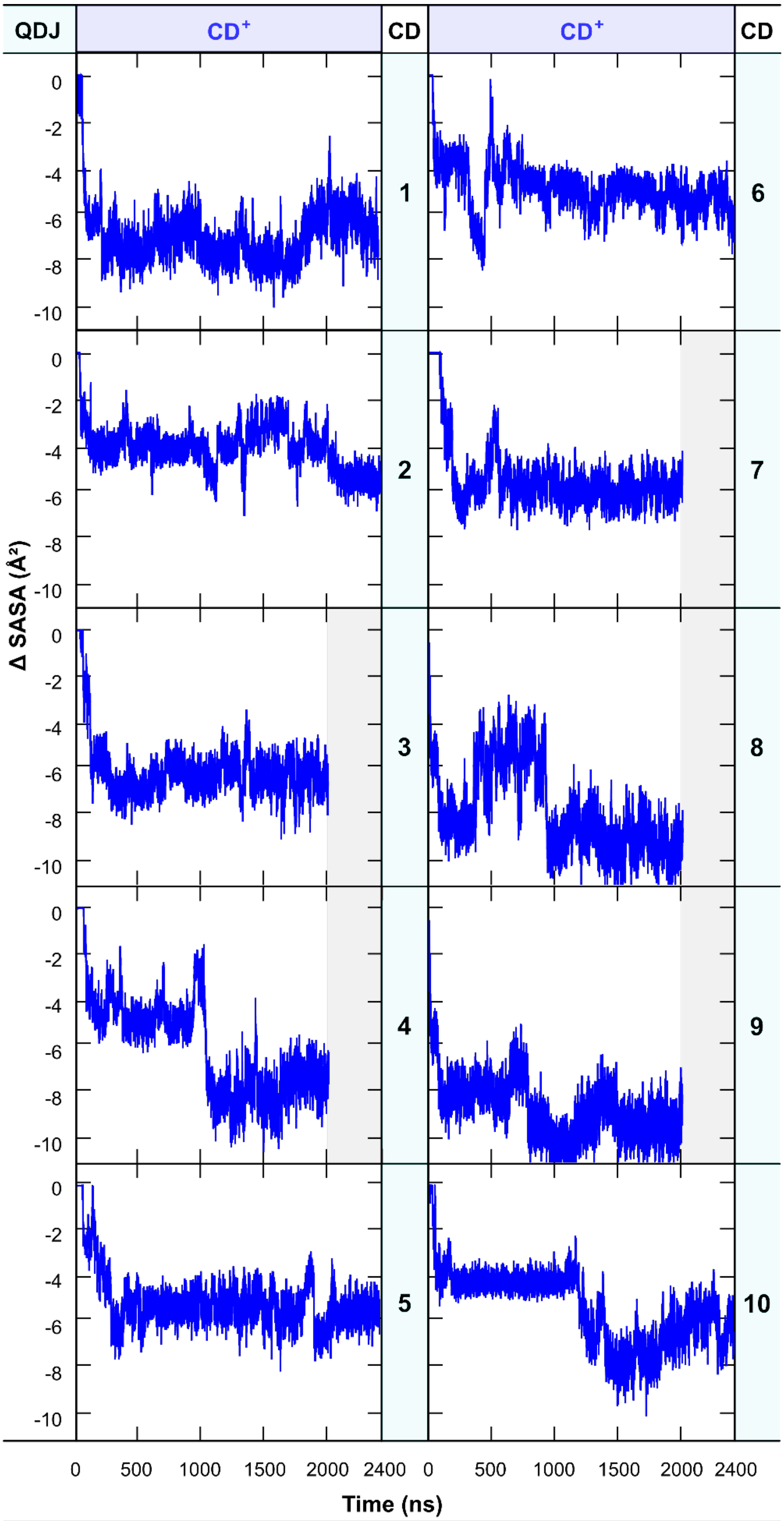

***Figure S35:** ΔSASA evolution in **QDJ** system with CD^{+}. ΔSASA corresponds to the difference between SASA calculated for the whole NA+CD complex and the sum of SASA of NA and CD separately.*

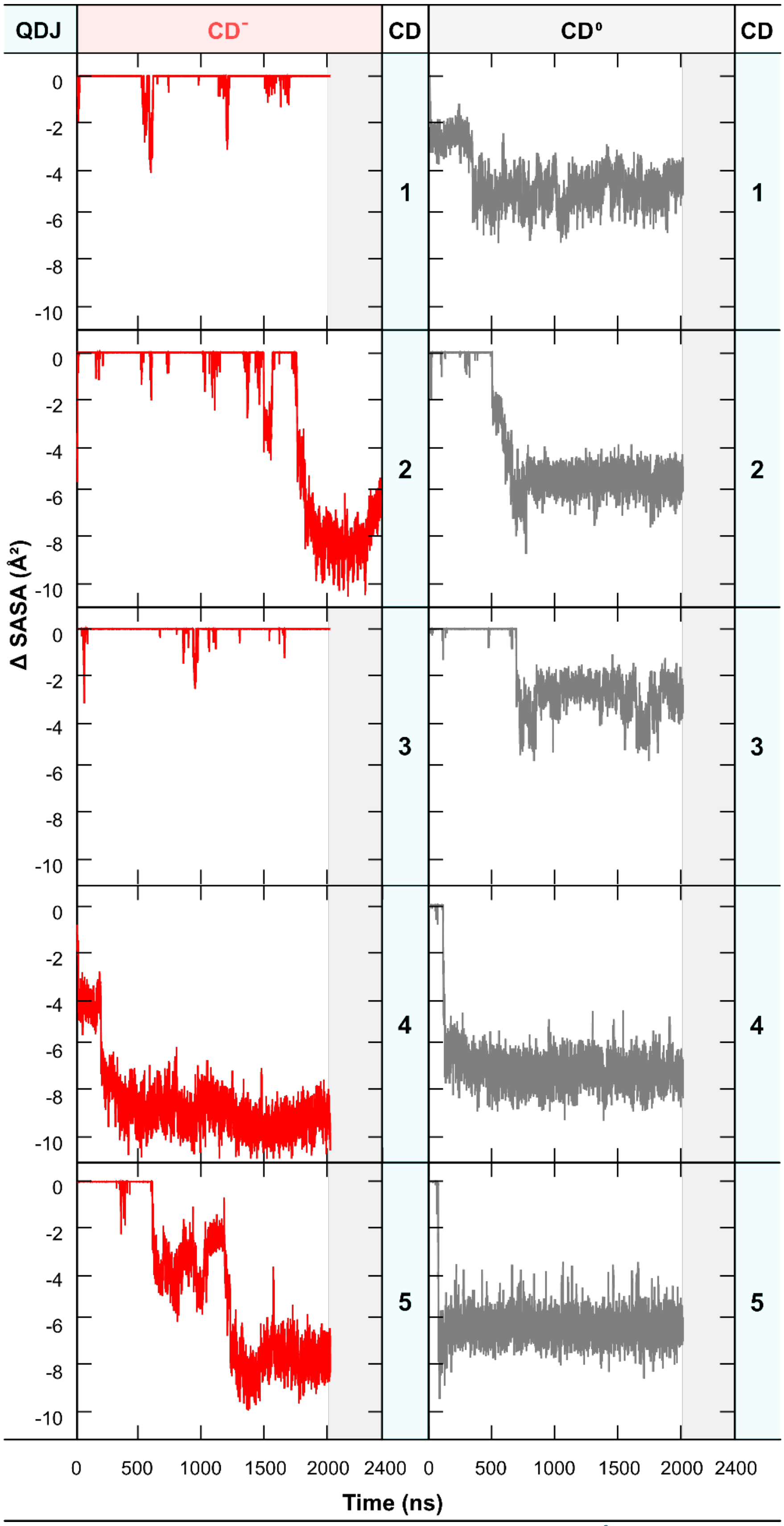

Figure S36: *ΔSASA evolution in* ***QDJ*** *system with CD⁻ and CD⁰. ΔSASA corresponds to the difference between SASA calculated for the whole NA+CD complex and the sum of SASA of NA and CD separately*

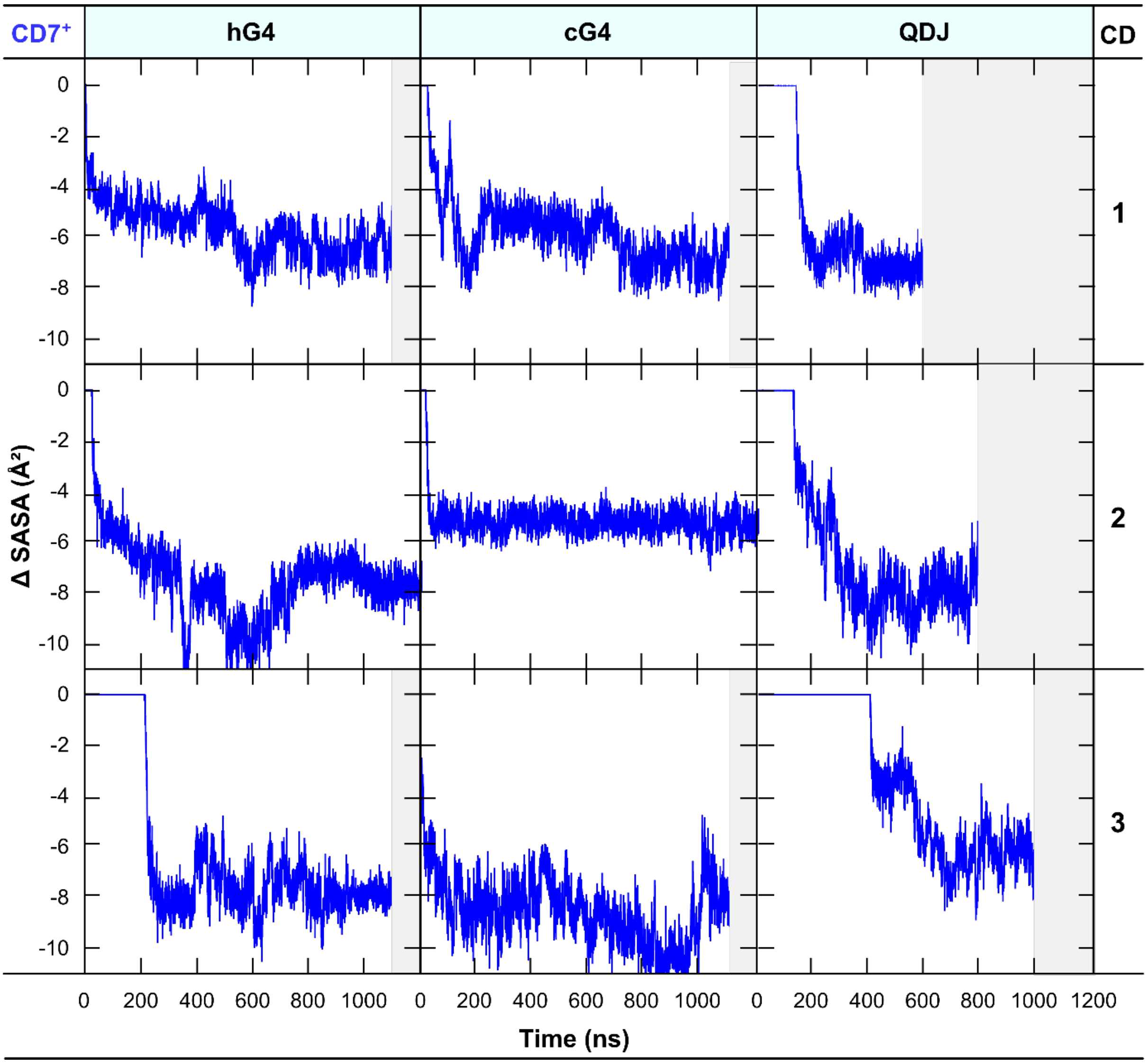

Figure S37: *ΔSASA evolution in non-canonical systems with CD7+. ΔSASA corresponds to the difference between SASA calculated for the whole NA+CD complex and the sum of SASA of NA and CD separately.*

Nucleosome

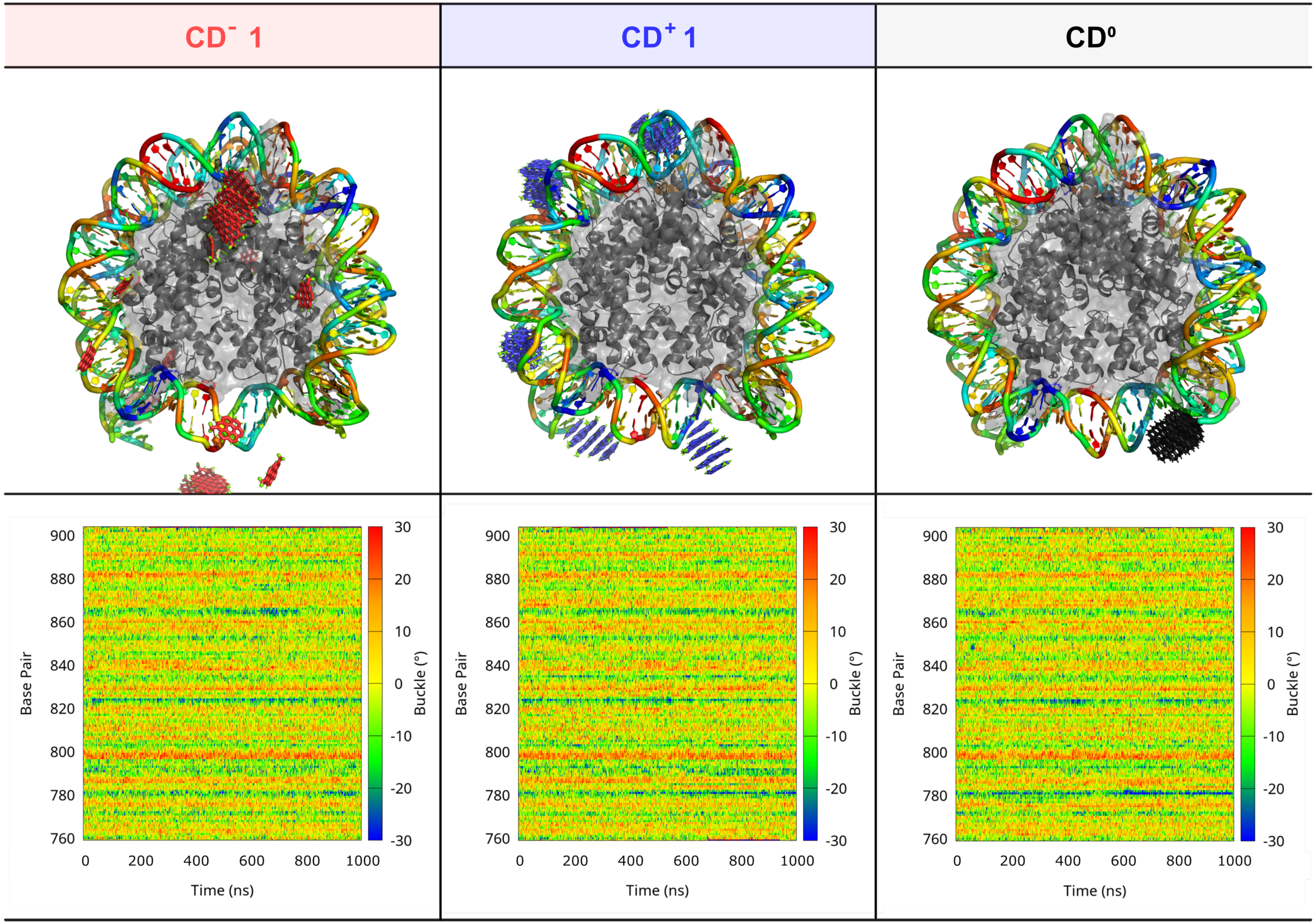

Figure S38: *Final structures of NS with bound CD with DNA residues colored by average buckle parameters (upper panel) and the heatmaps showing the time evolution of the per-residue buckle parameter (lower panel).*

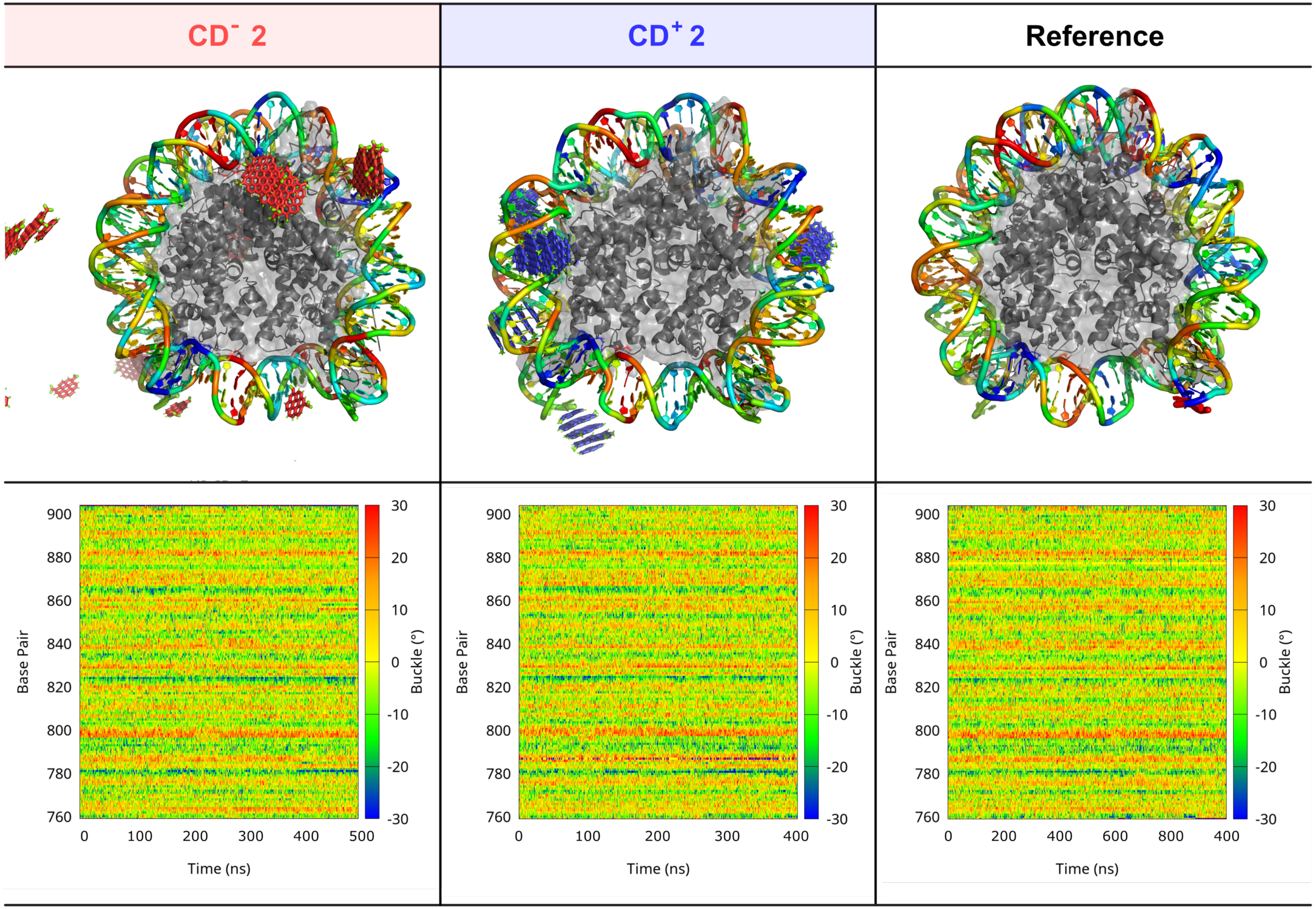

Figure S39: *Final structures of NS with bound CD with DNA residues colored by average buckle parameters (upper panel) and the heatmaps showing the time evolution of the per-residue buckle parameter (lower panel).*

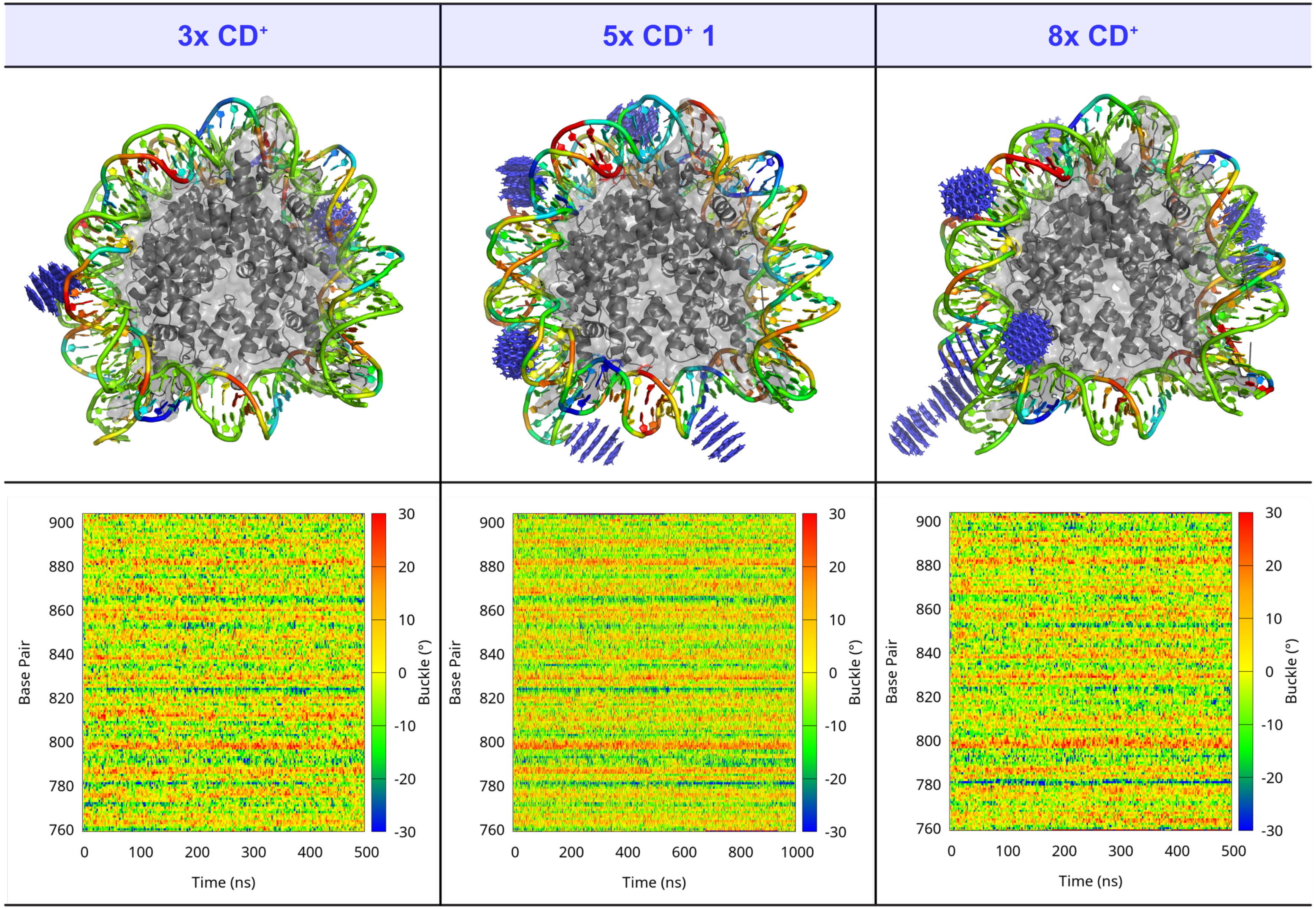

Figure S40: *Final structures of NS with bound CD with DNA residues colored by average buckle parameters (upper panel) and the heatmaps showing the time evolution of the per-residue buckle parameter (lower panel). The 5 CD*$^{+}$ *1 is the simulation from Figure S38 shown for reference.*

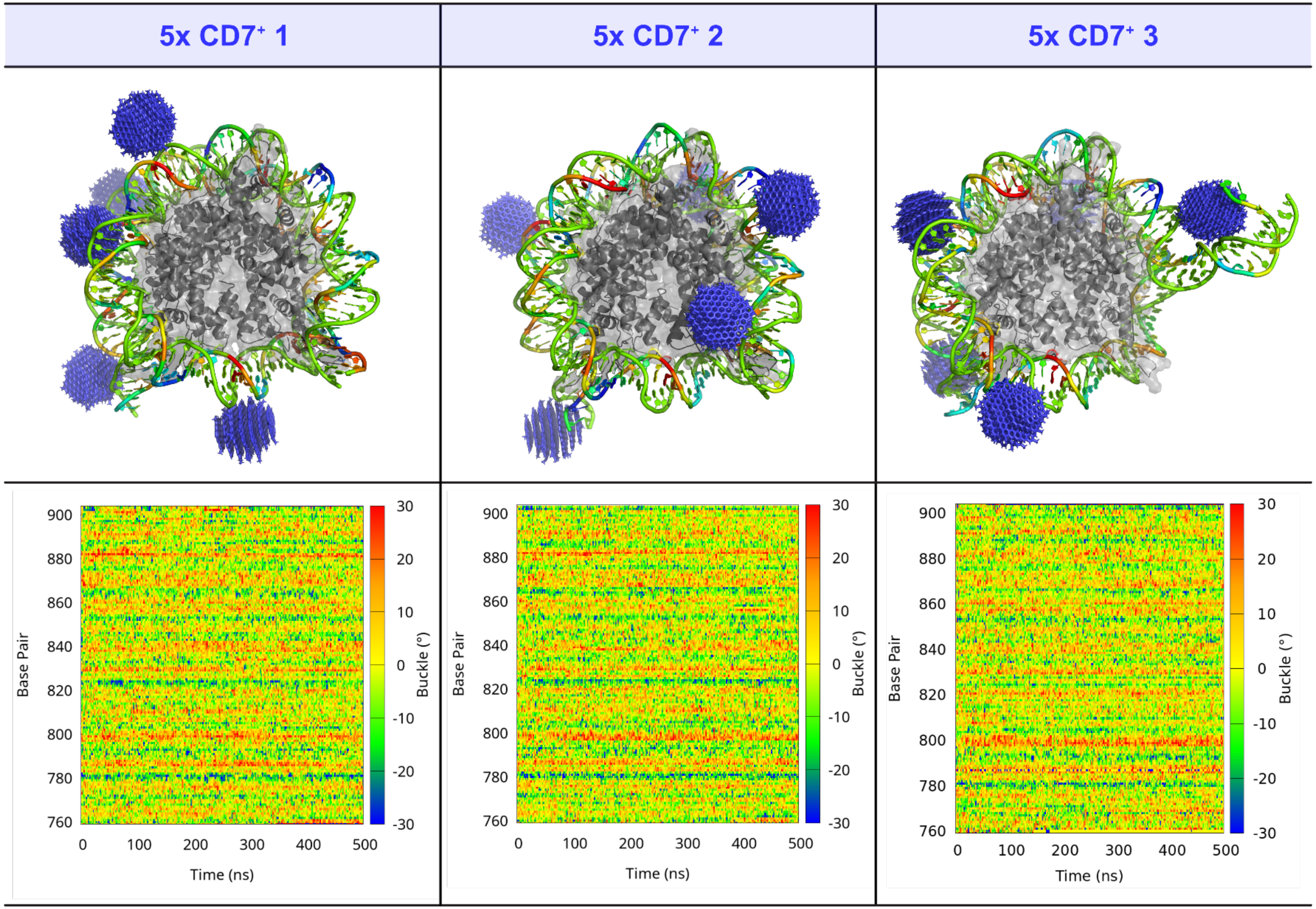

Figure S41: *Final structures of NS with bound CD with DNA residues colored by average buckle parameters (upper panel) and the heatmaps showing the time evolution of the per-residue buckle parameter (lower panel).*

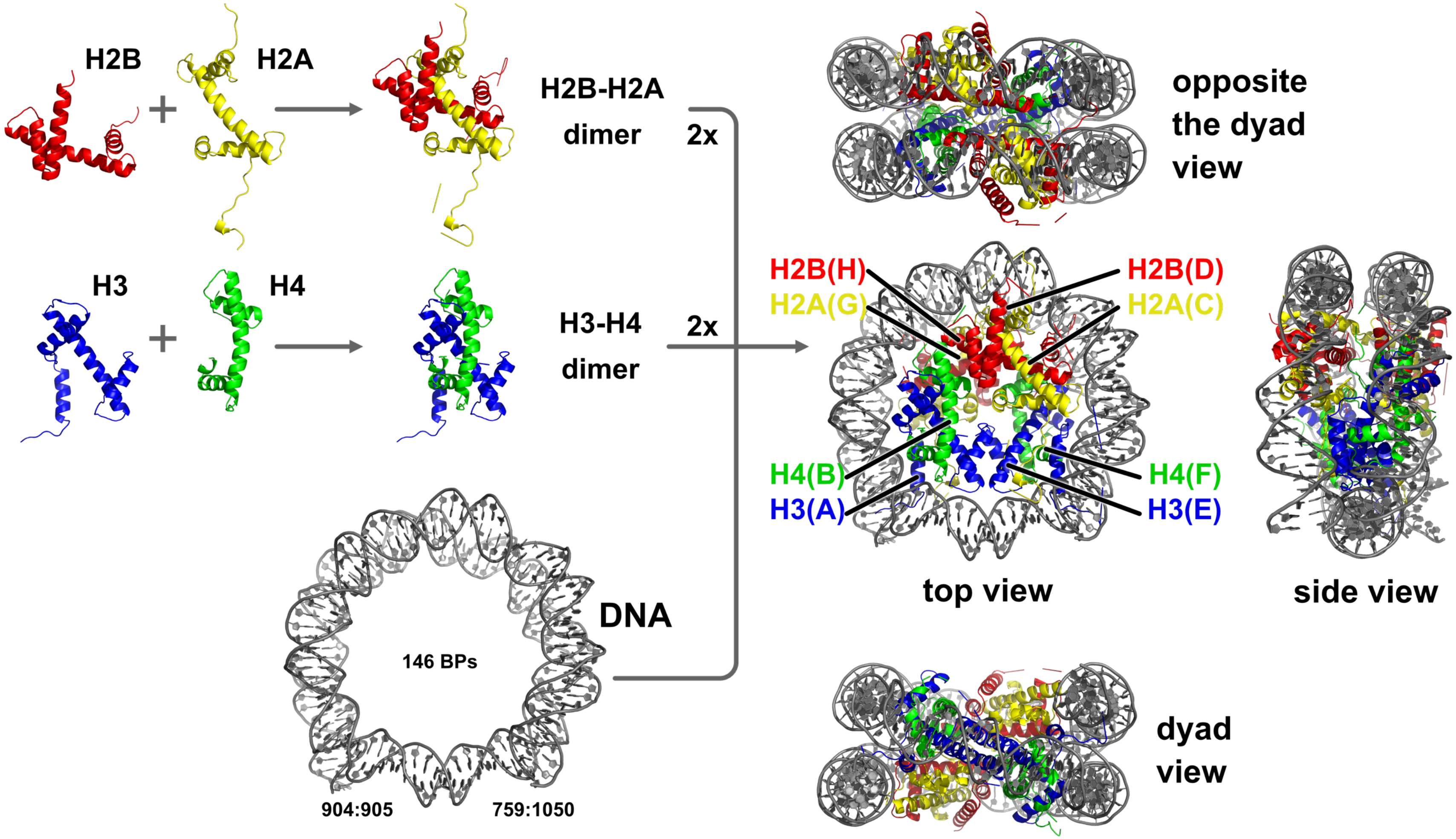

Figure S42: *Structure of NS with a focus on histone. DNA of 146 base pairs (BPs) wraps around a histone, that is composed of eight subunits arranged into four dimers (left). Dimers composed of H3 and H4 are more conserved (see Figures S43, S44 left) and are localized close to dyad, while the dimers composed of H2A and H2B are more flexible (see Figures S43, S44 right).*

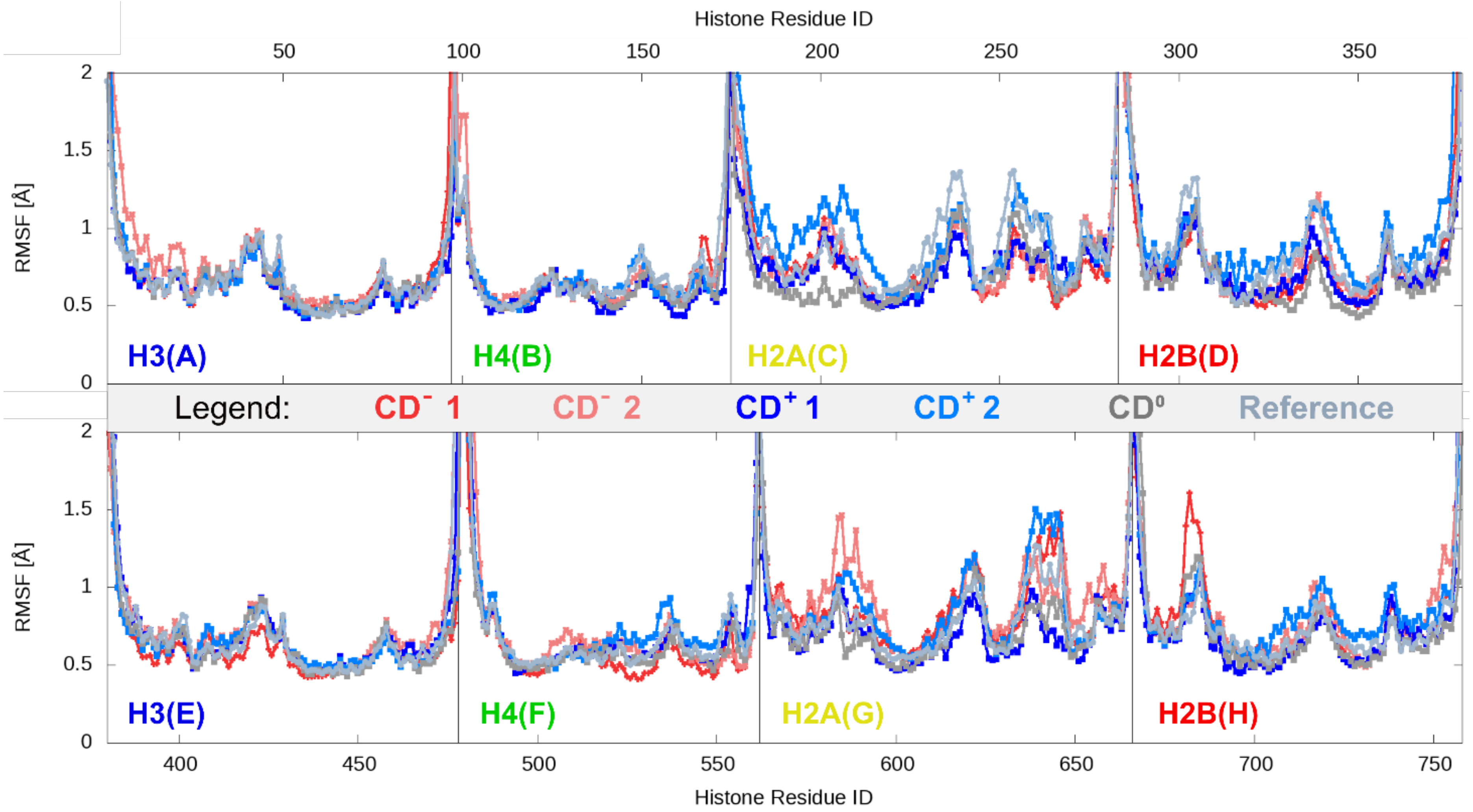

Figure S43: *Per-residue root mean square fluctuations (RMSF) of histone subunits. RMSF is calculated from the last 100 ns of MD simulation after fitting to the average structure using backbone heavy atoms (C, CA, N).*

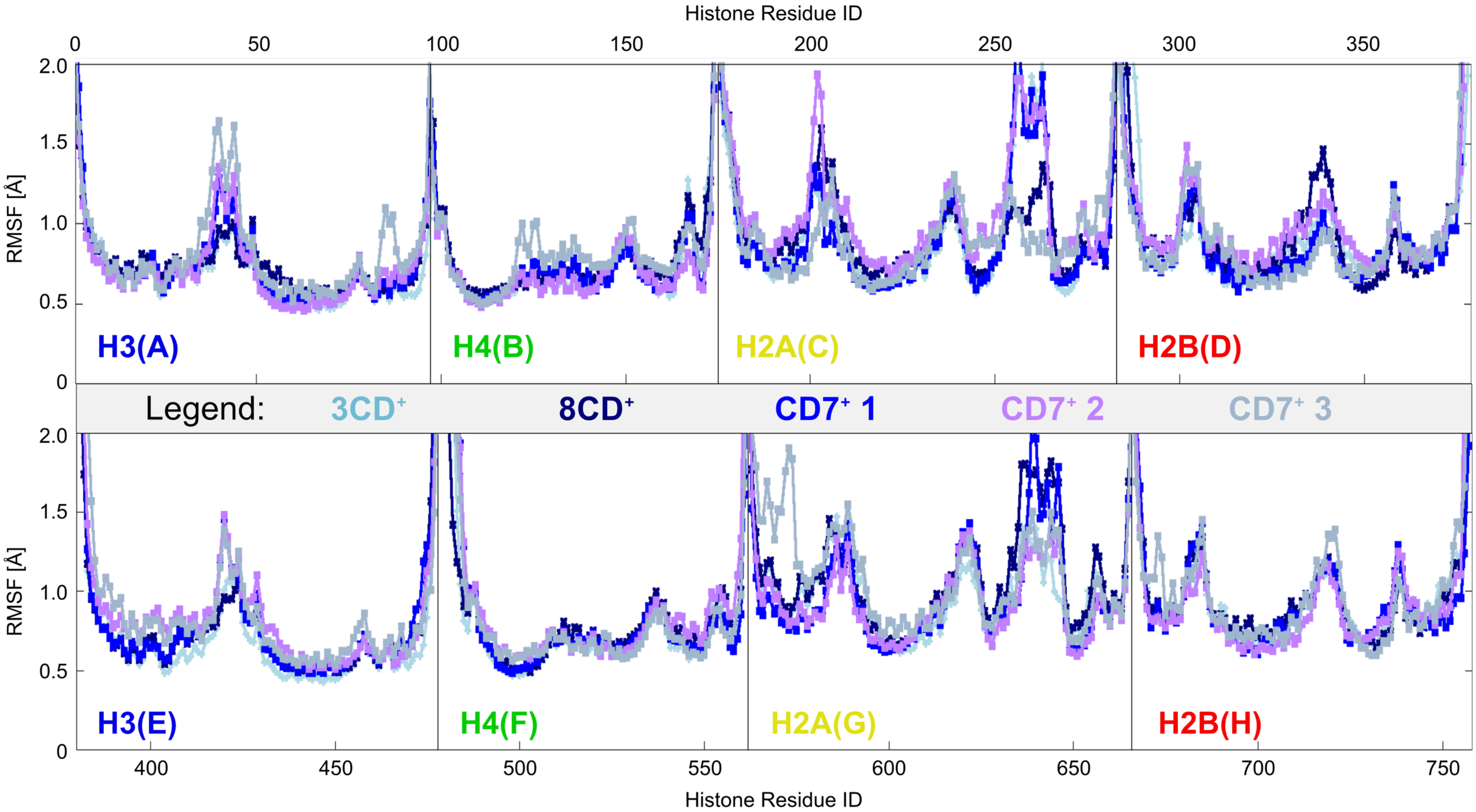

Figure S44: *Per-residue root mean square fluctuations (RMSF) of histone subunits. RMSF is calculated from the last 100 ns of MD simulation after fitting to the average structure using backbone heavy atoms (C, CA, N).*

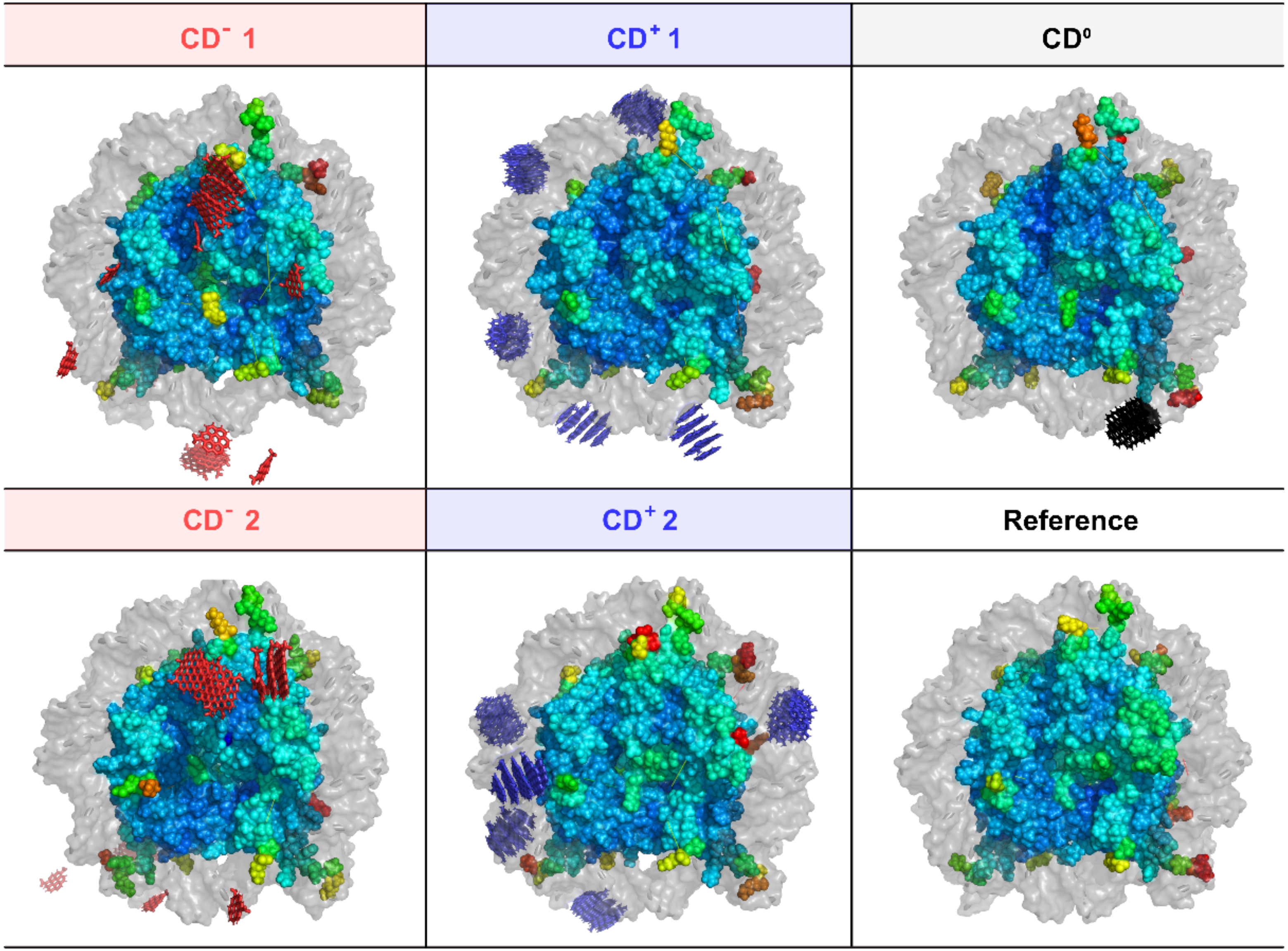

Figure S45: *Top view of the final structure of histones colored by per-residue RMSF* (see Figure S43) *ranging from blue to red (high RMSF).*

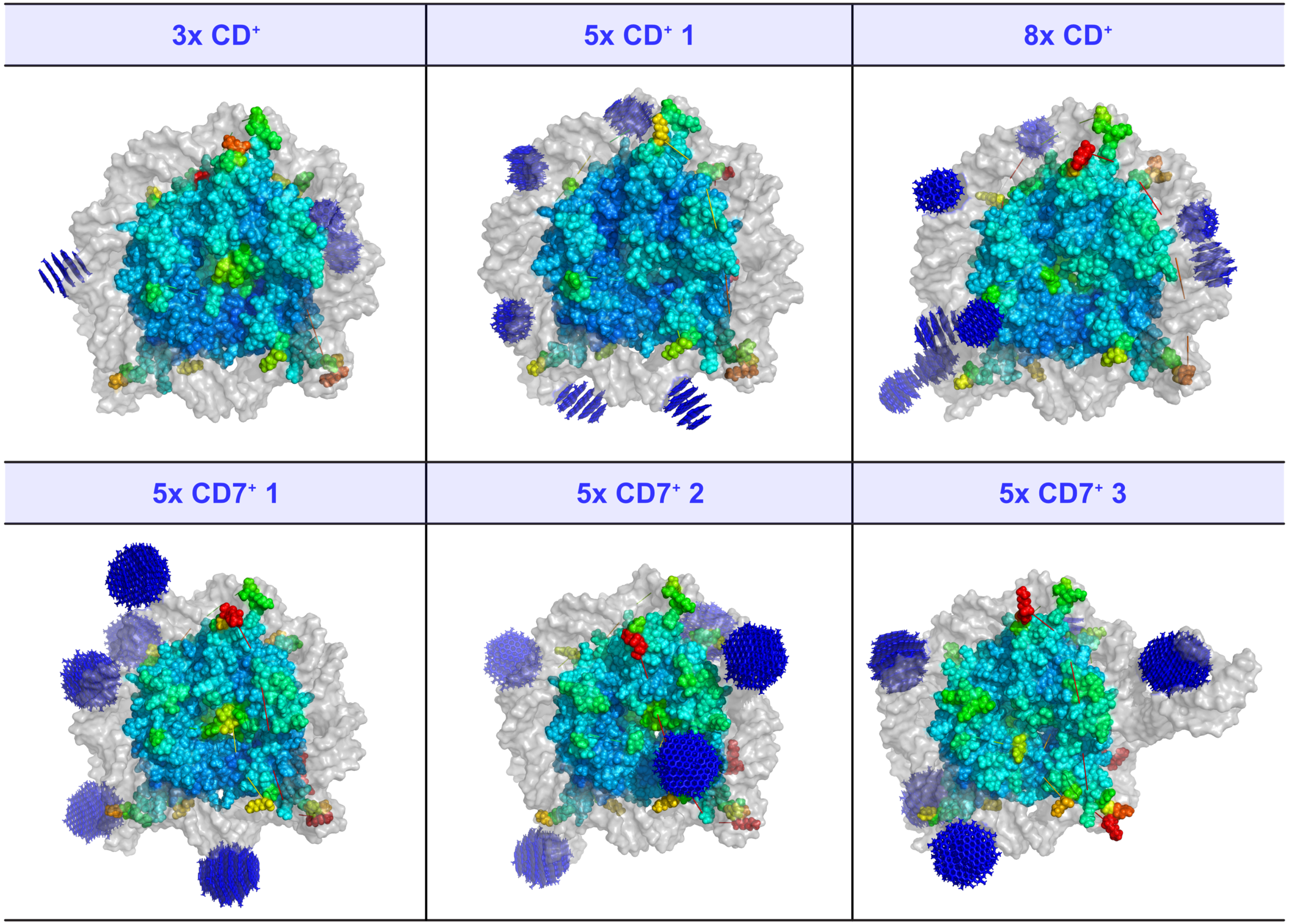

Figure S46: *Top view of the final structure of histones colored by per-residue RMSF (see Figure S44) ranging from blue to red (high RMSF).*

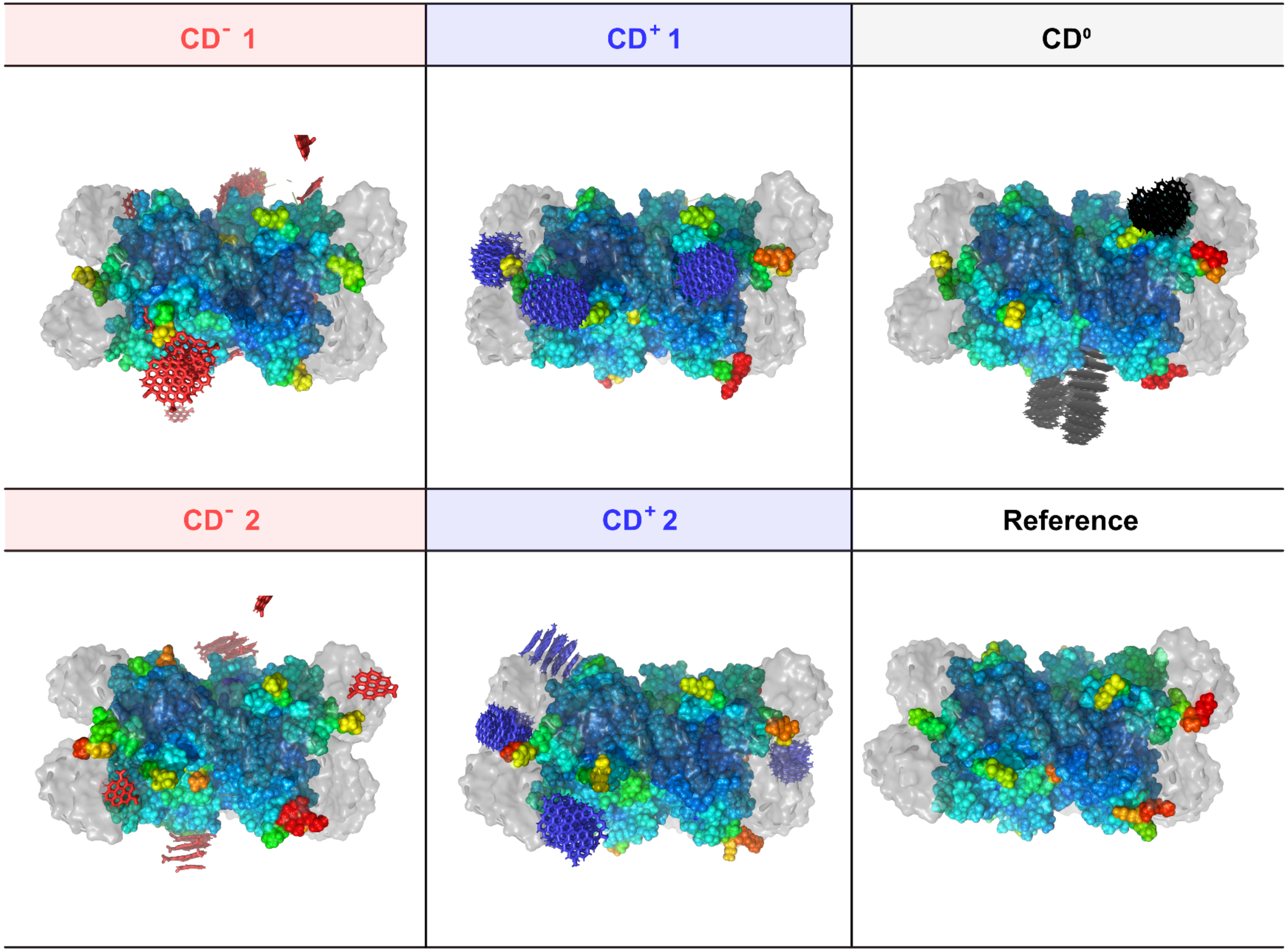

Figure S47: *Dyad view of the final structure of histones colored by per-residue RMSF* *(see Figure S43) ranging from blue to red (high RMSF).*

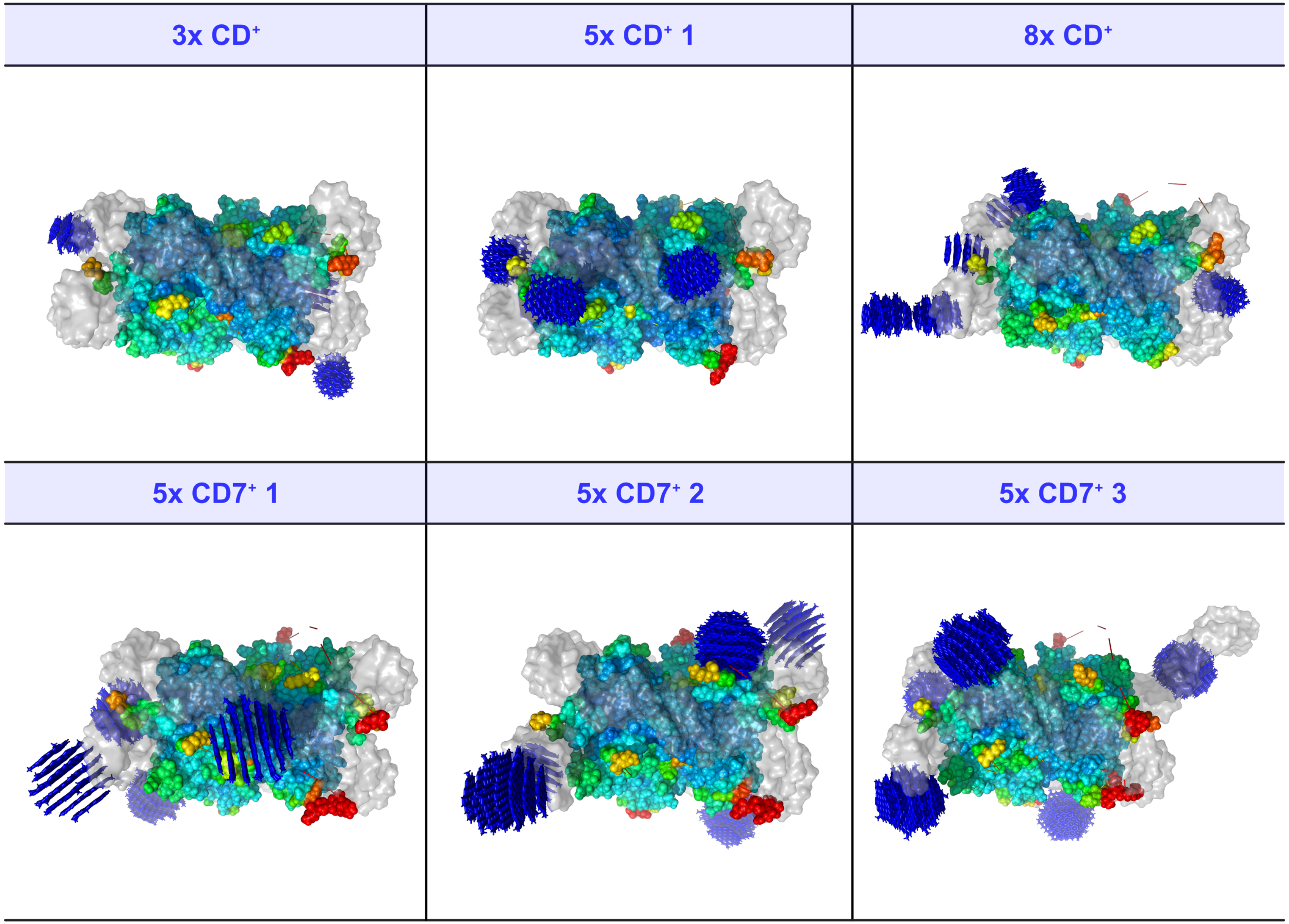

Figure S48: *Dyad view of the final structure of histones colored by per-residue RMSF (see Figure S44) ranging from blue to red (high RMSF).*

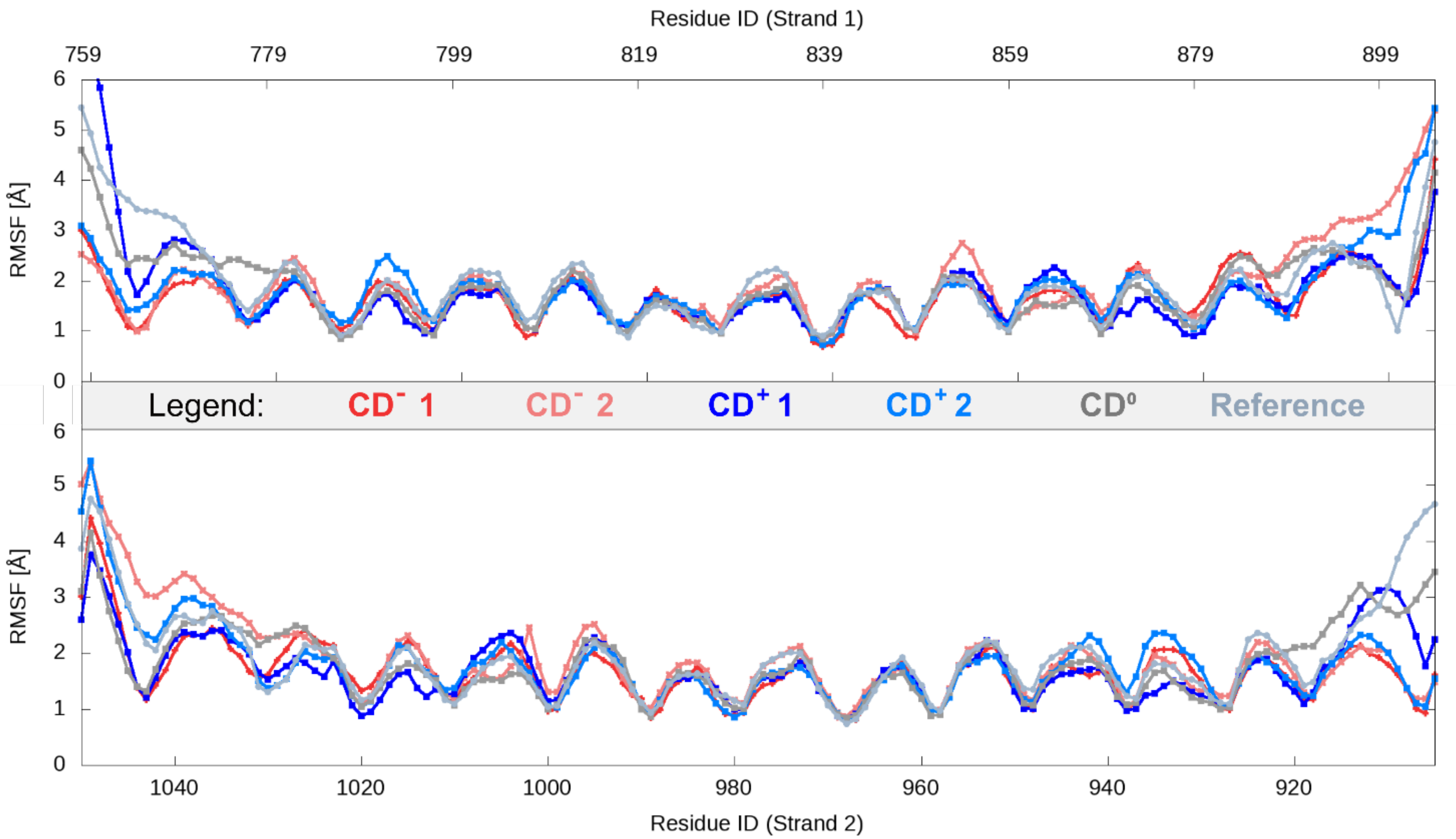

Figure S49: *Per-residue root mean square fluctuations (RMSF) of nucleosomal DNA. RMSF is calculated from the last 100 ns of MD simulation after fitting to the average structure using DNA backbone heavy atoms (C3', C4', C5', O3', O5', P, O1P, O2P). The DNA residues in contact with histone are less flexible than solvent exposed residues.*

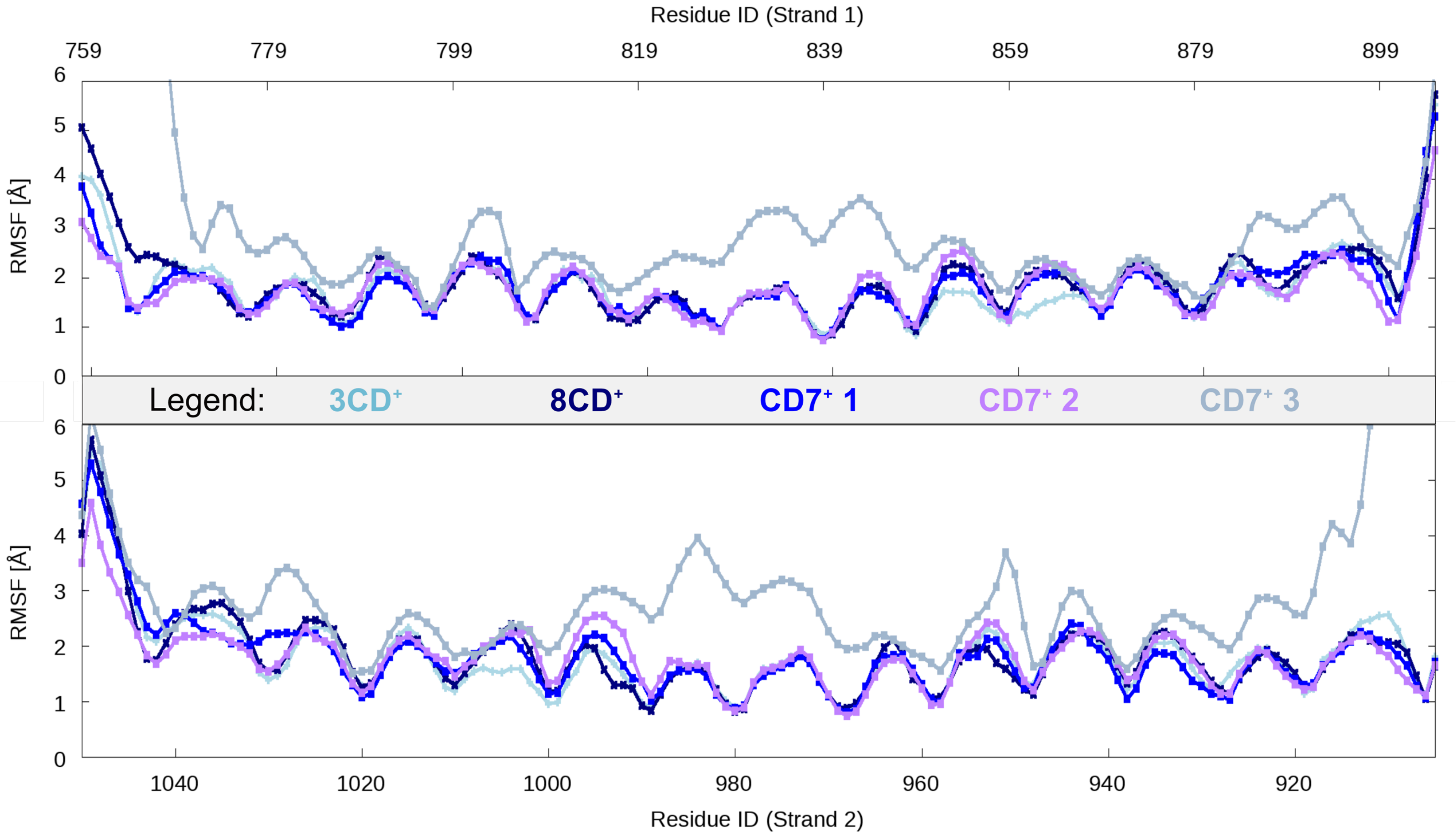

Figure S50: *Per-residue root mean square fluctuations (RMSF) of nucleosomal DNA. RMSF is calculated from the last 100 ns of MD simulation after fitting to the average structure using DNA backbone heavy atoms (C3', C4', C5', O3', O5', P, O1P, O2P). The DNA residues in contact with histone are less flexible than solvent exposed residues.*

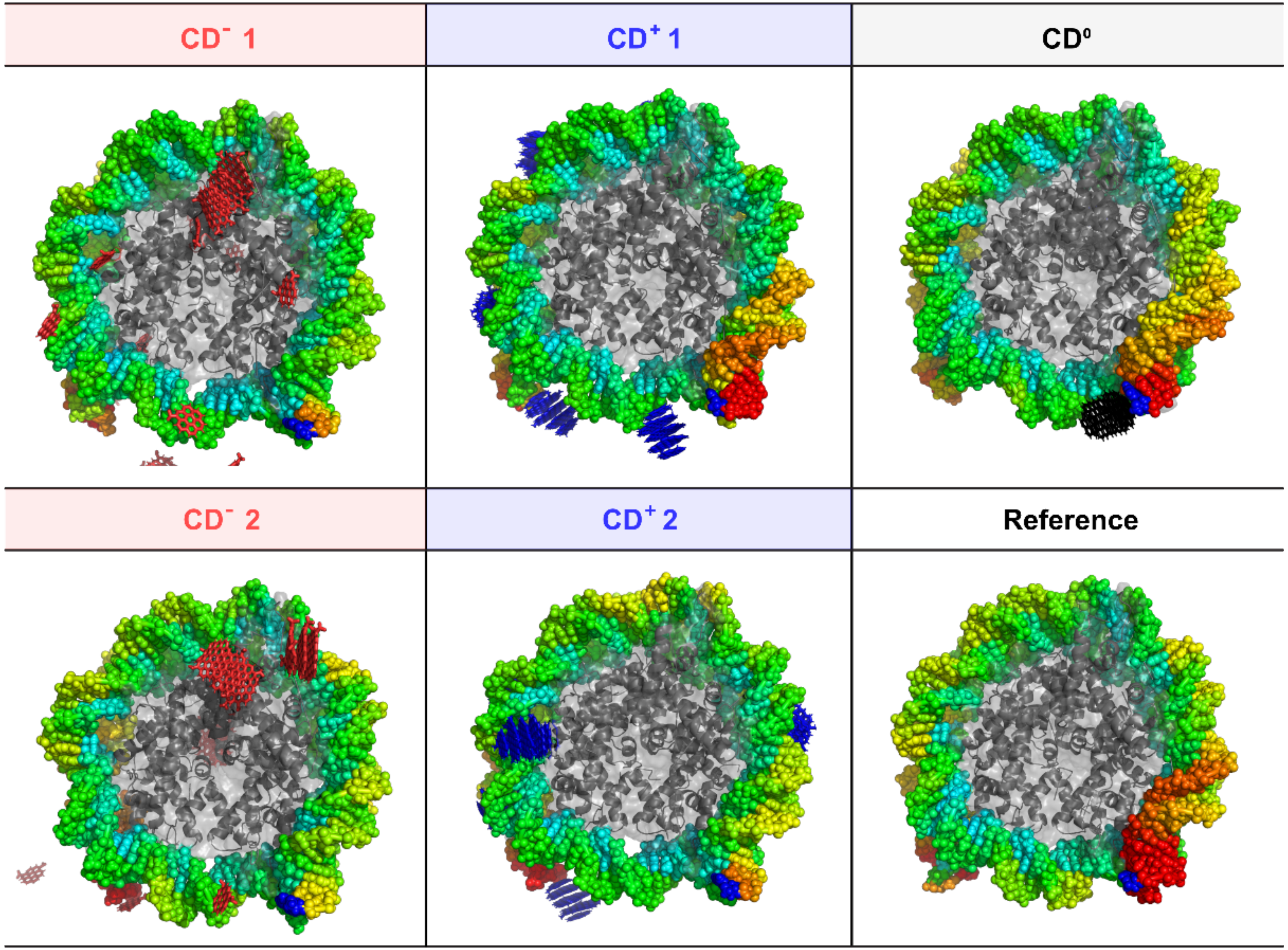

Figure S51: *Top view of the final structure of DNA colored by per-residue RMSF (see Figure S49) ranging from blue to red (high RMSF).*

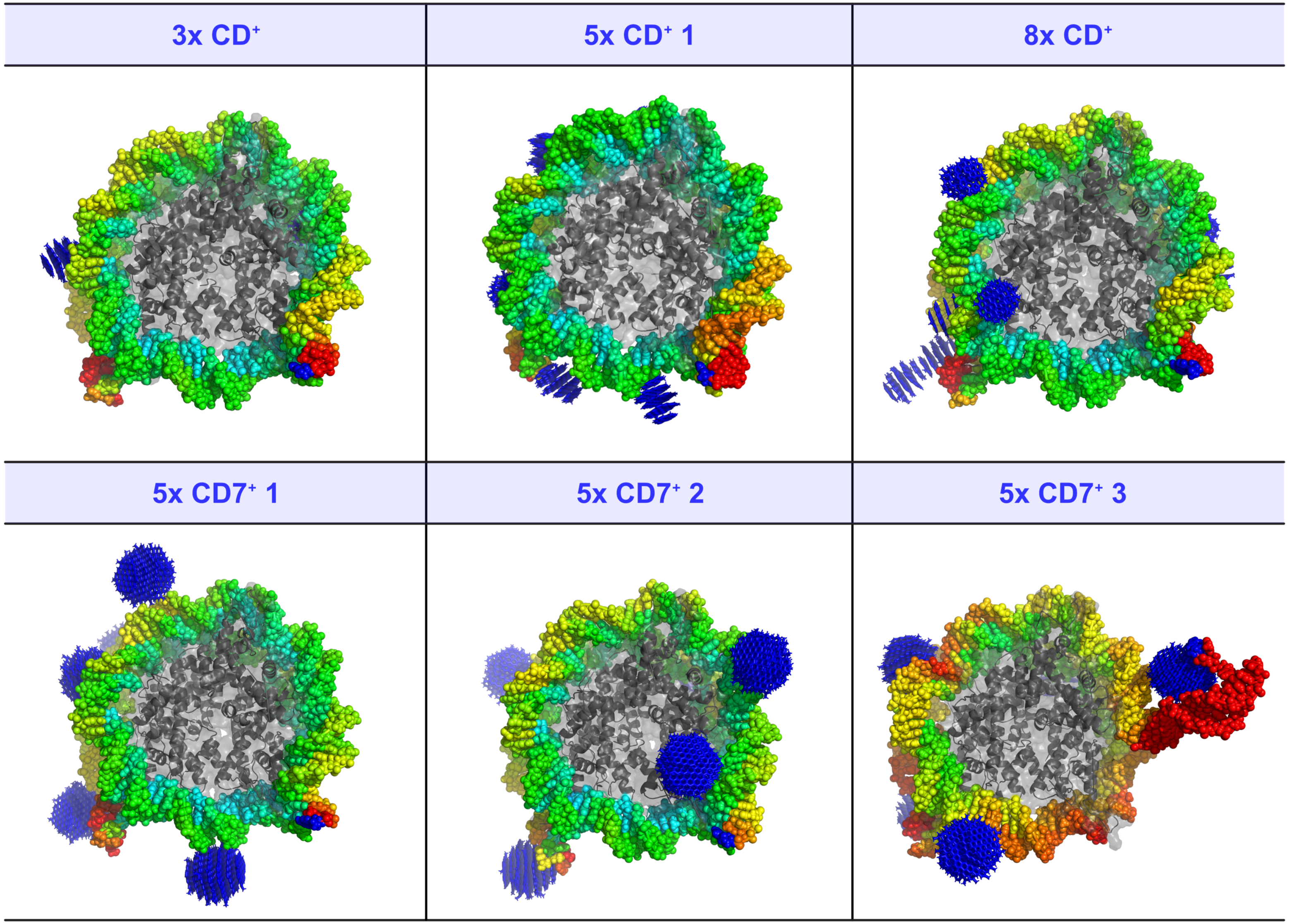

Figure S52: *Top view of the final structure of DNA colored by per-residue RMSF* *(see Figure S50) ranging from blue to red (high RMSF).*

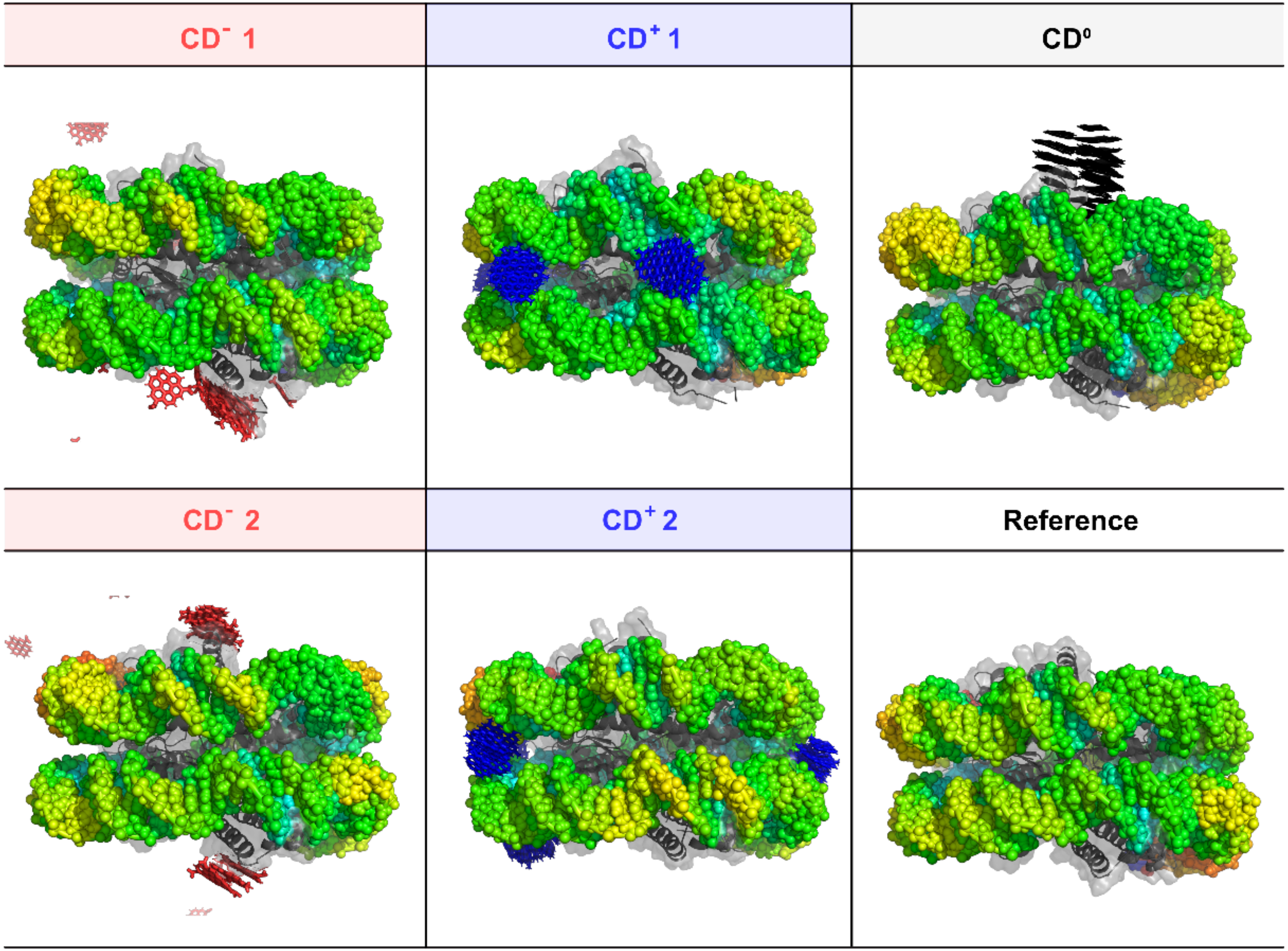

Figure S53: *Opposite the dyad view of NS with DNA colored by per-residue RMSF (see Figure S49) ranging from blue to red (high RMSF).*

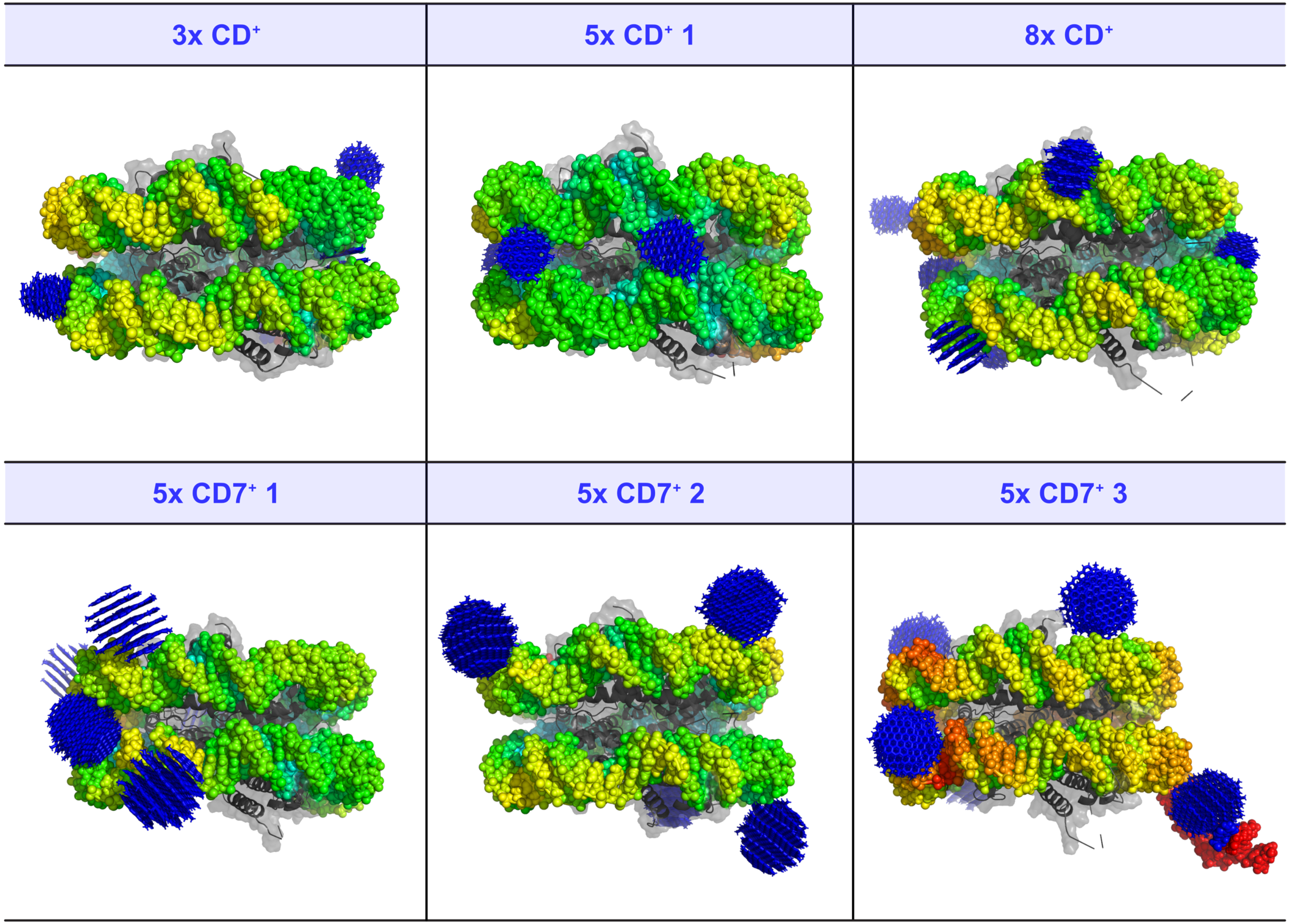

Figure S54: *Opposite the dyad view of NS with DNA colored by per-residue RMSF (see Figure S50) ranging from blue to red (high RMSF).*

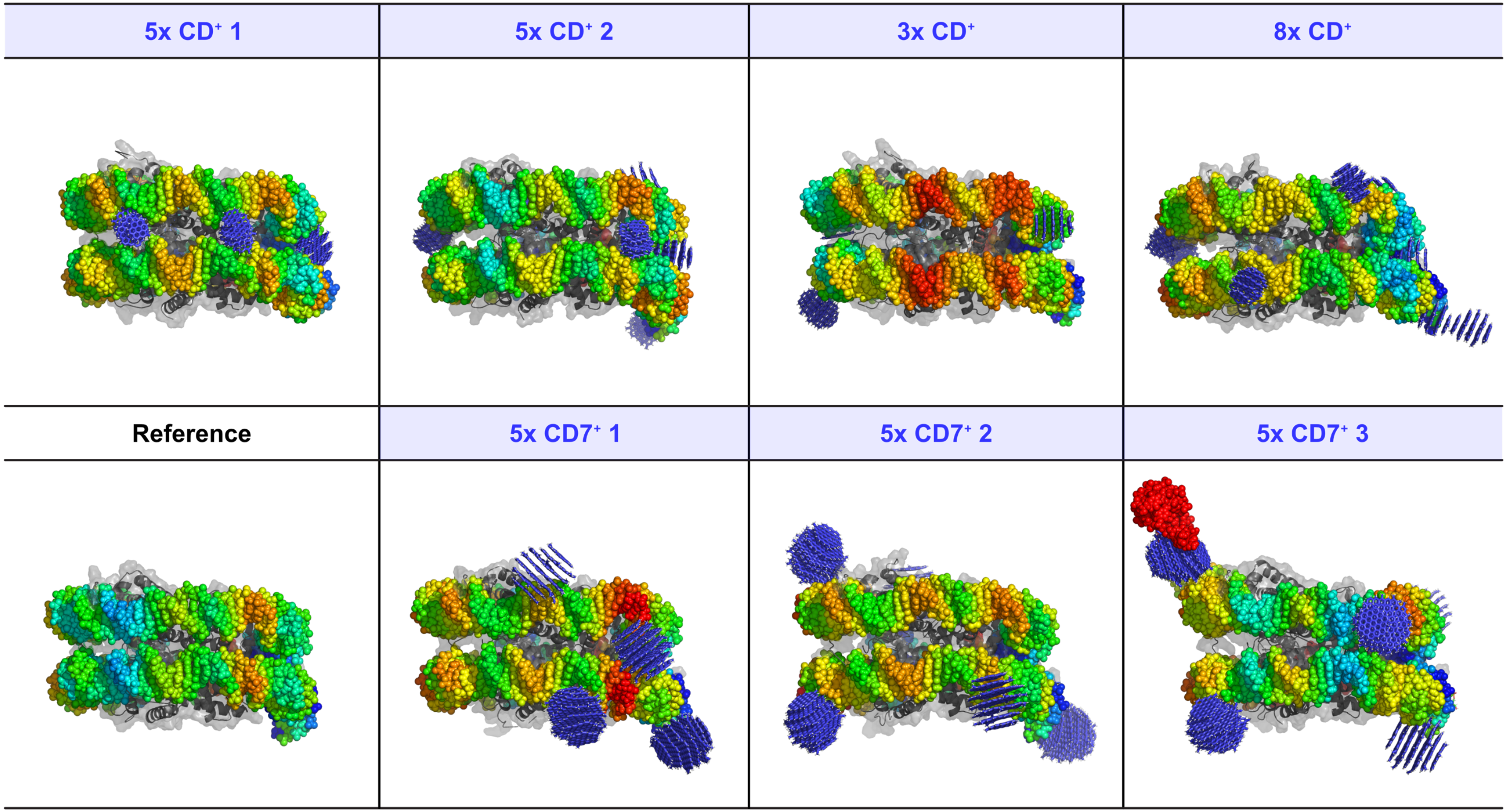

Figure S55: *Opposite the dyad view of NS with DNA colored by distance between DNA gyre centers ranging from blue (<2.0 nm) to red (>3.5 nm).*

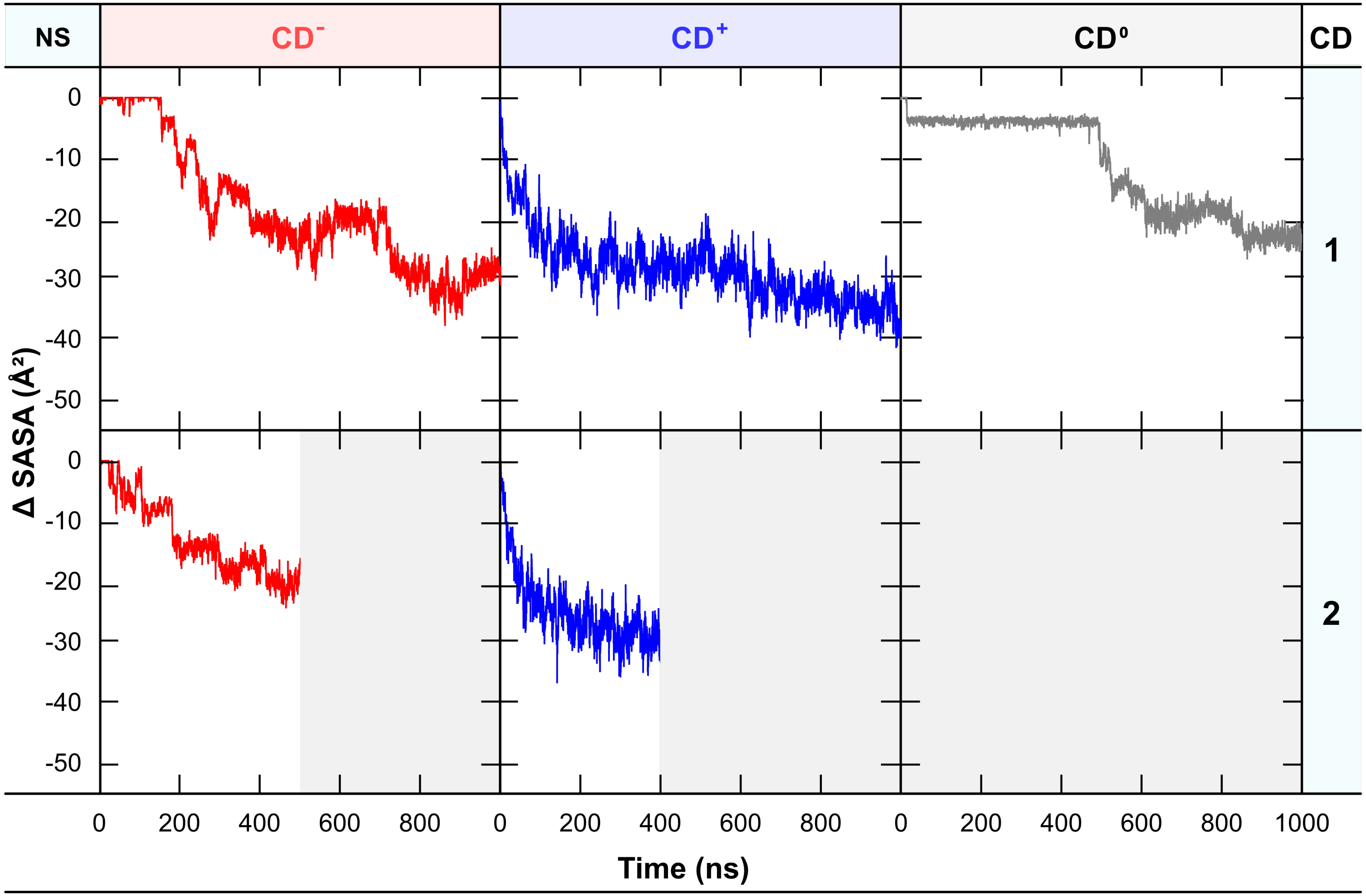

Figure S56: *ΔSASA evolution in* ***NS*** *with five CDs. ΔSASA corresponds to the difference between SASA calculated for the whole NA+CD complex and the sum of SASA of NA and CD separately.*

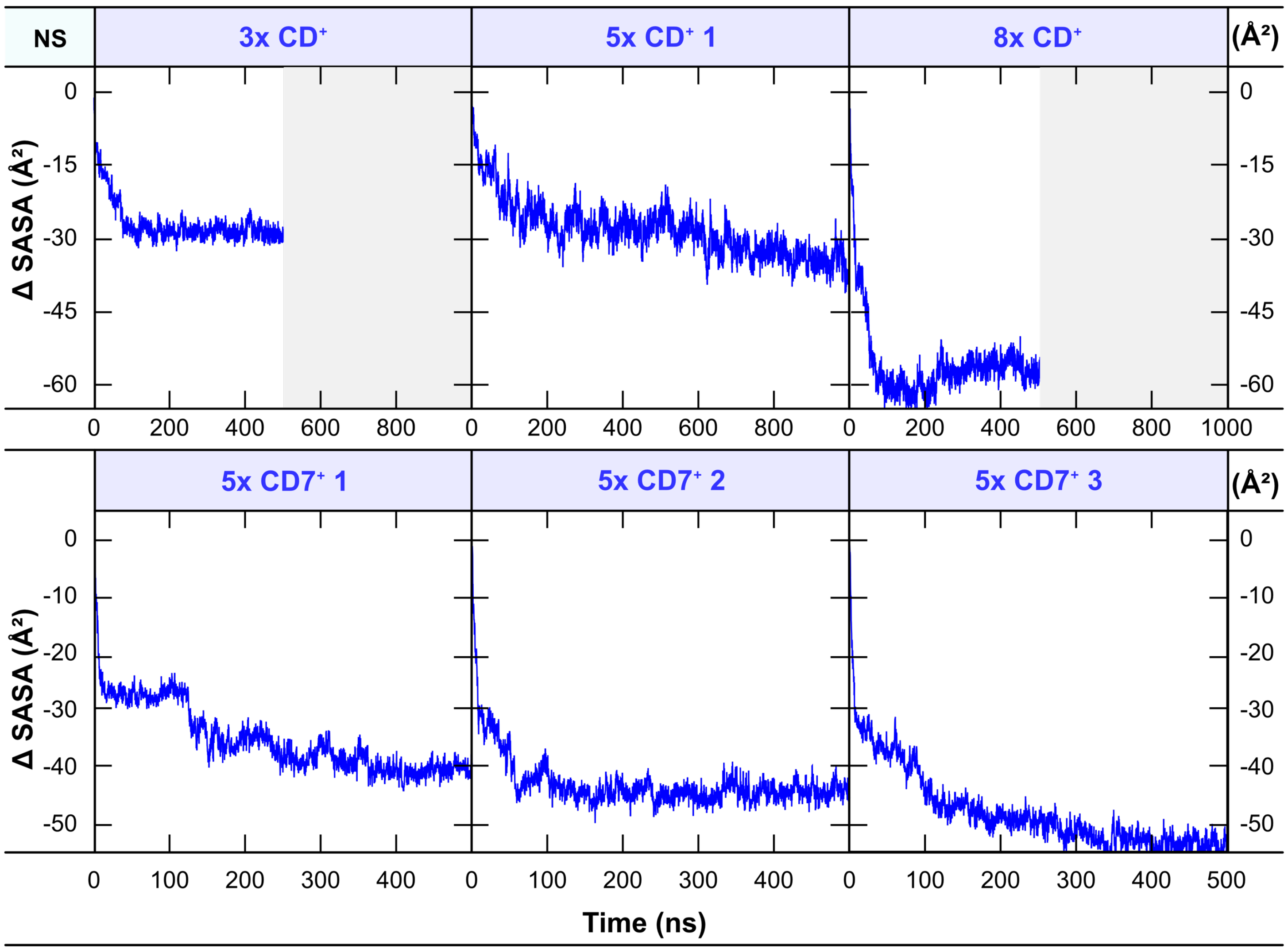

***Figure S57:** ΔSASA evolution in **NS** with varying number and size of CD^+. ΔSASA corresponds to the difference between SASA calculated for the whole NA+CD complex and the sum of SASA of NA and CD separately.*
